# Bacterial Indicators Are Ubiquitous Members of Pelagic Microbiome in Anthropogenically Impacted Coastal Ecosystem

**DOI:** 10.3389/fmicb.2021.765091

**Published:** 2022-01-17

**Authors:** Neža Orel, Eduard Fadeev, Katja Klun, Matjaž Ličer, Tinkara Tinta, Valentina Turk

**Affiliations:** ^1^Marine Biology Station Piran, National Institute of Biology, Piran, Slovenia; ^2^Department of Functional and Evolutionary Ecology, University of Vienna, Vienna, Austria; ^3^Office for Meteorology, Hydrology and Oceanography, Slovenian Environment Agency, Ljubljana, Slovenia

**Keywords:** coastal microbiome, pollution, pathogens, wastewater, *Arcobacter*, microbial source tracking

## Abstract

Coastal zones are exposed to various anthropogenic impacts, such as different types of wastewater pollution, e.g., treated wastewater discharges, leakage from sewage systems, and agricultural and urban runoff. These various inputs can introduce allochthonous organic matter and microbes, including pathogens, into the coastal marine environment. The presence of fecal bacterial indicators in the coastal environment is usually monitored using traditional culture-based methods that, however, fail to detect their uncultured representatives. We have conducted a year-around *in situ* survey of the pelagic microbiome of the dynamic coastal ecosystem, subjected to different anthropogenic pressures to depict the seasonal and spatial dynamics of traditional and alternative fecal bacterial indicators. To provide an insight into the environmental conditions under which bacterial indicators thrive, a suite of environmental factors and bacterial community dynamics were analyzed concurrently. Analyses of 16S rRNA amplicon sequences revealed that the coastal microbiome was primarily structured by seasonal changes regardless of the distance from the wastewater pollution sources. On the other hand, fecal bacterial indicators were not affected by seasons and accounted for up to 34% of the sequence proportion for a given sample. Even more so, traditional fecal indicator bacteria (*Enterobacteriaceae*) and alternative wastewater-associated bacteria (*Lachnospiraceae*, *Ruminococcaceae*, *Arcobacteraceae, Pseudomonadaceae* and *Vibrionaceae*) were part of the core coastal microbiome, i.e., present at all sampling stations. Microbial source tracking and Lagrangian particle tracking, which we employed to assess the potential pollution source, revealed the importance of riverine water as a vector for transmission of allochthonous microbes into the marine system. Further phylogenetic analysis showed that the *Arcobacteraceae* in our data set was affiliated with the pathogenic *Arcobacter cryaerophilus*, suggesting that a potential exposure risk for bacterial pathogens in anthropogenically impacted coastal zones remains. We emphasize that molecular analyses combined with statistical and oceanographic models may provide new insights for environmental health assessment and reveal the potential source and presence of microbial indicators, which are otherwise overlooked by a cultivation approach.

## Introduction

The urbanization and economic development of coastal regions has resulted in an increasing pressure on these environments (Hugo, 2011; Neumann et al., 2015). Anthropogenically induced perturbations can have a major impact on the coastal marine ecosystem, resulting in an impairment of its ecological functions (Nogales et al., 2007, 2011; Chen et al., 2019) and posing a risk to human health and well-being through recreational use of contaminated coastal waters or consumption of contaminated seafood (Stewart et al., 2008; Jahne et al., 2017).

Coastal ecosystems are strongly influenced by land–sea fluxes, related to various anthropogenic inputs either from non-point pollution sources (e.g., agricultural drainage water, urban stormwater runoff, atmospheric deposition, sewage from ships, wildlife, leakage from sewage systems) or point pollution sources (e.g., rivers, wastewater outfalls). All these various inputs introduce mixtures of inorganic/organic chemical compounds (Imai et al., 2002; Kontas et al., 2004; Raymond and Spencer, 2015; Wang and Chen, 2018; Malone et al., 2021) and microorganisms, including pathogens (Naidoo and Olaniran, 2013; Buccheri et al., 2019; Numberger et al., 2019), into the coastal marine ecosystems. These inputs are shown to play a key role in processes that negatively affect the quality of water and ecosystem, such as eutrophication (Boesch et al., 2001), decrease in dissolved oxygen rates (Breitburg et al., 2018), and shifts in bacterioplankton communities (Caron et al., 2017; Andersson et al., 2018; Rodríguez et al., 2018; Lønborg et al., 2019). Furthermore, wastewater-borne microbial taxa are linked to infections and mass mortalities of different ecologically and economically important marine species (Sutherland et al., 2010; Ziegler et al., 2016).

Despite recent improvements, wastewater treatment plants are still one of the main sources of anthropogenic pollution to coastal ecosystems (Naidoo and Olaniran, 2013; Atashgahi et al., 2015; Chu et al., 2018; Aghalari et al., 2020). Untreated or treated wastewaters are often discharged into the marine environment *via* estuaries, and their dilution is strongly regulated by river flow rate, density gradient, currents, tides, etc. (Mandac and Faganeli, 2015; Álvarez-Vázquez et al., 2016; Sun et al., 2020). Alternatively, treated wastewater might be discharged directly into the marine environment *via* submarine outfalls, in which low-density effluent rises to the surface and gets diluted along the water column (Malačič and Mozetič, 2005; Mozetič et al., 2008).

The use of bacterial indicators is widely employed in ecosystem monitoring to assess the state of the environment and to identify sources of contamination. For example, standardized culture-based approaches (e.g., Directive, 2006/7/EC), which detect the presence and abundance of fecal bacteria (e.g., *Escherichia coli*, enterococci), are used to identify fecal contamination and associated health risks (Scott et al., 2002). However, such culture-based approaches are strongly limited to the detection of culturable bacteria that comprise only a small portion of the total allochthonous and indigenous bacteria in the marine environment (Stewart et al., 2008; Converse et al., 2011). The development of high-throughput sequencing technologies has expanded the inventory of taxonomic groups suitable as indicators for identifying pollution and characterizing human health risks (reviewed in McLellan and Eren, 2014; Caruso et al., 2016; Cordier et al., 2020). Therefore, numerous studies proposed a list of alternative feces- and sewage-associated microbial indicators, affiliated to *Bacteroidaceae*, *Clostridiaceae*, *Lachnospiraceae, Ruminococcaceae, Acinetobacter*, *Arcobacteraceae, Aeromonadaceae*, and *Legionellaceae* (Maugeri et al., 2004; Stewart et al., 2008; Luna et al., 2016; Buccheri et al., 2019; Numberger et al., 2019). In addition, computational techniques, such as microbial source tracking, enable the identification of pollution sources that introduce allochthonous microbes into the marine environment (Neave et al., 2014; Henry et al., 2016; Saarenheimo et al., 2017; Brown et al., 2019).

Previous efforts to understand the effects of wastewater pollution on coastal marine ecosystems mainly focus on the sediment microbiome (Korlević et al., 2015; Quero et al., 2015; Hassard et al., 2016; Luna et al., 2016; Chen et al., 2019). Indeed, a large proportion of contaminants, including allochthonous microorganisms, can associate with particular matter and are most likely transported into the sediment (Hassard et al., 2016). It is reported that sediments in urbanized areas have higher concentrations of fecal or sewage microbial indicators (Lu and Lu, 2014; Luna et al., 2016), including human pathogens, groups associated with specific metabolic potentials (Chen et al., 2019), and the presence of antibiotic-resistant genes (ARGs) (Liang et al., 2020; Su et al., 2020; Ahmed et al., 2021). However, sediment resuspension is an important mechanism contributing to the flux of nutrients and microbiome into the water column (Giani et al., 2001; Pusceddu et al., 2005; Perkins et al., 2014), in particular in semienclosed coastal systems (Wang et al., 2007). Besides this, wastewater is predominantly released into the water column (Hylland and Vethaak, 2012). However, the potential impact of anthropogenic perturbations on the pelagic microbiome in coastal ecosystems gained attention only recently (Paliaga et al., 2017; Pearman et al., 2018).

In our study, we investigated the pelagic microbiome of an anthropogenically impacted coastal ecosystem with a focus on the bacterial indicators within. Our model system, the Gulf of Trieste (Northern Adriatic), is influenced by various pollution sources that strongly affect the sanitary quality and ecology of this area. Previous studies report increased concentrations of fecal coliforms (Turk et al., 2007; Luna et al., 2018) and the presence of human enteric viruses (Gonçalves et al., 2018) as well as an increase in phytoplankton biomass (Mozetič et al., 2008, 2012) near pollution sources, such as wastewater discharges and estuaries. Thus, we hypothesized that wastewater may affect the structure of the coastal pelagic microbiome, being an important source of wastewater-borne bacterial indicators (hereinafter bacteria belonging to traditional fecal indicators or alternative indicators of wastewater pollution). To test our hypothesis, we conducted seasonal sampling at high spatial resolution along the dilution gradient of two pollution sources: i.e., a wastewater-contaminated estuary and a submarine outfall. We assessed the dynamics of microbial community composition in relation to environmental factors, focusing on bacterial indicators associated with wastewater pollution, by combining high-throughput sequencing of 16S rRNA genes with analysis of various physicochemical parameters. Finally, the spatial extent of wastewater pollution was estimated using a combination of Lagrangian particle tracking model and microbial source tracking. The Lagrangian particle tracking approach, which is routinely employed to estimate marine particles drifts in the marine environment (Vennell et al., 2021), was applied to predict the propagation of pollution based on oceanographic conditions, whereas a tool SourceTracker (Knights et al., 2011) was implemented to estimate the proportion of pollution-associated microbes in bacterioplankton communities.

## Materials and Methods

### Study Site

**FIGURE 1:**
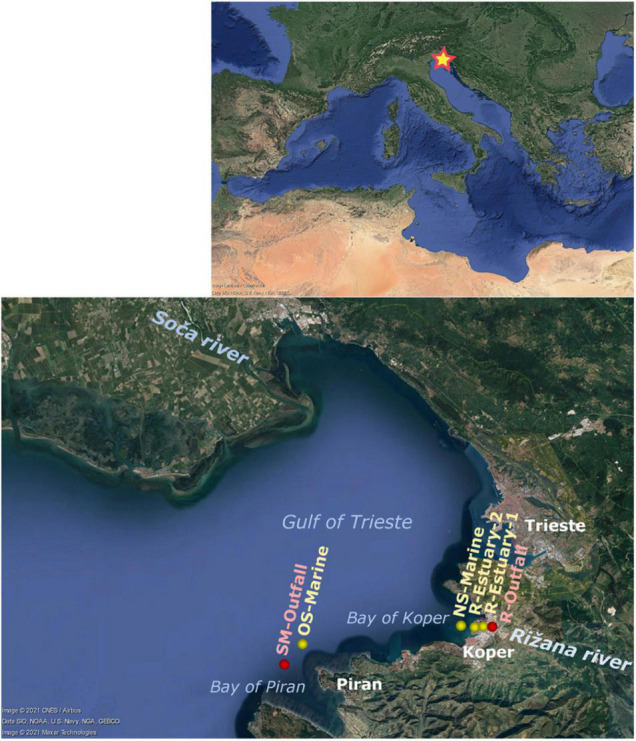
Map of the Mediterranean Sea showing sampling stations in the Bay of Koper and the Bay of Piran (Gulf of Trieste, northern Adriatic Sea): submarine outfall (SM-Outfall), offshore marine (OS-Marine), nearshore marine (NS-Marine), Rižana estuary 2 (R-Estuary-2), Rižana estuary 1 (R-Estuary-1), Rižana outfall (R-Outfall). Study area is subject to anthropogenic pressures: wastewater inputs (SM-Outfall = submarine outfall of WWTP Piran, R-Outfall = outfall from WWTP Koper), urban areas (Trieste, Koper and Piran) and port of Koper (located in Bay of Koper). Study area is presented with Google Earth image (Google Earth Pro 7.3.4.8248: Image © 2021 CNES/Airbus; Data SIO, NOAA, U.S. Navy, NGA, GEBCO; Image © 2021 Maxar Technologies).

The Gulf of Trieste is a shallow (<25 m depth), semienclosed, river-dominated ecosystem in the northernmost part of the Adriatic Sea. Local meteorological conditions, freshwater inputs, and exchange of seawater from the southern Adriatic induce pronounced seasonal variability of seawater temperature (9°C–25°C) and salinity (32–38) (Malačič et al., 2006). Water column conditions change from stratification in summer to a well-mixed water column in winter (Malačič et al., 2012). River discharges, mainly regulated by precipitation with peaks in early spring and autumn, importantly influence nutrient dynamics in this area (Klun et al., 2019). The greatest discharge source is the Soča (Isonzo) River, whereas minor rivers along the southern Slovenian coast, such as Rižana, have an impact on turbidity, freshwater, and nutrient content at a smaller spatial scale (Faganeli and Turk, 1989; Mandac and Faganeli, 2015; Cozzi et al., 2020; Figure 1). In recent years, a decrease in river loads of nutrients was observed, indicating that other allochthonous sources, such as wastewater loads, might become more important for this system (Cozzi et al., 2012).

Besides the influences of port activities and flushing from the land, the river Rižana, located in the eastern part of the Bay of Koper, is an important source of freshwater and various types of pollution in our study area (Figure 1). The most important pollution source is wastewater discharge into the river Rižana from the wastewater treatment plant (WWTP) Koper that receives domestic and industrial wastewater of the adjacent cities and the waste of Izola General Hospital (84,500 PE—population equivalent). For example, in the year 2019, the WWTP Koper discharged 5.9 × 10^6^ m^3^ of tertiary treated wastewater, resulting in loads of 7.9 t of total phosphorus and 17.4 t of total nitrogen (ARSO, 2021). The Rižana river flow rate is from 0.07 and 63.2 m^3^ s^–1^ and is strongly dependent on the season and weather conditions (Petrič et al., 2020). The mouth of the river is located in the basin of the port of Koper, and the whole estuary is a dredged canal with an average depth of 16 m, a highly stratified area with high variations in temperature, salinity, density, and turbidity (Mandac and Faganeli, 2015). Estuarine water circulation and distribution of freshwater within the Bay of Koper depends on meteorological and hydrodynamic conditions (river discharge rate, tides, and wind). Another important pollution source in our study area is the submarine wastewater outfall (Bay of Piran) of the WWTP Piran (30,000 PE), where sewage is released *via* two submarine pipes located 3,600 m from the shore with diffusers at the end (Figure 1). In 2019, the WWTP Piran discharged 2.2 × 10^6^ m^3^ of tertiary treated wastewater, resulting in a load of 2.1 t of total phosphorus and 12.9 t of total nitrogen (ARSO, 2021).

### Sampling

In our study, we performed sampling at six stations in the eastern part of the Gulf of Trieste (Figure 1), representing sites with different distances from the two pollution sources in focus (i.e., the Rižana estuary and the submarine outfall), and the offshore marine station, from which we expected a lower anthropogenic impact (Turk et al., 2007; Cozzi et al., 2012). Seawater samples were collected seasonally, four times during the first sampling period from March 2015 to February 2016 and four times during the second sampling period from December 2018 to October 2019 (Supplementary Table 2). During the first sampling campaign in 2015/2016, samples were collected at three stations: in the mouth of the river Rižana, after the location of the wastewater outlet of the WWTP Koper (R-Outfall), in the inner part of the estuary (R-Estuary-1), and at the nearshore station in the middle of the Bay of Koper, 2 km from the river mouth (NS-Marine) (Figure 1). A second sampling, performed in 2018/2019, included the same sampling stations along the transect from the inner part of the estuary (R-Estuary-1) and in the middle of the Bay of Koper (NS-Marine) (Figure 1 and Supplementary Table 2). At the same time, additional sampling was performed in the middle of the estuary (R-Estuary-2) and in the Bay of Piran, at the location of the submarine outfall (SM-Outfall) of WWTP Piran, and at an offshore marine station (OS-Marine) at the location of oceanographic buoy Vida, located approximately 15 km away from the river estuary, that is less subjected to anthropogenic pressures (Figure 1 and Supplementary Table 2).

At each station, temperature, salinity, and dissolved oxygen were measured throughout the water column before each sampling, using a CTD fine-scale probe (Microstructure Profiler MSS90, Sea & Sun Technology GmbH). Seawater was collected with 5 L Niskin bottles at two different depths: ∼0.5 m below the surface and ∼0.5 m above the bottom. Water samples were stored in Nalgene bottles (1 M HCl and MilliQ prewashed) and immediately transported to the laboratory protected from light. Seawater samples were subsampled for microbiological analyses (1) to determine the abundance, production, and composition of the microbial community; (2) to determine the abundance of total coliform bacteria using standard cultivation-based techniques to monitor fecal contamination of seawater. At the same time, the subsamples were collected for chemical analyses (i.e., dissolved organic and inorganic nutrients).

Due to missing environmental data and lower spatial resolution in the 2015/2016 sampling, the results were not analyzed together with the main 2018/2019 data set. However, separate complementary analyses were performed on the 2015/2016 data and were used for comparison in the bacterial indicator analyses.

### Chemical Parameters

Samples for dissolved organic carbon (DOC) and total dissolved nitrogen (TDN) were filtered onto combusted Whatman GF/F filters (∼0.8 μm pore size) and immediately stored at −20°C until analysis. DOC and TDN analyses were performed by a high-temperature catalytic method using a Shimadzu TOC-L analyzer equipped with a total nitrogen unit. Calibration for non-purgeable organic carbon (NPOC) was done with potassium phthalate, and potassium nitrate was used for TDN. Results were validated with Surface Seawater Reference (SSR) material for DOC and TDN (CRM Program, Hansell Lab). Reproducibility was no greater than 2% (Hansell and Carlson, 1998).

Dissolved inorganic nitrogen (NH_4_^+^, NO_2_^–^, NO_3_^–^) and dissolved inorganic phosphorus (PO_4_^3–^) concentrations were determined spectrophotometrically using segmented flow analysis (QuAAtro, Seal Analytical) following standard methods (Hansen and Koroleff, 2007). Validation and accuracy of results were checked with reference material (KANSO Co., Ltd.) before and after sample analyses. Quality control is performed annually through participation in an intercalibration program (QUASIMEME Laboratory Performance Study).

### Bacterial Abundance and Production

The subsamples for bacterial cell counts by flow cytometer were fixed with glutaraldehyde (2.5% final concentration) and stored at −80°C until further analysis. Samples were kept at room temperature for 30 min before staining with SYBR Green I (Invitrogen™, Life Technologies) at a 1 × concentration (10,000 × final dilution of DMSO stock solution), and 50 μL of each 400 μL sample was analyzed with flow cytometer Accuri C6 (BD) (Gatza et al., 2013), technical triplicate was measured for each sample.

Bacterial carbon production (BCP) was measured using the ^3^H-leucine incorporation method (20 nM final concentration, Perkin-Elmer), employing the centrifugation protocol of Smith and Azam (1992). BCP was calculated as described by Simon and Azam (1989) using 19.8 fg C bacterium^–1^ as a conversion factor (Lee and Fuhrman, 1987).

### Total Coliform Bacteria

Sample aliquots of 100 and 50 mL were filtered onto a 0.22 μm mixed cellulose membrane filter (47 mm, Millipore). Filters were placed on mFC agar (Merck, Millipore) and incubated at 35°C for 12 h in the dark. Blue colonies were counted, and the result was expressed as the number of colony-forming units (CFU) per 100 mL of seawater.

### Statistical Analysis of Environmental Data

Data handling and statistical analyses of the environmental data were performed in R (v. 4.0.1.) in RStudio (v. 1.2.5019, RStudio Team, 2020), using the *“tidyverse”* (Wickham et al., 2019), *“rstatix” (Kassambara, 2021)*, and *“vegan”* (Oksanen et al., 2020) packages. A test for a significant difference of selected environmental variables between sampling stations, season, or depth was performed by one-way analysis of variance (ANOVA) or Kruskal–Wallis test. The test was selected based on a previous analysis of normality using the Shapiro–Wilk method. The presence of a significant difference between sample groups is indicated with a significance level of at least *p* > 0.05. *Post hoc* analyses were performed using the Tukey test after one-way ANOVA or pairwise comparison using the Wilcoxon test.

Relationships between environmental parameters were studied using the Pearson correlation, performed on scaled (root-mean-square, non-centered) values of environmental parameters.

### Bacterial Community Analysis Using Illumina Sequencing of the 16S rRNA Gene

A large volume of seawater (500 mL–1 L) was filtered onto 0.2 μm polyethersulphone filters (PALL Life Sciences), which were stored at −80°C until further procedure. DNA was extracted from the filters with phenol-chloroform extraction according to Angel (2012) with slight modifications as described in Bayer et al. (2019). Amplification and sequencing were performed by LGC Genomics GmbH (Berlin). Primers 341F (5′-CCT ACG GGN GGC WGC AG-3′) and 785R (5′-GAC TAC HVG GGT ATC TAA KCC-3′) (Klindworth et al., 2013) were used for amplification of the V3-V4 region of bacterial 16S rRNA gene. The amplicons were sequenced on the Illumina Miseq platform (2 bp × 300 bp pair-end).

Primer sequences were removed using Cutadapt 2.1 (Martin, 2011), and all further analyses were conducted in R (4.0.1.). Paired-end reads were trimmed, denoised, and merged using the *“DADA2”* package (Callahan et al., 2017). Forward and reverse reads were quality trimmed to 230 (forward) and 220 bp (reverse) at a maximum expected error rate of 2. After merging of paired-end reads with a minimum overlap of 10 bp, chimeras were removed with method *“consensus.”* Only amplicon sequencing variants (ASVs) occurring at least twice and with a length between 400 and 430 were kept for further analyses. Taxonomy was assigned using the SILVA database (SSU 138) (Quast et al., 2013).

Further general manipulations were performed using *“phyloseq”* (McMurdie and Holmes, 2013). ASVs assigned to Eukaryota, chloroplast, Mitochondria, and Archaea or not assigned at the phylum level were excluded. The *“iNEXT”* (Hsieh et al., 2016) package was used to make rarefaction curves for each sample. Graphs were plotted with the *“ggplot2”* package (Gómez-Rubio, 2017). The number of observed ASVs, Chao1, Shannon, and inverse Simpson indices were calculated through the *“phyloseq”* package to describe alpha diversity. Phyla with a total abundance of less than 100 were removed for further analyses. Non-metric multidimensional scaling of bacterial community composition was assessed on variance stabilized ASV abundance data based on the Euclidean distance matrix. Additionally, permutational multivariate analysis of variance between the groups of samples (ADONIS) in R package *“vegan” (Oksanen et al., 2020)* was used to test significance between the groups of samples. RDA (redundancy analysis) in R package *“vegan”* was used to identify drivers of bacterial community composition in the samples. The analysis was performed on variance stabilized data. For variable reduction, we identified the environmental variables that have the strongest impact on community composition using the “*ordistep”* function with both-way selection (999 permutations, 100 steps). Variation partitioning was performed to test the significance of the individual contribution of the variables with the strongest impact. Shared ASVs were visualized with *“nVennR”* (Quesada, 2021).

### Bacterial Indicators

We searched for ASVs identified as traditional fecal indicators (i.e., ASVs belonging to the family *Enterobacteriaceae*, including *Escherichia* species and *Enterococcaceae*, including the genus *Enterococcus*) and alternative indicator taxa for wastewater pollution proposed by different studies (Supplementary Table 1). The selection was performed at the family level. The analyses and statistics performed on this data set were the same as those described for the whole community.

### Characterization of Arcobacter Diversity

Sequences assigned to the family *Arcobacteraceae* were extracted from the 2015/2016 and 2018/2019 data sets and uploaded to the Silva online tool for alignment, classification, and tree service (SINA 1.2.11:^1^). We searched for the 10 closest neighbors with a minimum identity of 0.97. The phylogenetic tree was computed *de novo* including neighbors, using the “Compute tree” option (program: RAxML, model: GTR). The tree was exported as a Newick file and visualized and connected with metadata using iTOL (Letunic and Bork, 2019).

### Microbial Source Tracking

Based on the assumption that microbial communities are spatially distributed with water mixing, a Bayesian community-based microbial source tracking algorithm “SourceTracker” (v1.0) (Knights et al., 2011) was applied on the ASV abundance table to identify the extent at which wastewater microbes (i.e., sources communities) are distributed in the environment (i.e., sink communities). The SourceTracker approach models the potential presence of a mixture of source communities in sink communities, in which the mixing proportions of the different sources are unknown. All samples were randomly subsampled to 5,000 sequences prior to the analysis, which was conducted according to default conditions, defined by the developers: burn-in period—100, restarts—10, dirichlet hyperparameters (α, β)—0.001 [the algorithm and its configuration are described in detail in Knights et al. (2011)].

### Pollution Propagation Trajectories Using Oceanographic Models

Lagrangian backtracking simulations were used, at each sampling time point, to backtrack a total of 1,280 virtual particles from each sampled station to the pollution source at the Rižana river. The Lagrangian modeling engine used in this study was the OceanDrift module of the OpenDrift modeling environment that is described in detail by Dagestad et al. (2018). Ocean currents from the innermost ocean model setup (over the Gulf of Trieste) and winds from the ALADIN SI atmospheric model were employed as inputs for Lagrangian backtracking of water masses. Lagrangian back-propagation, i.e., upwind and upstream advection backward in time, was employed to determine the origin of sampled water masses at each sampling station in the days prior to the sampling.

Atmospheric conditions over the Gulf of Trieste around the sampling dates were simulated using an operational version of the ALADIN SI atmospheric model at the Slovenian Weather Service. Further details about atmospheric and ocean model configurations are available in the Supplementary Material (Supplementary Text 1) and in Strajnar et al. (2015, 2019) and Ličer et al. (2016, 2020).

## Results

### Seasonal and Spatial Dynamics of the Pelagic Microbiome in Relation to Environmental Conditions

**FIGURE 2:**
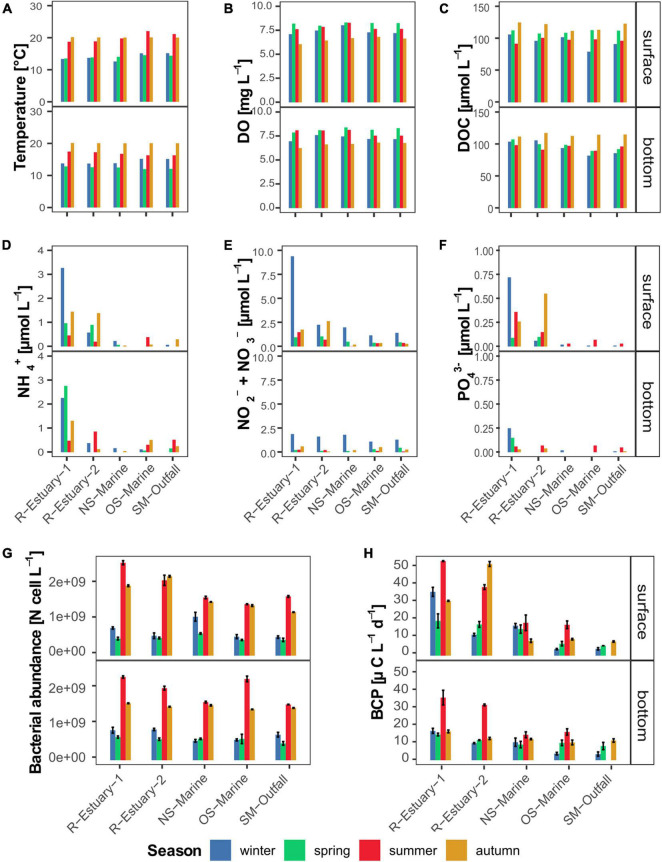
Dynamics of selected environmental parameters at the surface and bottom layer in winter, spring, summer, and autumn during the 2018/2019 survey in the eastern part of the Gulf of Trieste (sampling stations: R-Estuary-1, R-Estuary-2, NS-Marine, OS-Marine and SM-Outfall). **(A)** Temperature; **(B)** dissolved oxygen; **(C)** dissolved organic carbon: **(D)** ammonium; **(E)** nitrate and nitrite; **(F)** orthophosphate; **(G)** bacterial abundance; **(H)** bacterial carbon production. Data for bacterial carbon production at SM-outfall in summer are missing.

Pronounced seasonal changes in the physical conditions of the seawater were observed at all sampling stations during our 2018/2019 *in situ* survey. Average seawater temperature ranged from 13°C in spring to 20°C in autumn. Small spatial differences in seawater temperature were observed at the surface in summer; otherwise, no horizontal spatial trend was observed (Figure 2A). The difference between surface and bottom temperatures was observed during summer sampling (Figure 2A). Seasonal variation was also observed in salinity with lower salinity recorded in winter and summer (Supplementary Figure 1A) when the most pronounced differences between surface and bottom layers were measured at the sampling stations in the Rižana estuary. Lower salinity in winter coincided with a higher flow rate of the Rižana (Supplementary Table 2). Taken together with the differences in water temperature between surface and bottom layers (up to 6°C), the water column was stratified in summer at all stations (Supplementary Figure 2).

Throughout the year, the water at all sampling stations was well oxygenated with the highest dissolved oxygen (DO) concentrations recorded in spring and summer (average value 8.2 and 7.8 mg L^–1^, respectively) and the lowest in autumn (average value 6.5 mg L^–1^) (Figure 2B). The mean DOC concentration was 102.7 ± 11.3 μmol L^–1^ with the highest values recorded in autumn at all stations (Figure 2C).

Concentrations of inorganic nutrients (ammonia, nitrate and nitrite, orthophosphate) and TDN were overall higher at both estuary stations in comparison to the marine stations (Figures 2D–F and Supplementary Figure 1B). The highest concentrations were measured at R-Estuary-1 station in winter, whereas no seasonal trend was observed at the other stations. Concentrations were higher at the surface compared to the bottom layer and coincided with lower salinity (Supplementary Figure 3).

Bacterial abundance in our data set showed significant seasonal variation (Kruskal–Wallis test, *p* < 0.0001). Higher bacterial abundance was observed in summer and autumn (Figure 2G) and correlated with higher seawater temperatures (Pearson correlation, *r* = 0.73, *p* < 0.0001, Supplementary Figure 3). On the other hand, bacterial carbon production (BCP), showed no significant seasonal variation yet differed significantly among stations (Kruskal–Wallis test, *p* < 0.001, Figure 2H). In all seasons, BCP was higher at estuary stations, reaching up to 51.1 μg C L^–1^ d^–1^ in summer, coinciding with low values of salinity (Pearson correlation; *r* = −0.67, *p* < 0.0001, Supplementary Figure 3) and high values of inorganic nutrients (Pearson correlation; *r* = from 0.43 to 0.76, *p* < 0.01, Supplementary Figure 3).

The taxonomic composition of the bacterial communities was investigated using Illumina 16S rRNA gene amplicon sequencing, which generated a data set of 40 samples with 3,341,665 sequences (Supplementary Table 4). Overall, we identified 7,561 bacterial ASVs with an observed richness from 212 to 1,338 bacterial ASVs per sample. Rarefaction curves did not reach a plateau in any of the examined communities; however, estimated asymptotic extrapolation to double the amount of sequences showed only few additional ASVs (Supplementary Figure 4). Thus, our sequencing depth represents most of the bacterioplankton diversity (Hsieh et al., 2016).

Alpha diversity of bacterial communities (Supplementary Figure 5) revealed significant seasonal differences in the Shannon diversity index (ANOVA test, F_3_,_31_ = 9.9, adjusted *p*-value < 0.0001) and evenness (ANOVA test, F_3_,_31_ = 10.7, adjusted *p*-value < 0.001). The Shannon diversity index and evenness significantly decreased from winter to spring and increased from summer to autumn (Tukey’s HSD *post hoc* test, adjusted *p*-value < 0.0001). Furthermore, alpha diversity indices revealed spatial differences in evenness (ANOVA test F_4_,_31_ = 7.3, adjusted *p*-value < 0.0001) with R-Estuary-1 and R-Estuary-2 having significantly different values from OS-Marine and SM-Outfall (Tukey’s HSD *post hoc* test, adjusted *p*-value < 0.05).

**FIGURE 3:**
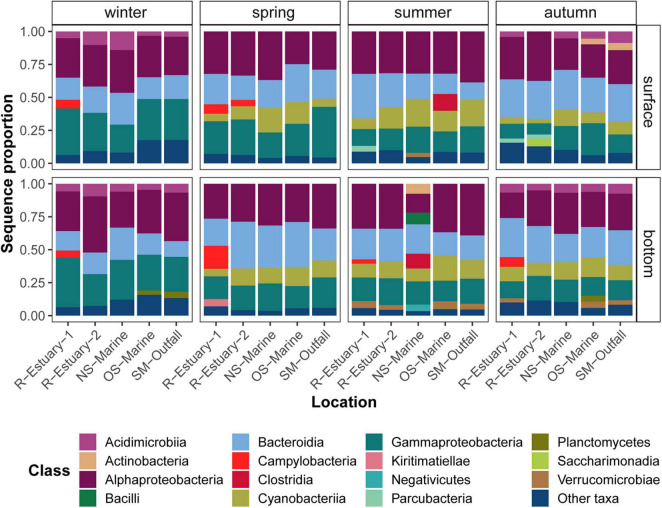
Sequence proportion of dominant bacterial classes at the surface and bottom layer in winter, spring, summer, and autumn during the 2018/2019 survey in the eastern part of the Gulf of Trieste (sampling stations: R-Estuary-1, R-Estuary-2. NS-Marine, OS-Marine and SM-Outfall). Classes showing a sequence proportion of < 3% were grouped together as “other taxa.”

Alphaproteobacteria, Bacteroidia, Gammaproteobacteria, and Cyanobacteria dominated the bacterial communities in all samples (Figure 3). We found that these groups formed a core microbiome, composed of 643 ASVs that were shared between all stations, representing more than 88% of the total reads (Supplementary Figure 12B). In all seasons, Alphaproteobacteria had the highest sequence proportions (mean sequence proportion 30 ± 5.3%), comprising mainly Rhodobacterales, that predominated in spring (17 ± 4% of the sequences), and of the SAR11 clade in winter (13 ± 7% of the sequences; Supplementary Figure 6A). Bacteroidia comprised 23 ± 6% of the sequences in the samples and consisted mainly of the Flavobacteriales (20 ± 5% of the sequences; Supplementary Figure 6B). Gammaproteobacteria comprised 21 ± 7% of the sequences per sample and consisted mainly of the Cellvibrionales, predominantly found in spring (11 ± 2% of sequences), whereas the SAR86 clade and the Thiomicrospirales mainly occurred in winter (11 ± 3% and 9 ± 4% of the sequences, respectively; Supplementary Figure 6C). Cyanobacteria were almost absent from winter bacterial communities (<1% of the sequences); however, on average, they represented 9 ± 6% of the sequences (Figure 3). Campylobacteria represented high sequence proportion at the estuarine stations (6 ± 5% and 1 ± 2% at stations R-Estuary-1 and R-Estuary-2, respectively) and were almost absent at the other stations (<0.1% of the sequences).

Bacterial communities showed both seasonal (PERMANOVA test, *R*^2^ = 0.378, *p* < 0.001) and spatial variation (by sampling stations PERMANOVA test, *R*^2^ = 0.102, *p* < 0.01; by sampling depth PERMANOVA test, *R*^2^ = 0.028, *p* < 0.05.; Supplementary Figure 7). Community composition was found to be mostly associated with natural seasonality-related parameters (ANOVA test, *p* < 0.001), such as DO, seawater temperature, and DOC (adj. *R*^2^ 12.2, 8.5, and 6.5, respectively). However, differences in community composition were also related to salinity and concentrations of inorganic phosphate (PO_4_^3–^; adj. *R*^2^ 3.1) and nitrogen (NH_4_^+^ and NO_2_^–^ + NO_3_^–^; adj. *R*^2^ 5 and 2.5, respectively) (ANOVA test, *p* < 0.05) (observed trends were shown with RDA analyses; Supplementary Figure 8 and Supplementary Text 2). These parameters may not only represent the natural influx of nutrients from river waters, but may also be associated with nutrient input from anthropogenic point and non-point pollution sources.

**FIGURE 4:**
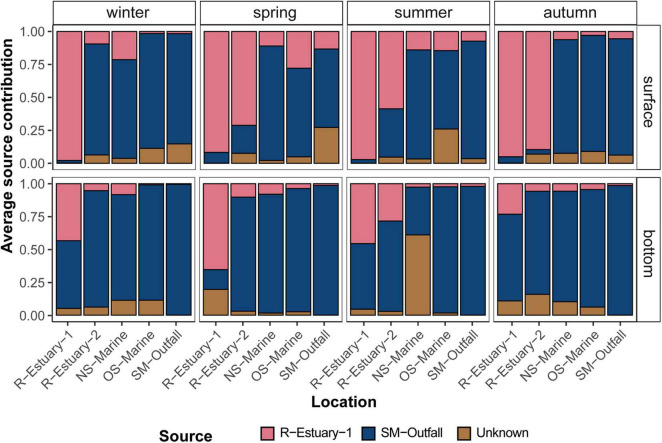
Proportion estimates of the estuarine (R-Estuary-1) and submarine (SM-Outfall) outfall bacterial communities at the surface and bottom layer in winter, spring, summer, and autumn in the 2018/2019 data set in the eastern part of the Gulf of Trieste (at sampling stations: R-Estuary-1, R-Estuary-2, NS-Marine, OS-Marine and SM-Outfall), using “SourceTracker.” Unknown—refers to the fraction of the communities’ source of which was not identified by the SourceTracker.

To test our hypothesis that bacterial communities were, to some extent, affected by wastewater pollution introduced *via* the estuary or *via* the submarine outfall, we applied the microbial source tracking (MST) Bayesian algorithm “SourceTracker.” This MST approach assumes that the diversity in a tested bacterial community (i.e., “sink” community) corresponds to the diversity of potential “source” communities (e.g., SM-outfall and R-Estuary-1) and allows identification of statistically probable links between them. To test the predictive accuracy of the source bacterial communities, we used the statistical learning validation approach “leave-one-out.” Each source sample was removed, in turn, from the training data set, and its origin was predicted based on the rest of the source samples in the data set. The assessed performance of the algorithm showed a strong signal of the distinct R-Estuary-1 bacterial communities. However, the algorithm was not able to clearly separate between the submarine outfall bacterial communities and the ambient seawater communities. Nonetheless, the MST analysis showed a strong effect of the estuary source community on the bacterial communities in the seawater throughout all seasons (Figure 4). The effect of the R-Estuary-1 source communities was higher at the surface compared with the bottom (2–90% vs. 1–65%, respectively). The effect decreased strongly with increasing distance from the discharge station at both depths (Figure 4).

**FIGURE 5:**
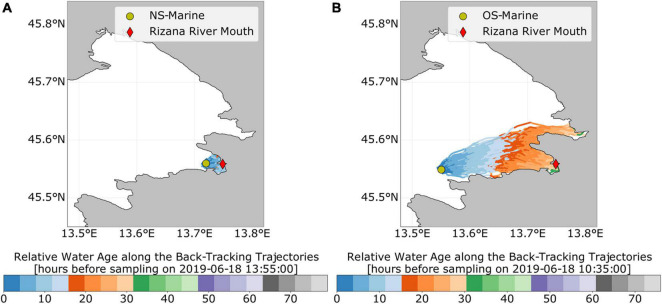
Lagrangian backtracking of water masses from two sampling stations during summer sampling in 2019. **(A)** NS-Marine station, **(B)** OS-Marine station. In each figure, the sampling station is marked by a green circle and the mouth of the Rižana river with the red diamond. The trajectories indicate from where the water masses may have arrived to the sampling location prior to the respective sampling time. Each trajectory is colored by relative water age prior to the sampling time.

To further validate the potential dispersion of wastewater-borne bacteria throughout the sampling area, as revealed by the MST, we applied a Lagrangian backtracking model. This approach allowed us to allocate the origin of surface and bottom water at all sampling stations 72 h prior to each sampling (Supplementary Material 3). These simulations suggest that the origin of the sampled surface water at the outermost sampling stations (NS-Marine and OS-marine) may have been the Rižana estuary at particular samplings, e.g., summer in our 2018/2019 data set (Figure 5). Estimates of relative water age at the sampling station can also be inferred from color along each trajectory: water masses, sampled at the NS-Marine station, took approximately 10–15 h to travel from the Rižana river mouth to the sampling station during our summer sampling in 2019 (Figure 5A). At the same time, water masses sampled at the OS-Marine station needed 20–30 h of advection from the river mouth to the sampling station (Figure 5B). Our model further indicates that, during our summer sampling in 2019, the surface water masses at the sampling station OS-Marine originate predominantly from the inner part of the Bay of Koper. Based on the backtracking simulations, an additional forward-tracking simulation was performed, in which 6,400 particles were released at the Rižana river mouth 24 h prior to the sampling. By the time of sampling (June 18, 2019, 10 a.m.), 80 of the 6,400 particles (1.25% of the initial particle density at the release site) reached the 1 km^2^ area around the OS-Marine sampling station. The simulated particle density at the sampling location peaked at about 300 particles km^–2^ (about 5% of the initial particle density at the release site). Additional simulations further suggest that the Soča estuary at the north of the Gulf of Trieste is also a potential source of bacterial indicators at the OS-Marine station (Supplementary Figure 13). However, this needs further clarification and is currently part of ongoing investigations.

### Bacterial Taxa as Pollution Indicators

**FIGURE 6:**
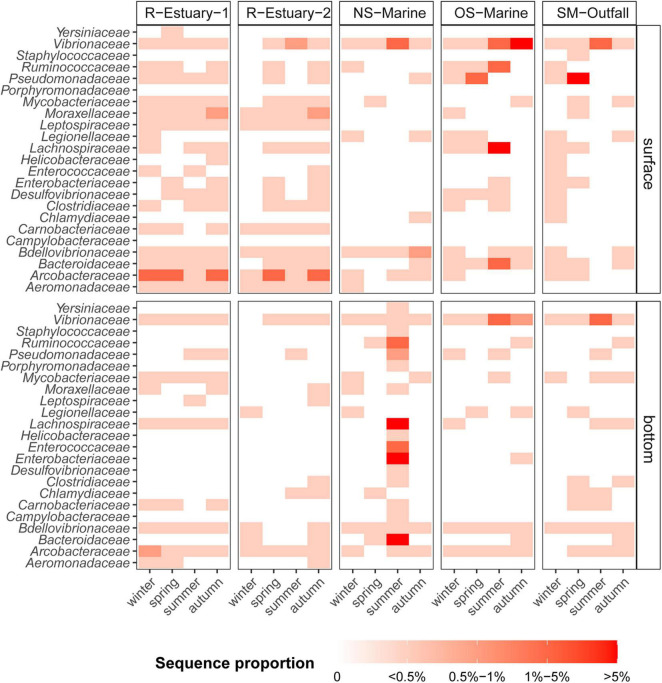
Sequence proportion heatmap (white = absent, red = high) of selected family-level bacterial indicators at five sampling stations (R-Estuary-1, R-Estuary-2, NS-Marine, OS-Marine and SM-Outfall) in winter, spring, summer, and autumn sampling during the 2018/2019 survey.

The traditional cultivation techniques we applied revealed the presence of total coliform bacteria on the surface at all sampling stations (except at station OS-Marine), which suggested potential fecal and sewage pollution (Supplementary Table 5). Coliform bacteria were observed at the estuarine stations throughout the year with the highest numbers at the surface in autumn and winter. To further investigate the presence of wastewater pollution–associated bacteria within the water column, we screened for traditional and alternative bacterial indicator taxa (Supplementary Table 1) in our 16S rRNA amplicon sequences data set. We found that 623 ASVs in our data set were assigned to bacterial indicator taxa that represented from 0.1 to 34% of the sequence proportion for a specific sample. Bacterial indicators were present throughout the whole year at all sampling locations and at both sampling depths. In the river estuary (R-Estuary-1 and R-Estuary-2), bacterial indicators were persistent throughout the year and comprised up to 5% of the communities (17–69 ASVs). The most abundant (in terms of sequence proportion) bacterial indicator in the estuary was the family *Arcobacteraceae* that comprised up to 4% of the community, followed by *Moraxellaceae* (up to 0.6%), *Vibrionaceae* (up to 0.6%), and *Mycobacteriaceae* (up to 0.5%) (Figure 6). During our 2015/2016 preliminary sampling (see section “Materials and Methods”), we observed a decrease in the relative sequence abundance of bacterial indicators with distance from the river outfall (Supplementary Figure 10). In the 2015/2016 data set, 949 ASVs were assigned to bacterial indicators, and they represented from 0.2 to 52% of the sequence proportion for a specific sample. In 2015/2016, the highest sequence proportion of bacterial indicators was observed at the sampling station in the river mouth near the WW outfall (R-Outfall) with the overall relative sequence proportion of bacterial indicators ranging from 25 to 52%. The highest contribution was from the *Arcobacteraceae* family, reaching a sequence proportion of 51% (R-Outfall). Bacterial indicators represented up to 8% of the community at the submarine outfall station (SM-Outfall1) in our 2018/2019 data set and were associated with *Pseudomonadaceae* (up to 7%) and *Vibrionaceae* (up to 2%) (Figure 6).

The highest sequence proportion of bacterial indicators in our 2018/2019 data set was observed in the summer communities at the NS-Marine station, reaching up to 34% of the sequences (216 ASVs) in the bottom layer. Bacterial indicators in this particular community were associated with both traditional indicators *Enterobacteriaceae* and *Enterococcaceae* (9 and 2% of sequences, respectively) as well as alternative indicators such as *Bacteroidaceae* (10%), *Lachnospiraceae* (8%), and *Ruminococcaceae* (1%) (Figure 6). In summer, the sequence proportion of bacterial indicators was high also in the surface sample at the OS-Marine station, reaching up to 13% of the sequences (111 ASVs), mainly represented by *Lachnospiraceae* (6%), *Ruminococcaceae* (4%), *Bacteroidaceae* (1%), and *Vibrionaceae* (1%) (Figure 6).

**FIGURE 7:**
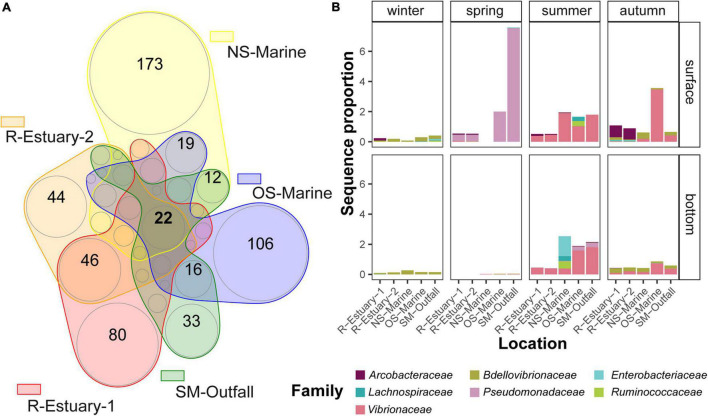
Visualization of bacterial community relationships between different stations based on the presence/absence of ASVs belonging to microbial indicators in the 2018/2019 data set using a Venn diagram **(A)**. The insert circles are approximately proportional to the number of ASVs in each region. The numbers of shared ASVs between stations that are lower than 10 are not shown. Graph **(B)** represents the sequence proportion of bacterial indicators that were shared among all stations.

Determination of the similarity of microbial communities at different stations was performed based on analysis of presence/absence of ASV (Supplementary Figure 12A) for the 2018/2019 data set. Stations R-Estuary-1 and R-Estuary-2 had the highest number of unique ASVs (1,626 and 1,295, respectively), but these unique ASVs represented only a small proportion of the total reads (0.7 and 0.4%, respectively). Within the core microbiome, we found in total 22 ASVs assigned to bacterial indicators (Figure 7A), belonging to the families *Enterobacteriaceae, Pseudomonadaceae, Vibrionaceae*, *Bdelovibrionaceae, Lachnospiraceae, Ruminococcaceae*, and *Arcobacteraceae* (Figure 7B).

### Characterization of Arcobacter Diversity

**FIGURE 8:**
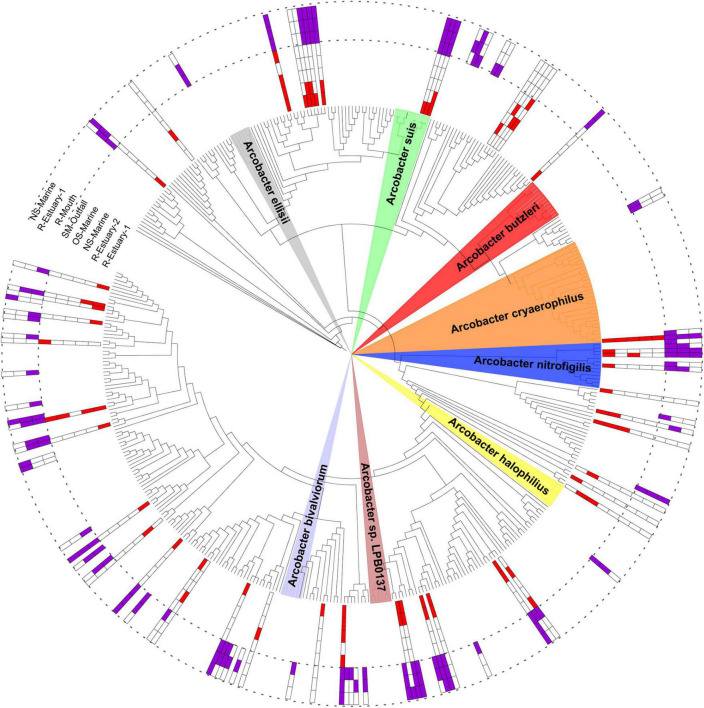
Phylogenetic tree with ASVs from the 2015/2016 and 2018/2019 data sets assigned to the *Arcobacteraceae* family and its neighbors. Colored areas are associated with the taxonomic classification of known *Arcobacter* species. Magenta squares indicate the presence of ASV at the sampling station in the 2015/2016 data set; red squares indicate the presence of ASV in the 2018/2019 data set.

The *Arcobacteraceae* family was further investigated as a group with potential pathogen species. A total of 48 ASVs belonging to the *Arcobacteraceae* family were present in the 2018/2019 data set, whereas 93 ASVs were detected in the 2015/2016 sampling (Supplementary Material 2). To explore *Arcobacter* diversity, a phylogenetic tree was constructed including ASVs from both 2015/2016 and 2018/2019 data sets (Figure 8). The majority of detected ASVs were clustered together with reference *Arcobacter* sequences classified as uncultured bacterium, uncultured *Epsilonproteobacteria bacterium*, or uncultured *Arcobacter* sp. One ASV was clustered close to known pathogenic species *Arcobacter cryaerophilus* and was present at all sampling stations with a mean relative sequence abundance of up to 0.14% (at R-Estuary-1) in the 2018/2019 data set and up to 5.75% (at R-Outfall) in the 2015/2016 data set (Supplementary Figure 11). Furthermore, one additional ASV related to *A. cryaerophilus* was recorded in the 2015/2016 data set (mean relative abundance at R-Outfall 0.22%). Other ASVs clustered with known *Arcobacter* species were associated with *Arcobacter nitrofigilis* and *Arcobacter suis* (Supplementary Table 7).

## Discussion

Marine microbial communities can be sensitive to perturbations in their surroundings, such as nutrient fluctuations and the introduction of allochthonous microbial populations (Nogales et al., 2011; Saarenheimo et al., 2017). Despite improvements in wastewater quality discharged from WWTP, the regular detection of wastewater-borne pathogens in the marine environments suggests that fecal pollution remains a problem (Newton et al., 2013; Luna et al., 2016; Carney et al., 2019), especially in coastal areas subjected to diverse point and non-point pollution sources. The presence of bacterial indicators in coastal environments continues to be monitored using traditional culture-based approaches that have been questioned lately because of their limitation to detect only certain bacterial indicators (Stewart et al., 2008; Converse et al., 2011; Bradshaw et al., 2016; Caruso et al., 2016). Furthermore, our understanding of the spatio-seasonal dynamics of potentially pathogenic bacteria in marine ecosystems is limited and knowledge on environmental factors that affect their population dynamics is lacking. To maintain healthy marine ecosystems and their services, it is crucial to gain an insight into the distribution of bacterial indicators and niches they occupy.

We analyzed the pelagic microbiome of the eastern part of the Gulf of Trieste (northern Adriatic), which has been proposed as a model area for the study of the importance of wastewater and land-born nutrient inputs in a subtropical coastal ecosystem (Cozzi et al., 2012). We focused on the effects of wastewater pollution on the structuring of the coastal microbiome. The sampling design of this study enables us to perform comparative analyses of the microbial community at spatial and seasonal dimensions, from which we were able to deduce patterns of variation driven by distance from the wastewater pollution sources (submarine outfall and estuary) and seasonal changes in the area.

### Bacterial Indicators Are Part of the Core Coastal Microbiome

The core microbiome of our model system comprises Alphaproteobacteria, Bacteroidia, Gammaproteobacteria, and Cyanobacteria as previously reported for coastal marine ecosystems (Lindh et al., 2015; Maresca et al., 2018; Pearman et al., 2018; Ruiz-González et al., 2019; Adyasari et al., 2020) and also the Northern Adriatic (Celussi and Cataletto, 2007; Šilović et al., 2012; Tinta et al., 2015; Paliaga et al., 2017; Buccheri et al., 2019). Our model ecosystem is strongly subjected to seasonal variations in seawater temperature, solar radiation, weather patterns (winds, freshwater inputs), changes in nutrient availability, stratification, etc., which were recognized as important forcing factors driving seasonal variations in bacterial community structure and function (Gilbert et al., 2012; Fuhrman et al., 2015; Auladell et al., 2021). Therefore, we conducted *in situ* surveys in different seasons to be able to distinguish between variations of microbial communities triggered by seasonal variations of abiotic and biotic conditions and those caused by different distance from pollution sources. Our observations showed that the bacterial community exhibited significant seasonal variation in abundance with the highest abundance determined in the warmer part of the year (Figure 2). We observed higher diversity and evenness in winter, which is in accordance with previous studies of pelagic coastal ecosystems (Gilbert et al., 2010; García-Armisen et al., 2014). Bacterial community composition showed pronounced differences at seasonal scale, mainly driven by seasonal changes in temperature, dissolved oxygen, inorganic nutrients, DOC concentration, and to some extent salinity as previously reported for this area (Celussi et al., 2009; Šilović et al., 2012; Tinta et al., 2015). Sampling location and depth had a lower structuring impact on bacterial community composition in our study. This is consistent with a previous study from the nearby Venice Lagoon, where temporal variation in seawater bacterioplankton composition was higher compared with spatial variation (Celussi et al., 2009). Next, we used 16S rRNA gene amplicon sequencing data to investigate the presence of traditional fecal indicator taxa (i.e., taxa determined using culture-based methods) and alternative indicators (i.e., taxa determined using molecular-based approaches) of wastewater pollution as has been successfully applied previously in other areas (Maugeri et al., 2004; Newton et al., 2013; Luna et al., 2016; Buccheri et al., 2019). Bacterial indicators accounted cumulatively for up to 34% of all sequences per sample in our 2018/2019 sampling campaign and even up to 52% of sequences in our 2015/2016 data set. Taxa belonging to the bacterial indicators of wastewater pollution *Enterobacteriaceae*, *Lachnospiraceae*, *Ruminococcaceae, Arcobacteraceae, Pseudomonadaceae*, *Vibrionaceae*, and *Bdelovibrionaceae* were part of the core pelagic microbiome, i.e., were detected at all sampling stations (Figure 7). On the other hand, cultivation-based techniques we applied did not detect total coliforms at our reference marine station (OS-Marine). Thus, our study confirms that high-throughput sequencing can increase the resolution of monitoring for the presence of rare taxa that can serve as indicators of environmental change (McLellan and Eren, 2014) and allow the description of overall fluctuations in community structure to assess environmental status from a holistic point of view (Gilbert et al., 2010; Campbell et al., 2015; Caruso et al., 2016). Among the alternative indicators detected in our core microbiome, *Lachnospiraceae* (representing up to 8% of sequence proportion in samples) have previously been suggested as an important source-specific indicator because they are strongly related to untreated human sewage but, in contrast, are low or absent in animal feces (McLellan and Eren, 2014; Feng et al., 2018). Besides this, the presence of *Arcobacteraceae* (comprising up to 4 and 51% of the microbial community in 2018/2019 and 2015/2016 data sets, respectively) has been frequently reported in anthropogenically impacted coastal areas in the Mediterranean Sea (Maugeri et al., 2004; Fera et al., 2010; Buccheri et al., 2019), and sewage has been recognized as their potential source (Collado and Figueras, 2011; Fisher et al., 2014). Although members of some indicator families found in the core microbiome of our study area, such as *Pseudomonadaceae, Vibrionaceae*, and *Bdelovibrionaceae*, could be natural inhabitants of the marine environment, they have been previously connected with wastewater pollution and deserve attention because they contain human or animal pathogens (Cavallo and Stabili, 2004; Cañigral et al., 2010; Luczkiewicz et al., 2015) or have an impact on ecology as obligate predators of other bacteria (Jurkevitch, 2020).

Our results reveal that bacterial indicators of wastewater pollution were not restricted to stations at the vicinity of the wastewater pollution source, but were widely distributed in this coastal ecosystem (Figures 6, 7). In contrast, Borin et al. (2009) describe significant spatial differences in microbial community composition in the sediment of the Venice Lagoon with a higher presence of *Vibrionaceae* at stations adjacent to urban areas. This could be explained by the fact that, although water masses are moving and mixing, sediments are site-specific and, therefore, likely accumulate a stronger signal over time. Our observation that the magnitude of indicator signature did not decrease with increasing distance from pollution sources (i.e., estuary and submarine outfall) suggests that the entire area is under anthropogenic pressure and receives loads of allochthonous microbes, potentially *via* numerous sources. This coastal zone is indeed highly populated, and in addition to WWTPs, effluents entering the sea, pressures from an industrial port, aquaculture facilities, and agricultural runoff cannot be neglected. Unfortunately, the contribution from non-point sources, such as overland runoff, is more difficult to characterize. However, several studies report increased fecal contamination in urban rivers or coastal marine waters in connection with rainfall events (Converse et al., 2011; Garner et al., 2017; Rio et al., 2017; Baral et al., 2018). Stormwater runoff can contribute to loads of fecal pollution originating from various sources, including humans, livestock, and wildlife, and is closely dependent on the land use in watersheds (e.g., agricultural and urban areas) (Converse et al., 2011). We observed a higher presence of bacterial indicators at the surface in our study area in contrast to studies that reported a higher presence of fecal pollution indicators at bottom layers and emphasized a source *via* sediment resuspension (Perkins et al., 2014; Bradshaw et al., 2016; Hassard et al., 2016; Paliaga et al., 2017). Although we did not observe clear seasonal patterns of bacterial indicators, some ASVs within the family *Vibrionaceae* were positively correlated with seawater temperature and dissolved oxygen concentration (Supplementary Figure 9). A similar trend was previously described for the abundance of fecal indicator bacteria in 12 ports in the northern Adriatic Sea, including the port of Koper, located in our study area, where no significant seasonal variability of bacterial indicators in seawater was observed (Luna et al., 2018).

### Estuary as a Potential Source of Bacterial Indicators to the Coastal Ecosystem

We detected elevated bacterial abundance in the estuary at the vicinity of wastewater outfall in summer and autumn sampling (Figure 2). Furthermore, using traditional culturing techniques, we determined an elevated number of total coliform bacteria in the estuarine surface samples, indicating the introduction of allochthonous bacteria, in agreement with previous studies (Turk et al., 2007; Gonçalves et al., 2018). On the other hand, alpha diversity analyses showed lower evenness in samples in the vicinity of wastewater outfall, indicating that the relative sequence abundance of species was not evenly distributed, suggesting higher perturbations of bacterial communities. The alpha diversity metric has been previously suggested as a useful tool for assessing anthropogenic impacts on coastal ecosystems and other water bodies (Cordier et al., 2020; Ferrera et al., 2020). Studies of freshwater (rivers and lakes) ecosystems report that the vicinity of wastewater sources favors certain microbial groups, leading to biotic homogenization (Drury et al., 2013; Lu and Lu, 2014; Saarenheimo et al., 2017), whereas a contrasting pattern is reported for polluted marine sediments, with which disturbance promoted co-occurrence of phylogenetically distinct species, resulting in increased diversity (Galand et al., 2016).

In addition to wastewater discharge, the studied estuary is subject to a variety of human influences, including port activities and runoff that drains urban/rural and agricultural regions at river watersheds. Differences in water characteristics were evident in higher concentrations of organic and inorganic nutrients (TDN, NH_4_^+^, NO_2_^–^ + NO_3_^–^, PO_4_^3–^) at stations adjacent to the estuary compared to offshore marine sites (Figure 1). This result is in accordance with previous observations, showing the important influence of river outflow along with receiving wastewater on nutrient conditions in this coastal area (Turk et al., 2007; Cozzi et al., 2012). Estuarine stations were also characterized with higher BCP in comparison to offshore marine stations (especially in the surface layer, in accordance with Tinta et al., 2015), which could be triggered by nutrient enrichment (Rowe et al., 2018). On the other hand, no clear signal in terms of abundance, production, diversity indices, or nutrient conditions was observed near the submarine outflow. This was expected as the wastewater entering the sea through submarine pipelines with numerous diffusers could be immediately diluted (Malačič and Mozetič, 2005), whereas the estuarine circulation could further disperse the polluted water in a horizontal direction (Soczka Mandac et al., 2014; Álvarez-Vázquez et al., 2016).

Our MST analysis revealed that the estuary could be an important source of wastewater-related microorganisms in our study area, such as *Arcobacteraceae*, *Moraxellacea*, *Vibrionaceae*, and *Aeromonadaceae* (Supplementary Table 6). The influence of an estuarine-associated community was determined at all sampled stations in the middle of the bay and was observed also at the offshore marine station, located more than 15 km from the estuary. Furthermore, the effect of the estuary community was higher at the surface in comparison with the bottom layer. As previously reported for this area, water circulation is strongly dependent on a number of processes, including tides, wind, waves, and river discharge (Malačič et al., 2006, 2012; Cosoli et al., 2013). Our backtracking Lagrangian simulations indicated that, during summer sampling, surface water masses at the offshore sampling station (OS-Marine) originated predominantly from the Bay of Koper and took a relatively short time of 20–30 h to drift from the source (Rižana estuary) to the offshore sampling station (Figure 5). This means that river discharge in combination with local surface winds and ocean currents importantly influences the presence of bacterial indicators at stations relatively far from pollution sources. At the same time, the highest sequence proportions of bacterial indicators were observed in summer at offshore and nearshore marine stations (NS-Marine) in comparison with other sampling periods (Supplementary Figure 10). Additional forward-tracking Lagrangian simulations allowed an estimate that less than about 5% of the initially released particles from the estuarine stations reached the OS-Marine sampling station within 20–30 h of drift. Obviously, physiochemical parameters, such as temperature, ultraviolet radiation, salinity, oxygen, and nutrient concentrations, may also affect the survival, activity, and propagation of fecal microorganisms (Pommepuy et al., 2005; Korajkic et al., 2015) including potential pathogens [e.g., *Vibrio* (Montánchez et al., 2019)]. Previous studies in freshwater systems suggest that seasonal patterns may influence the resilience of systems because WWTP-induced communities were found to trigger changes in the microbial community that are followed by a rapid return to the original state only in certain seasons, whereas the effect persists in other seasons (García-Armisen et al., 2014). Although our results did not show seasonal trends in the presence of bacterial pollution indicators, additional studies would be necessary to explore other environmental factors that influence their survival and activity and to better understand the risk they represent under different environmental conditions.

In a contrasting scenario, these bacterial indicators could attach to different particles and, hence, settle down at the seafloor more rapidly (Hassard et al., 2016). It has already been emphasized that sediments can act as reservoir of pathogens that can be resuspended under certain conditions (Korlević et al., 2015; Quero et al., 2015; Chen et al., 2019). This could also be the case in our study, in which we observed elevated relative sequence abundance of bacterial indicators in the bottom layer during summer at the nearshore marine station, located 2.5 km from a river pollution source. However, further studies are needed to better understand the presence of bacterial indicator taxa in sediments and the rate of resuspension into the water column under different conditions. MST computational approaches are tools developed to characterize the sources of microbial contamination. They can be applied on different markers and indicators commonly monitored in water samples as in the case of the Ichnaea^®^ tool (Sanchez et al., 2011; Ballesté et al., 2020) or on total 16S rRNA amplicon data as in the case of SourceTracker (Knights et al., 2011). The SourceTracker tool has been already applied in different environmental studies (Neave et al., 2014; Henry et al., 2016; Saarenheimo et al., 2017; Brown et al., 2019) to predict the extent to which microbial communities at an environmental site can be attributed to fecal sources. However, whereas MST methods can only identify sources at the time and site of sampling, oceanographic modeling and pollution source prediction could add useful information during non-sampled events, providing especially valuable information for water management authorities. Further efforts are underway to employ atmospheric, oceanographic, and Lagrangian models in real time prior to sampling.

### Arcobacteraceae as Potential Pathogens

Among potentially pathogenic bacteria, *Arcobacteraceae* were present in all estuarine samples (at all seasons at the surface and in the bottom layer) and represented up to 3.7% of sequences. Even higher relative sequence abundances of *Arcobacteraceae* were detected during our 2015/2016 sampling campaign when this family represented up to 36% of the sequences at the station in the river mouth in the immediate vicinity of the WWTP outfall. At the same time, we also measured higher nutrient concentrations (Supplementary Table 3). The genus *Arcobacter* comprises 29 recognized species (Isidro et al., 2020; On et al., 2020) of which *A. butzleri*, *A. cryaerophilus*, *A. skirrowii*, and *A. thereius* are considered to be human and animal pathogens (Pérez-Cataluña et al., 2018). *Arcobacter* usually occur at low abundances in natural marine and freshwater environments (less than 1%) (Carney et al., 2019), but their relative abundance can be much higher in environments with high levels of fecal pollution (Collado et al., 2008, 2010; Fisher et al., 2014). As reported, treated wastewater could be an important source as high relative abundance of *Arcobacteria* (reaching up to 30%) has been found in the WWTPs effluent despite advanced biological and chemical treatments that meet European standards (Kristensen et al., 2020). Furthermore, using a fluorescence *in situ* hybridization and metagenomics approach, the same study reported *A. cryaerophilus* to be the most abundant *Arcobacter* species in the influent and treated wastewater effluent (Kristensen et al., 2020).

The presence of *Arcobactereace* has been reported in previous studies in the Mediterranean Sea. Buccheri et al. (2019) report a relative sequence abundance of the genus *Arcobacter* of up to 36.7% in the marine samples in the immediate vicinity of wastewater output in a Mediterranean tourist site (Catania, Italy), whereas its relative abundance in the sample 400 m away was much lower (0.22%). In our study, we did not notice a seasonal pattern in the overall *Arcobacter* sequence abundance, which is in contrast to previous findings showing that pathogenic *Arcobacters* might survive better at lower temperatures (D’Sa and Harrison, 2005) and are observed more frequently in winter and spring (Maugeri et al., 2004; Fera et al., 2010). Other environmental factors (such as freshwater intrusion) might have a stronger influence on the presence and relative sequence proportion of *Arcobacter* in our study area. High levels of *Arcobacter* have previously been linked with periods of significant rainfall as reported for Lake Erie (Lee et al., 2012) and Lake Michigan (Newton et al., 2013). Carney et al. (2019) report large and rapid increases in the relative abundance of the genus *Arcobacter* from < 1% to up to 70% of sequences after stormwater intrusion on the coast of Sydney (Australia).

Pathogenic species of *Arcobacter* (e.g., *A. butzleri, A. cryaerophilus, A. skirrowii*) have been previously isolated from higher organisms, e.g., bivalve mollusks (Levican et al., 2012; Leoni et al., 2017) and fish (Rathlavath et al., 2017), indicating that higher marine organisms could act as reservoirs of pathogenic species in the marine environment and as vectors for transmission to more distant areas. Note that the mariculture facilities located in our study area could represent a potential hot spot for these pathogens. However, this was beyond the research focus of our *in situ* surveys, but will be addressed in the future. A recent study confirmed the occurrence of multidrug resistance strains of *A. butzleri* and *A. cryaerophilus* in seaweeds and seawater samples (Sciortino et al., 2021). The isolation of *Arcobacter* from different sources suggests concerns regarding its importance as food- and water-borne human pathogens (Ottaviani et al., 2013; Ramees et al., 2017).

The 16S rRNA sequencing technique is widely used to describe bacterial community composition, but has low discriminatory power at the species level for some genera (Janda and Abbott, 2007). Even though we were not able to resolve the species identity of *Arcobacter* ASVs in our data set, we found high diversity within this group with 48 ASVs belonging to the *Arcobacteraceae* family in our 2018/2019 data set and 93 ASVs in our 2015/2016 data set. With the phylogenetic analyses, we revealed that some sequences in our data set were affiliated with known pathogenic taxa (Figure 8). One ASV (ASV55), detected at all stations, was clustered closely with *A. cryaerophilus*, which is classified as a “serious hazard to human health” according to the International Commission on Microbiological Specification for Foods (Tompkin et al., 2002). *A. cryaerophilus* has been associated with diarrhea and bacteremia in humans and with abortion, mastitis, and diarrhea in animals (Ho et al., 2006). This ASV was found at all sampling stations with the highest sequence proportion in summer 2015 (sequence proportion 13%).

### Conclusion and Future Directions

We found that bacterial indicators of wastewater pollution were widespread in our model coastal ecosystem throughout the year, suggesting that the area is under the influence of different point and non-point pollution sources. The overall coastal microbiome showed more pronounced seasonal than spatial dynamics, but these seasonal variations were not observed for bacterial indicators. Our source tracking analysis, supported with Lagrangian tracking models, revealed the importance of river water as a vector for transmission of allochthonous microbes into marine systems. We want to highlight the power of source tracking of bacterial populations, which can provide clues to relevant sampling areas, and on the other hand, allow the retroactive use of the obtained sampling results to validate surface ocean dynamics in the numerical models. Our results confirm that molecular analyses supported with statistical and oceanographic models give more profound insights into the environmental state and can help to assess the potential pollution sources and health risk they represent. However, our *in situ* survey was performed only on a limited sampling area; thus, to better understand the potential scale of wastewater-derived pollution dispersion, an effort should be made to upscale sampling areas in the future. Furthermore, future studies should continue to develop and test standardized culturing-independent methods, including amplicon sequencing or more rapid molecular assays, such as quantitative-PCR (Ghaju Shrestha et al., 2019). Implementation of rapid molecular analyses in combination with the use of oceanographic and statistical models can help improve environmental monitoring by increasing the frequency of monitoring and expanding the range of indicators and can be used to predict the spread of pollutants and associated health risks over larger areas.

## Data Availability Statement

The original contributions presented in the study are publicly available. This data can be found here: https://www.ebi.ac.uk/ena/browser/view/PRJEB46868.

## Author Contributions

NO designed and conducted the sampling, acquired the data, performed the data analysis, and drafted and submitted the final version of the manuscript. EF performed the data analyses and drafted and revised the manuscript. KK performed the chemical analysis and drafted the manuscript. ML conducted the Lagrangian tracking models and drafted the manuscript. TT and VT designed the sampling and drafted and revised several versions of the manuscript. All authors contributed to the article and approved the submitted version.

## Conflict of Interest

The authors declare that the research was conducted in the absence of any commercial or financial relationships that could be construed as a potential conflict of interest.

## Publisher’s Note

All claims expressed in this article are solely those of the authors and do not necessarily represent those of their affiliated organizations, or those of the publisher, the editors and the reviewers. Any product that may be evaluated in this article, or claim that may be made by its manufacturer, is not guaranteed or endorsed by the publisher.

## Footnotes

1
https://www.arb-silva.de/aligner/
