# Supplementary material for: Bacterial Indicators Are Ubiquitous Members of Pelagic Microbiome in Anthropogenically Impacted Coastal Ecosystem
Source: Front Microbiol. 2022 Jan 17;12:765091. doi: 10.3389/fmicb.2021.765091 (PMC8801744; doi:10.3389/fmicb.2021.765091)
Supplement: Supplementary file 1 [file Data_Sheet_1.docx]

Supplementary Material

# Supplementary Text 1

## *Material and methods: Oceanographic and Atmospheric model setup*

A chain of nested general circulation ocean models (based on NEMO3.6 engine) was employed to downscale circulation from high-resolution (1^o^/111) full Adriatic basin model through an intermediate Northern Adriatic model (1^o^/333) to final extremely high horizontal resolution (1^o^/648 or roughly 120 m) model covering the Gulf of Trieste. Full Adriatic basin model is presented in detail in (Ličer et al., 2020). Nesting was implemented uni-directionally from parent to child grid at all nesting levels, with Flather boundary conditions for barotropic variables and Orlanski boundary conditions for baroclinic variables and tracers. Bottom friction was set to be nonlinear and employed a logarithmic boundary layer. Operators for lateral diffusion of momentum and tracers were Laplacian acting over geopotential surfaces. Vertical diffusion was parameterized using a generic length scale (GLS) turbulence scheme. Wave breaking contribution to surface mixing was parameterized following Craig and Banner formulation (Craig and Banner, 1994). Vertical discretization of the Gulf of Trieste model consists of 21 partial *z* steps.

Atmospheric conditions over the Gulf of Trieste around sampling dates were simulated using an operational using operational version of ALADIN SI atmospheric model at the Slovenian Weather Service. ALADIN SI runs on a 432×432 horizontal Lambert conic conformal grid, centered on Slovenia, with 4.4 km resolution and 87 vertical levels with the model top at 1 hPa atmospheric pressure. Further details about the atmospheric model setup are available in Strajnar et al. (2015, 2019) and (Ličer et al., 2016).

To initialize the Lagrangian back-tracking simulations, a total of 1280 virtual passive drifters were released within ocean model simulation at the surface of the water column at each sampling station at each sampling time. The initial spatial distribution of the virtual drifters was a radially symmetric Gaussian with a 10 m standard deviation around the sampling station. Passive drifter velocities are determined in the OceanDrift module as a linear combination of ocean currents and 2 percent of the surface wind velocities (Dagestad et al., 2018). To account for subgrid turbulent diffusion, this deterministic combination of current and wind velocities is further perturbed during each computation step by random velocity fluctuations, which are sampled from a Gaussian velocity distribution with a 0.1 m s^-1^ standard deviation and zero mean. A 2^nd^ order Runge-Kutta method was employed to integrate these passive drifter velocities in half-hourly steps backwards in time (for 72 hours), thus obtaining their respective locations at various time steps prior to each sampling.

# Supplementary Text 2

***Results: RDA***

We explore whether the presence of occurring ASVs in the overall coastal pelagic microbiome was associated with the measured environmental parameters (temperature, salinity, dissolved oxygen, DOC, TDN, NO_2_^-^+NO_3_^-^, NH_4_^+^, PO_4_^3+^, C:N). Because of considerable collinearities between the measured environmental variables (especially organic and inorganic nutrients), the variable selection was applied. A set of 7 environmental parameters was selected with *ordistep* function and further used for the RDA analyses of bacterial community data (**Supplementary Table 5**). Dissolved oxygen, seawater temperature, PO_4_^3+^, DOC and NO_2_^-^+ NO_3_^-^ significantly explained differences in the community composition (ANOVA test, p < 0.001), while salinity and NH_4_^+^ explained differences at a lower significance level (ANOVA test, p < 0.05). The cumulative variance explained by the selected set of environmental parameters was 47.9 %. Variance partitioning showed that the highest individual contributions were attributed to dissolved oxygen, sea temperature, DOC and PO_4_^3+^ (R adj. square 12.2, 8.5, 6.5 and 3.1 %, respectively). Four axes were found to be significant (RDA 1, RDA 2, RDA 3 and RDA 4) (ANOVA test, p < 0.001), among that RDA axis 1 and RDA axis 2 (**Supplementary Figure 8**) explained 17.5 % and 12.6 % of the total variation, respectively. RDA ordination showed a strong separation of bacterial communities, corresponding to different sampling seasons (**Supplementary Figure 8, A)**. ASVs belonging to dominant bacterial classes were widely distributed in the RDA plot, suggesting that selected environmental parameters differently affect ASVs within these classes (**Supplementary Figure 8, B-E**). On the other hand, ASVs belonging to the class Campylobacteria were aggregated in the center of the RDA plot (**Supplementary Figure 8, F**), suggesting that they were less affected by measured environmental parameters, which influence ordination on the RDA plot.

# Supplementary Text 3

***Results: RDA***

RDA ordination shows aggregation of ASVs belonging to bacterial indicators in the center of the RDA plot. Both traditional (*Enterobacteriaceae*) and alternative indicators (*Bacteroidaceae, Lachnospiraceae* and *Arcobacteraceae*) behaved similarly and did not show correlation with tested environmental parameters (**Supplementary Figure 9**). Different ordination was observed for the *Vibrionaceae* family, where some ASVs were positively correlated with sea temperature and dissolved oxygen concentration (**Supplementary Figure 9, F**).


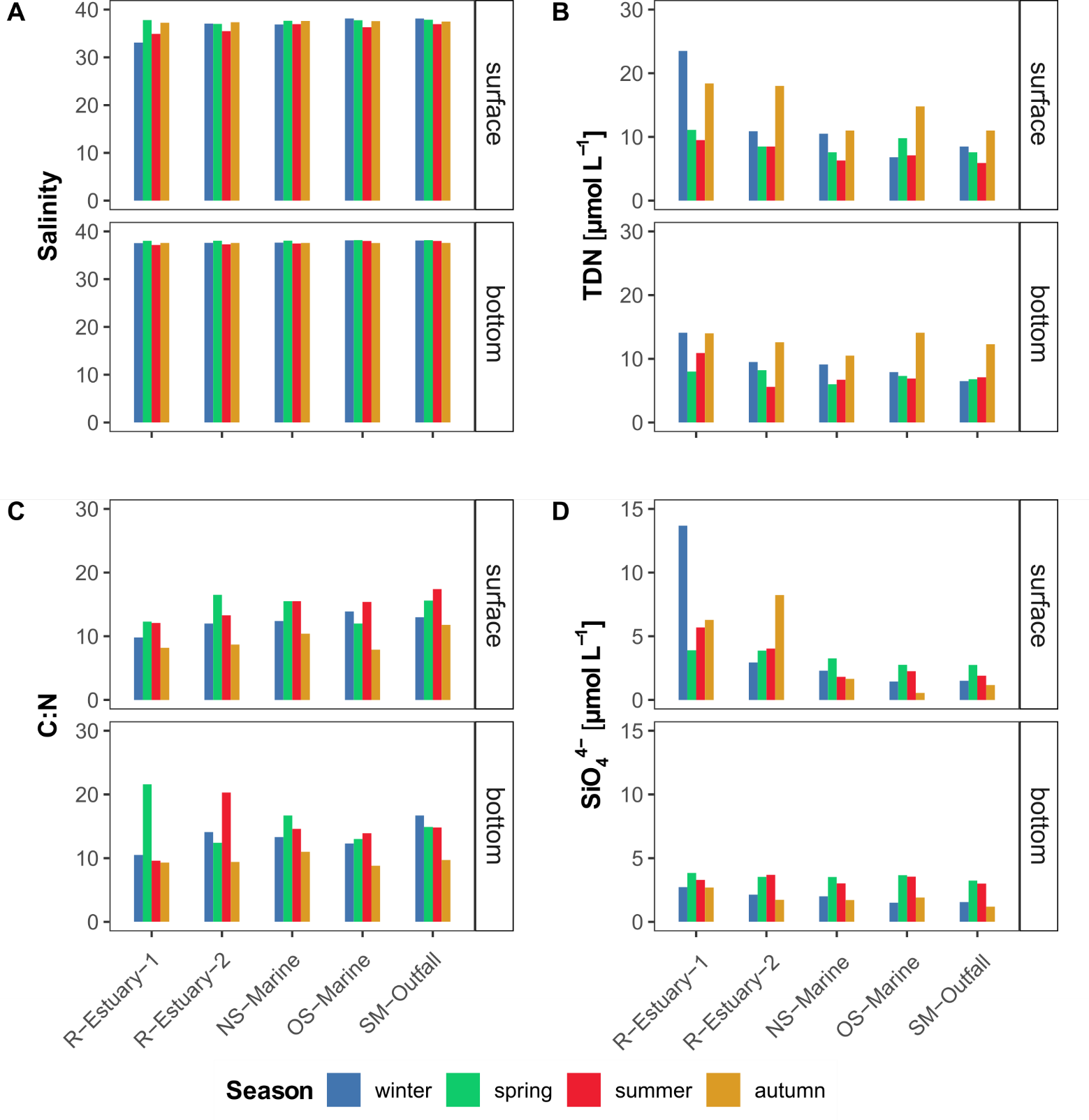


**Supplementary Figure 1:** Dynamics of selected environmental parameters at the surface and in the bottom layer in the winter, spring, summer and autumn during the 2018/2019 survey in the Gulf of Trieste at sampling stations: R-Estuary-1, R-Estuary-2, NS-Marine, OS-Marine and SM-Outfall. Salinity **(A)**, total dissolved nitrogen **(B)**, carbon to nitrogen ratio (molar) **(C)** and silicate **(D)**.

**
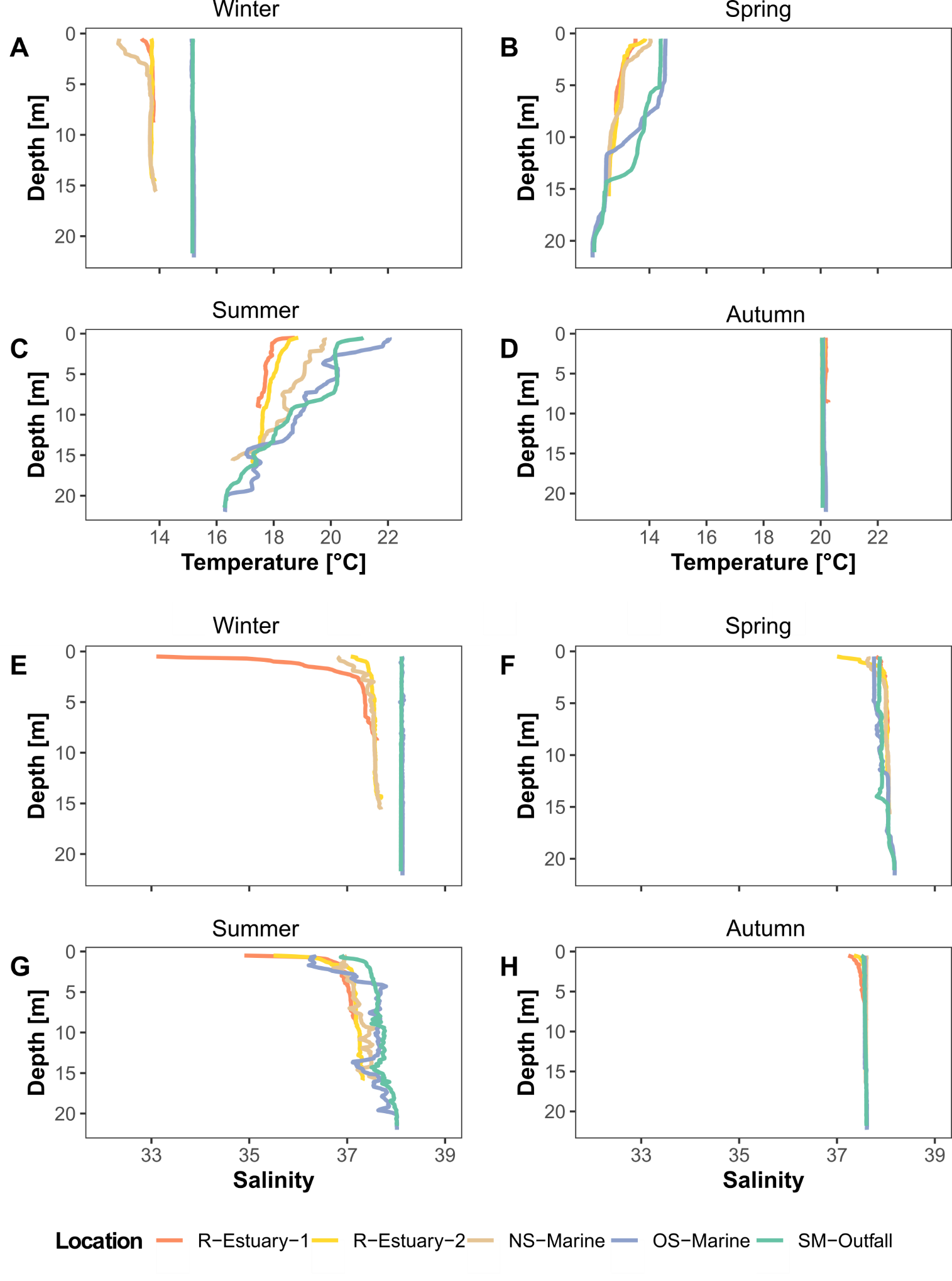
Supplementary Figure 2:** Vertical profiles of temperature **(A, B, C, D)** and salinity **(E, F, G, H)** at four seasonal samplings (winter, spring, summer, autumn) at 2018/2019 survey at 5 locations (R-Estuary-1, R-Estuary-2, NS-Marine, OS-Marine and SM-Outfall), measured with CTD probes prior sampling.


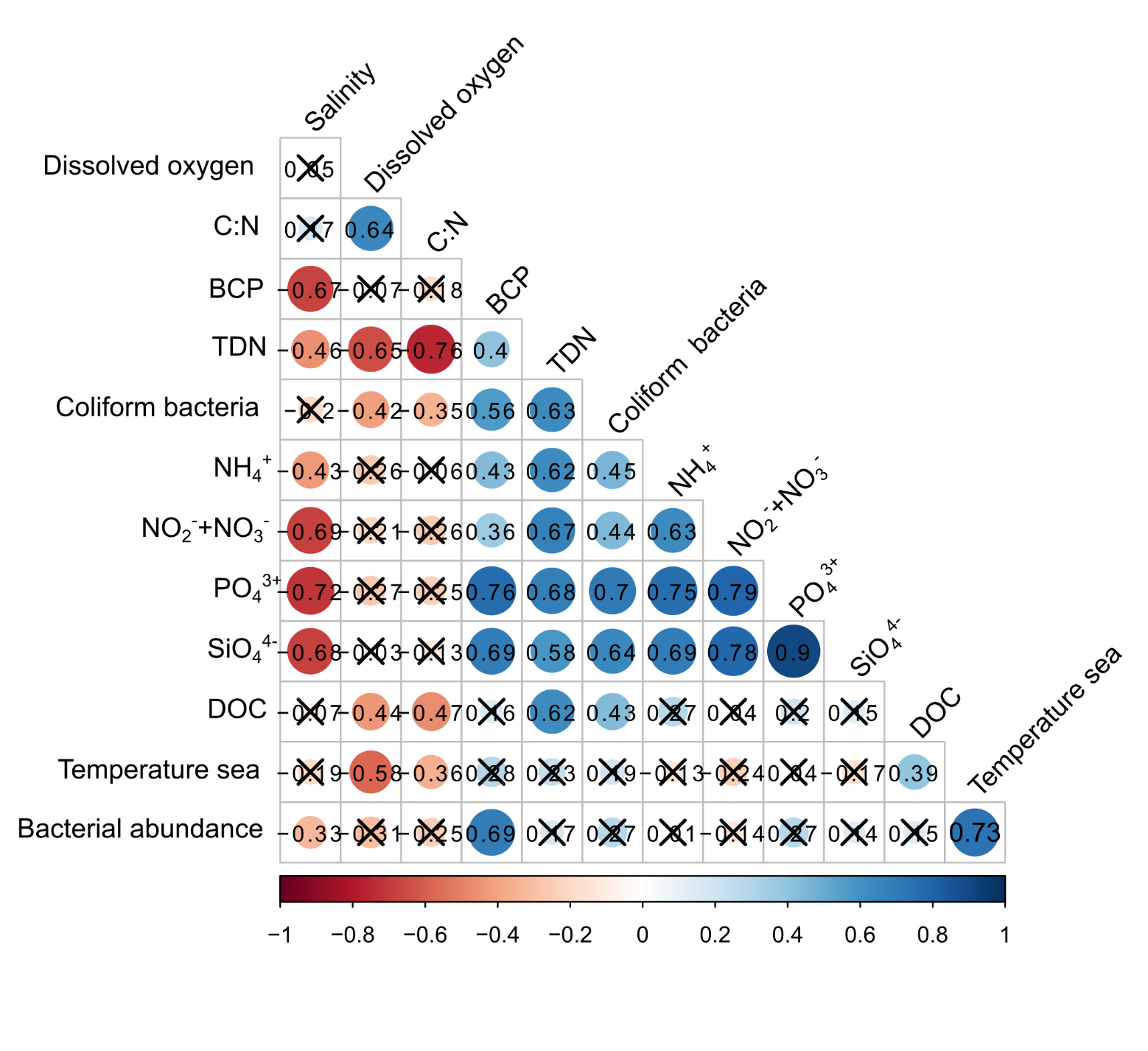


**Supplementary Figure 3:** Pearson correlation matrix. The legend shows the correlation coefficient and corresponding colors, insignificant correlations are crossed.


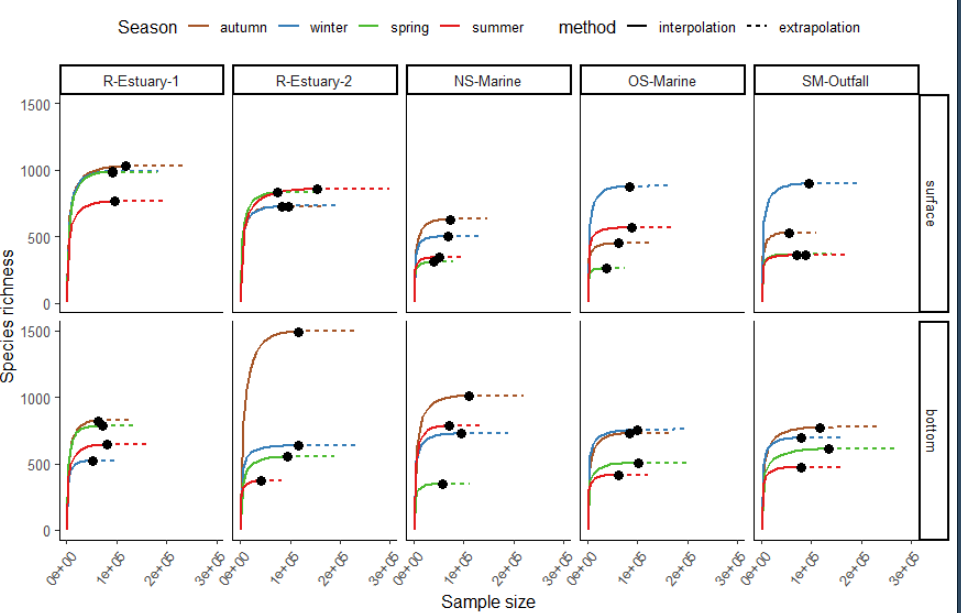


**Supplementary Figure 4:** Rarefactions of 16S rRNA gene analysis of bacterial communities. The solid lines represent the observed accumulation with the number of reads sampled, and the dashed lines represent the extrapolated accumulation up to the double amount of reads. The observed values for each community are denoted by solid shapes. Sample-size-based rarefaction curves generated with the R-package “iNEXT”, based on the Hill number of order q = 0. The rarefaction curves for each sample were generated based on 40 equally spaced rarefied sample sizes with 100 iterations.

**
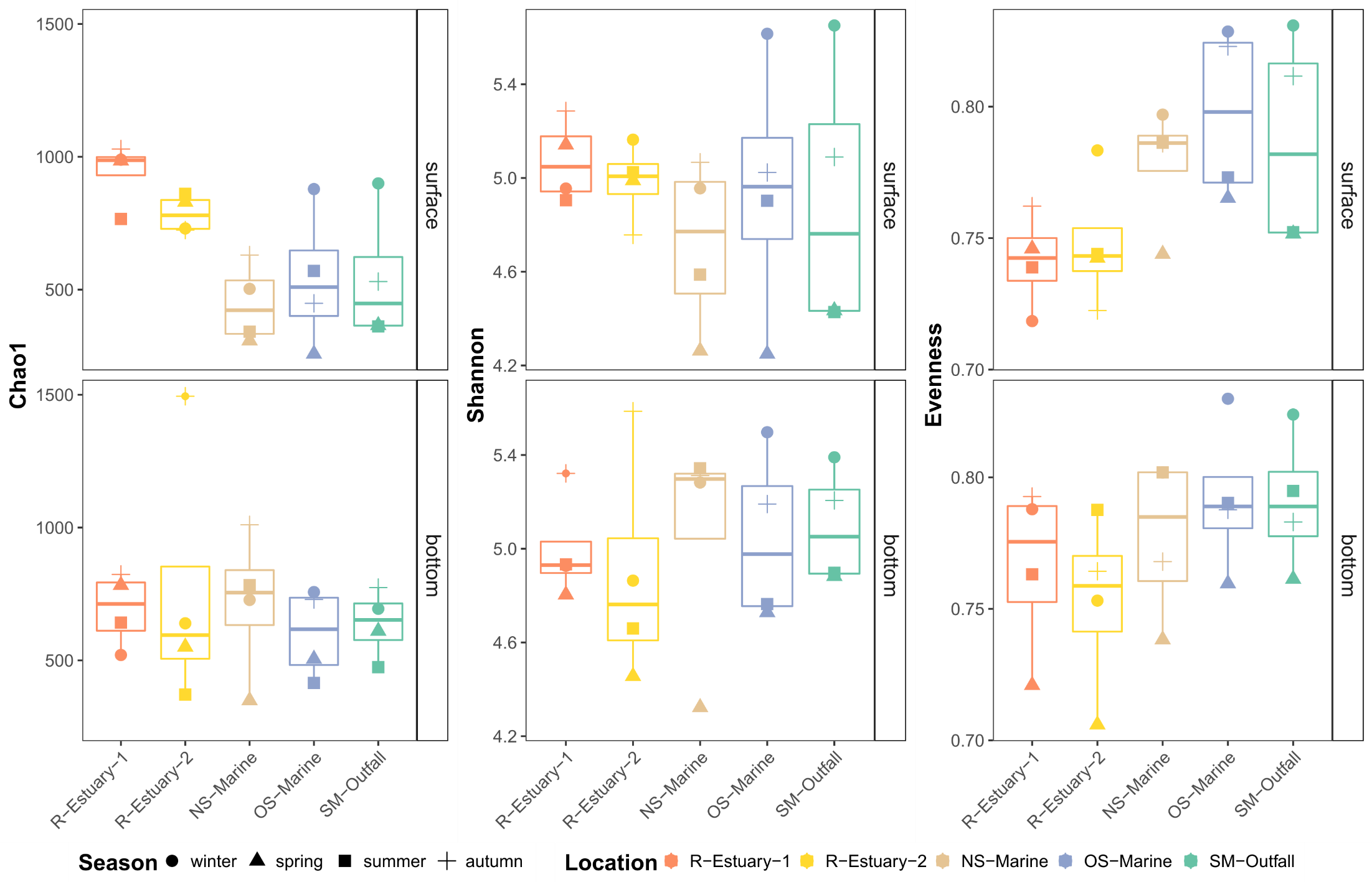
**

**Supplementary Figure 5:** Alpha diversity indexes at the surface and in the bottom layer in the winter, spring, summer and autumn during the 2018/2019 survey in the Gulf of Trieste at sampling stations: R-Estuary-1, R-Estuary-2, NS-Marine, OS-Marine and SM-Outfall. Presented are: Chao1 richness estimator, Shannon diversity index and species Evenness. Evenness is calculated as Shannon diversity index divided by natural logarithm of species richness. Different colors represents different sampling stations, while different shapes indicated sampling season.

**
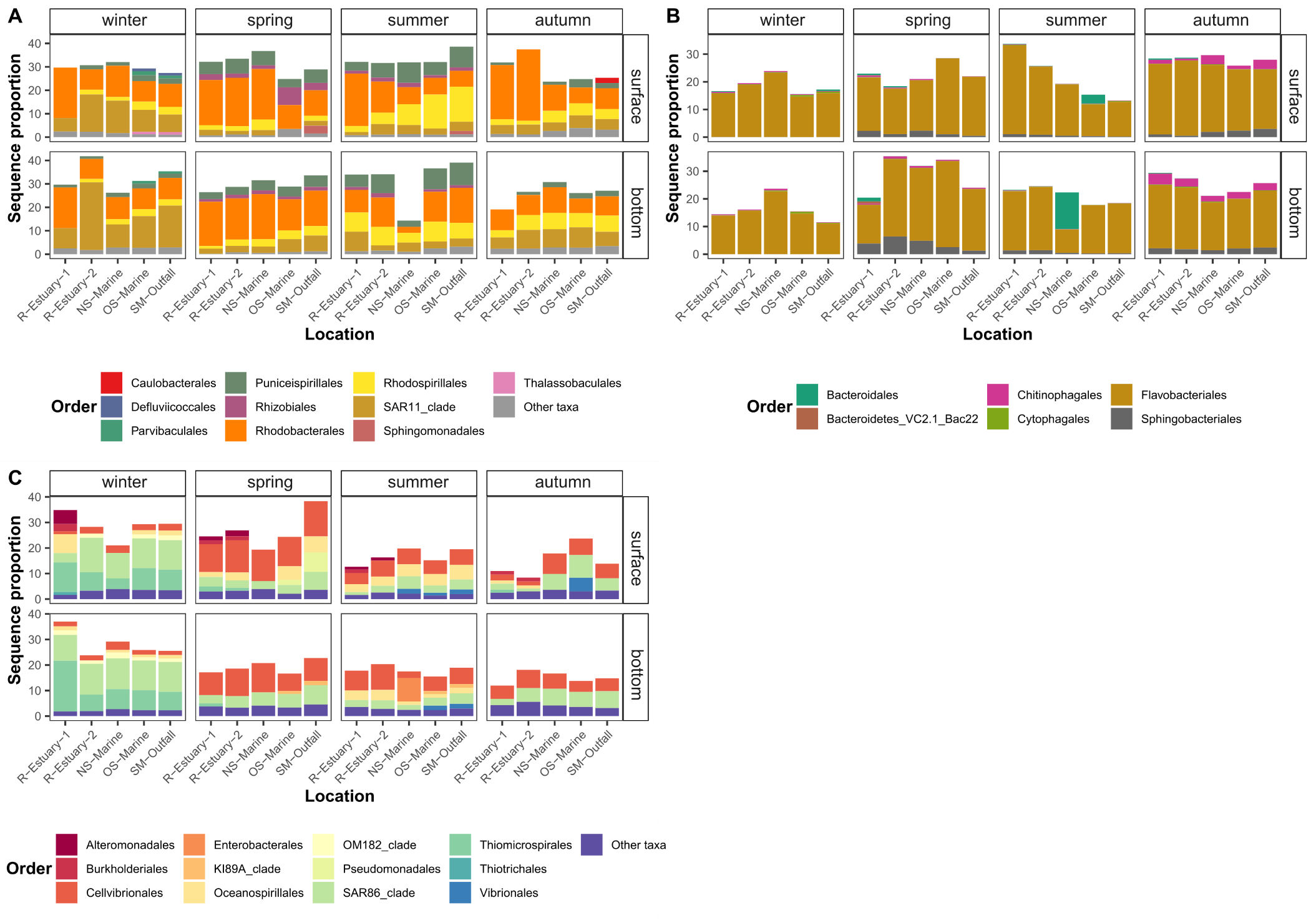
Supplementary Figure 6:** Sequence proportion of bacterial orders within dominant bacterial classes at the surface and in the bottom layer at sampling stations (R-Estuary-1, R-Estuary-2. NS-Marine, OS-Marine and SM-Outfall), in the winter, spring, summer, and autumn during the 2018/2019 survey. Presented are: Alphaproteobacteria **(A)** (bacterial orders showing a sequence proportion of <1 % were grouped together as “Other taxa”), Bacteroidia **(B)** and Gammaproteobacteria **(C)** (bacterial orders showing a sequence proportion of <1 % were grouped together as “Other taxa”).

**
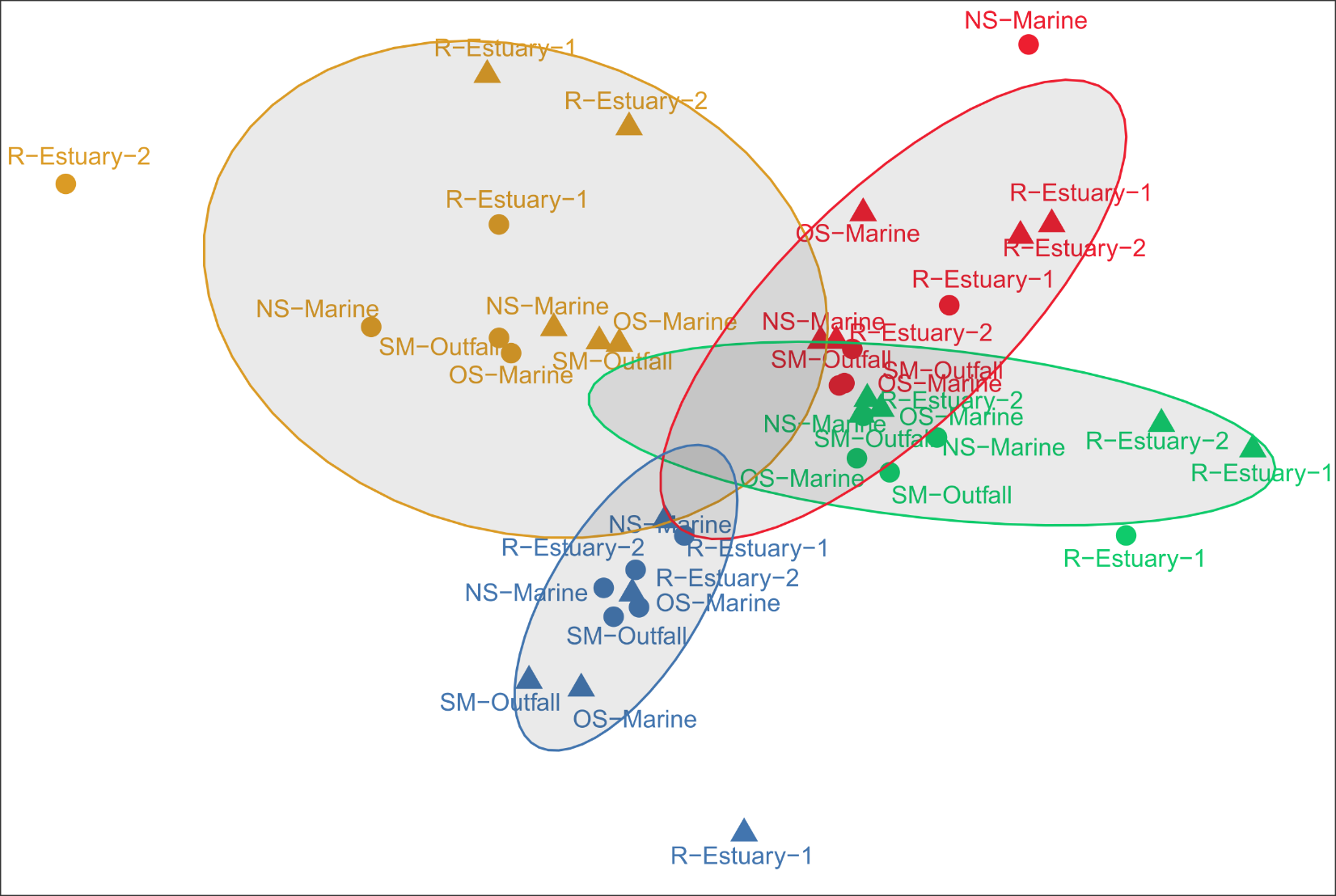
Supplementary Figure 7:** Non-metric multidimensional scaling (nMDS) ordination of bacterial community composition based on Euclidean distance matrix. The ellipses represent 0.95 confidence interval around the centroid of each group. Different colors represent sampling seasons, while shapes represents sampling depths.

**
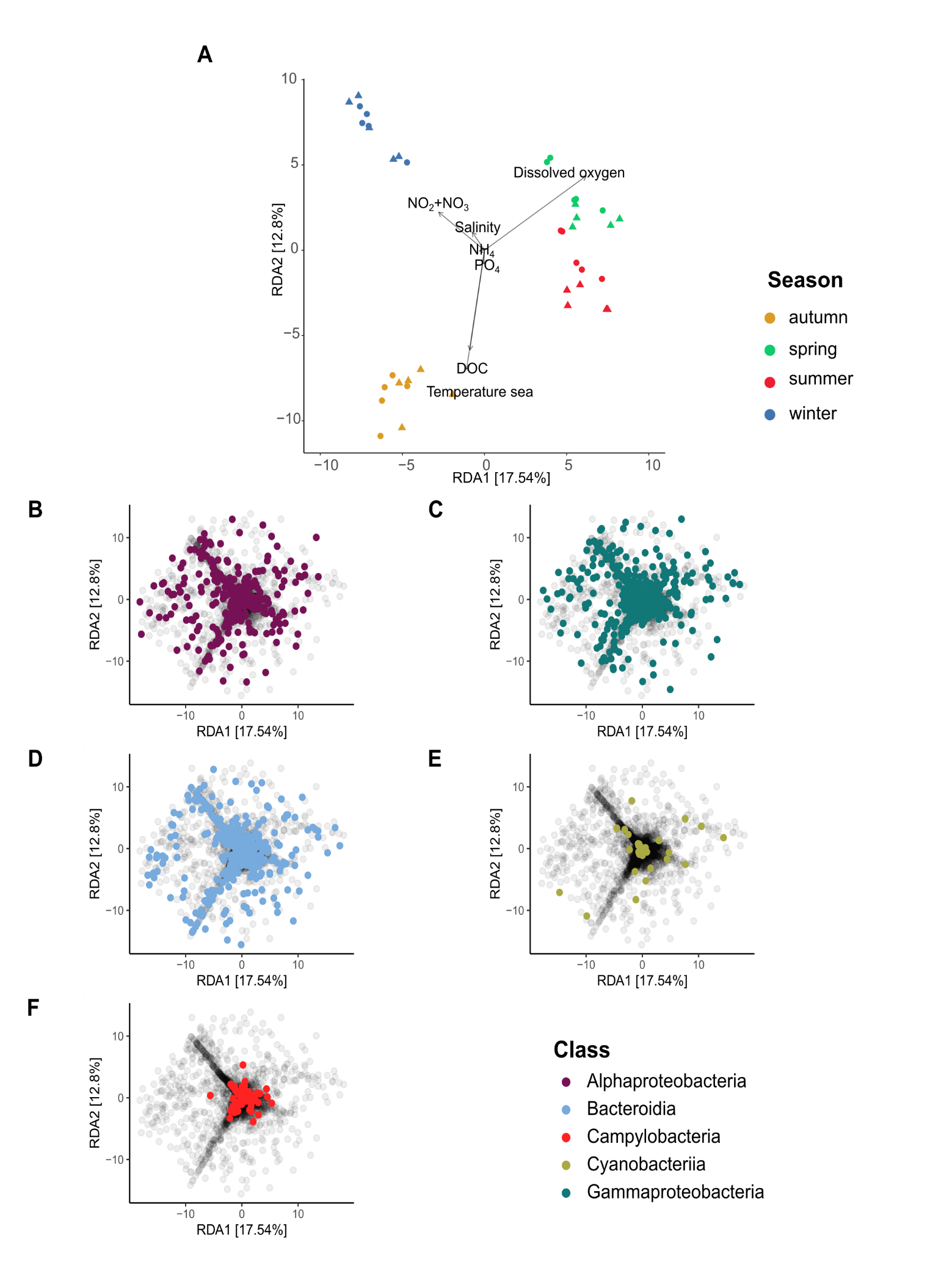
Supplementary Figure 8:** Redundancy analyses (RDA) ordination of variance-transformed data of bacterial community at 2018/2019 dataset constrained by environmental parameters: Temperature, Salinity, Dissolved Oxygen, sum of nitrite and nitrate (NO_2_^-^+NO_3_^-^), phosphate (PO_4_^3+^) and ammonium (NH_4_^+^). The percentage represents the explained variance of each axis. Presented ASVs assigned to dominant bacterial classes are presented at separate graphs

**
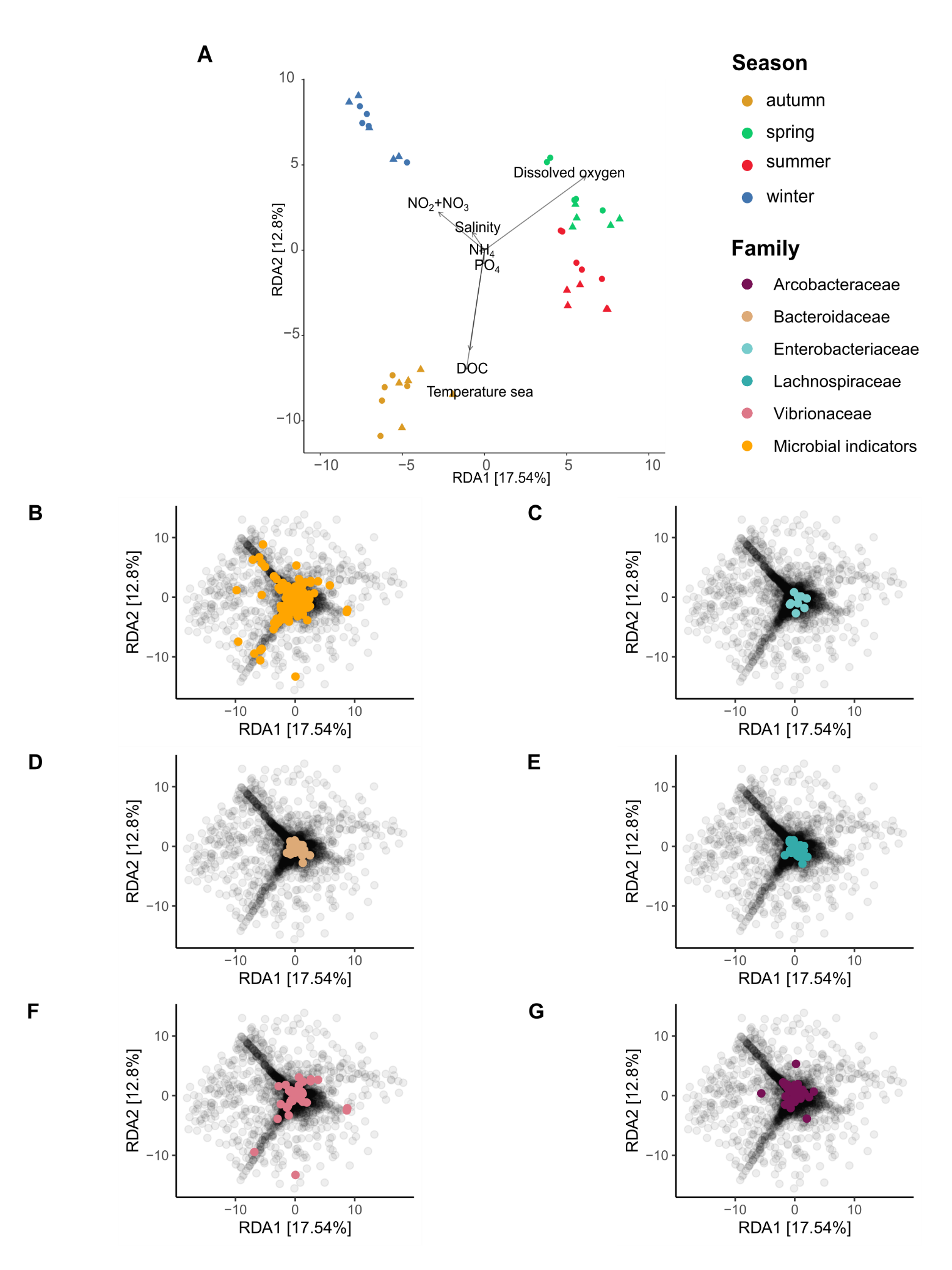
**

**Supplementary Figure 9:** Redundancy analyses (RDA) ordination of variance-transformed data of bacterial community at 2018/2019 dataset constrained by environmental parameters: Temperature, Salinity, Dissolved Oxygen, sum of nitrite and nitrate (NO_2_^-^+NO_3_^-^), phosphate (PO_4_^3+^) and ammonium (NH_4_^+^). The percentage represent the explained variance of each axis. Presented ASVs assigned to selected bacterial indicators of wastewater pollution are presented at separate graphs.

**
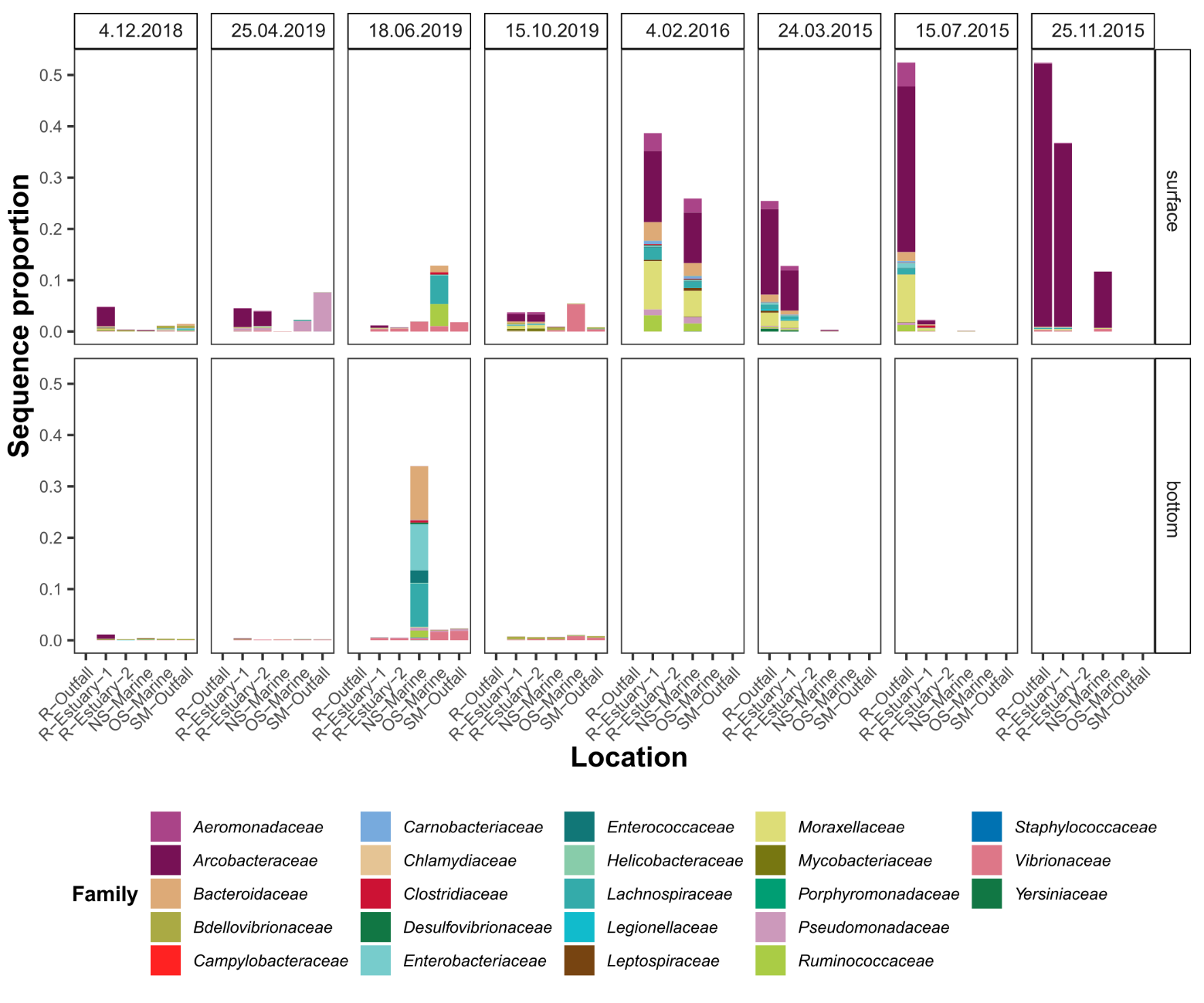
Supplementary Figure 10:** Sequence proportion of families belonging to bacterial indicators of wastewater pollution in winter, spring, summer, and autumn sampling during 2018/2019 *in situ* survey at sampling stations: R-Estuary-1, R-Estuary-2, NS-Marine, OS-Marine and SM-Outfall and during 2015/2016 *in situ* survey at sampling stations: R-Outfall, R-Estuary-1, NS-Marine.

**
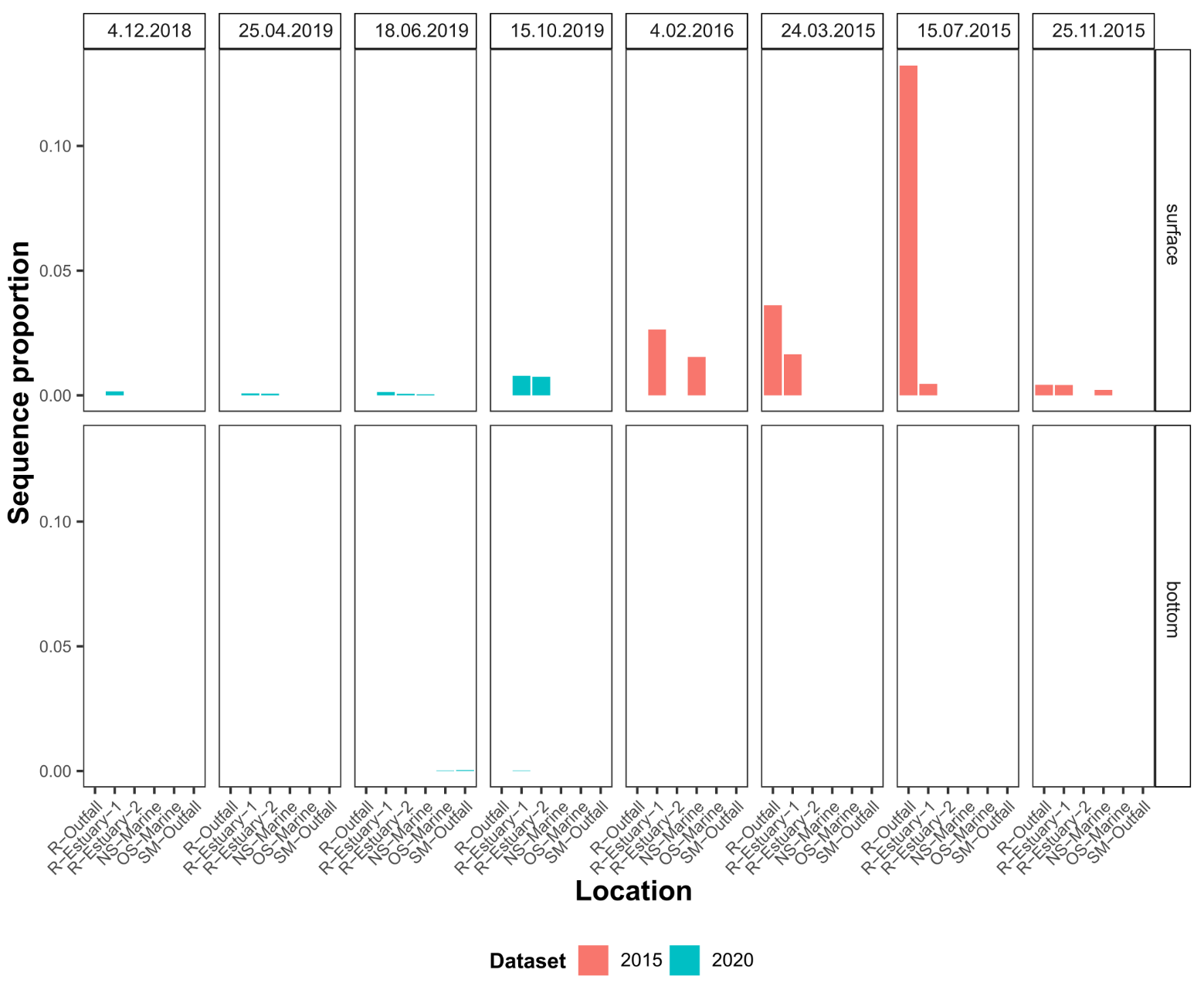
**

**Supplementary Figure 11:** Sequence proportion of ASV55 at sampling locations in winter, spring, summer, and autumn sampling during 2018/2019 *in situ* survey at sampling stations: R-Estuary-1, R-Estuary-2, NS-Marine, OS-Marine and SM-Outfall and during 2015/2016 *in situ* survey at sampling stations: R-Outfall, R-Estuary-1, NS-Marine.

**
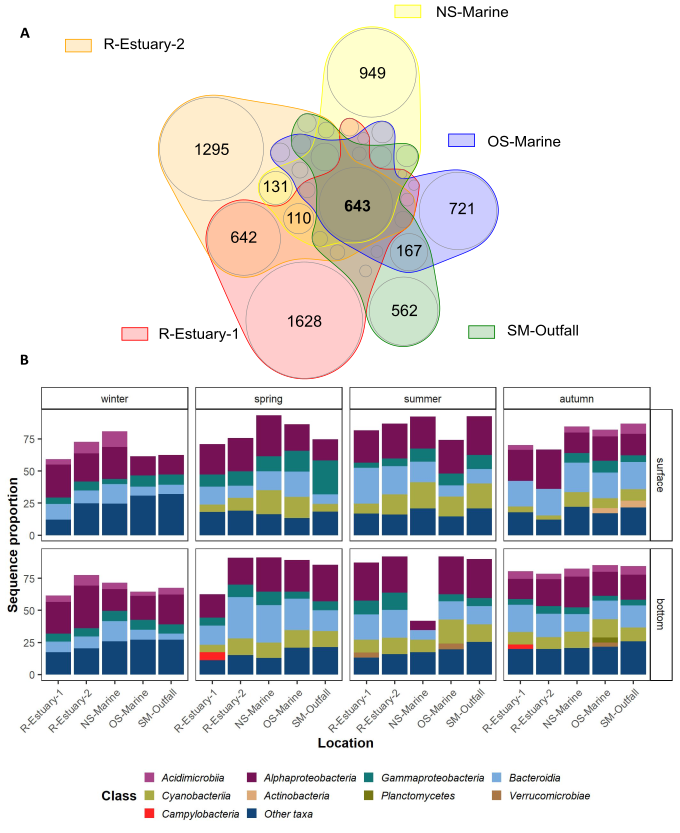
Supplementary Figure 12:** Visualization of relationships between bacterial communities at different stations based on presence/absence of ASVs in 2018/2019 dataset using a Venn diagram **(A)**. Analyses of presence/absence was done on overall data for specific location (all seasons and both depths together). Inserted circles are approximately proportional to the number of ASVs at each region. Numbers of shared ASVs between stations that are lower than 100 are not presented. The community composition of the core microbiome is presented in graph **B** (classes showing a relative sequence abundance of <3% were aggregated into the group “Other taxa”).


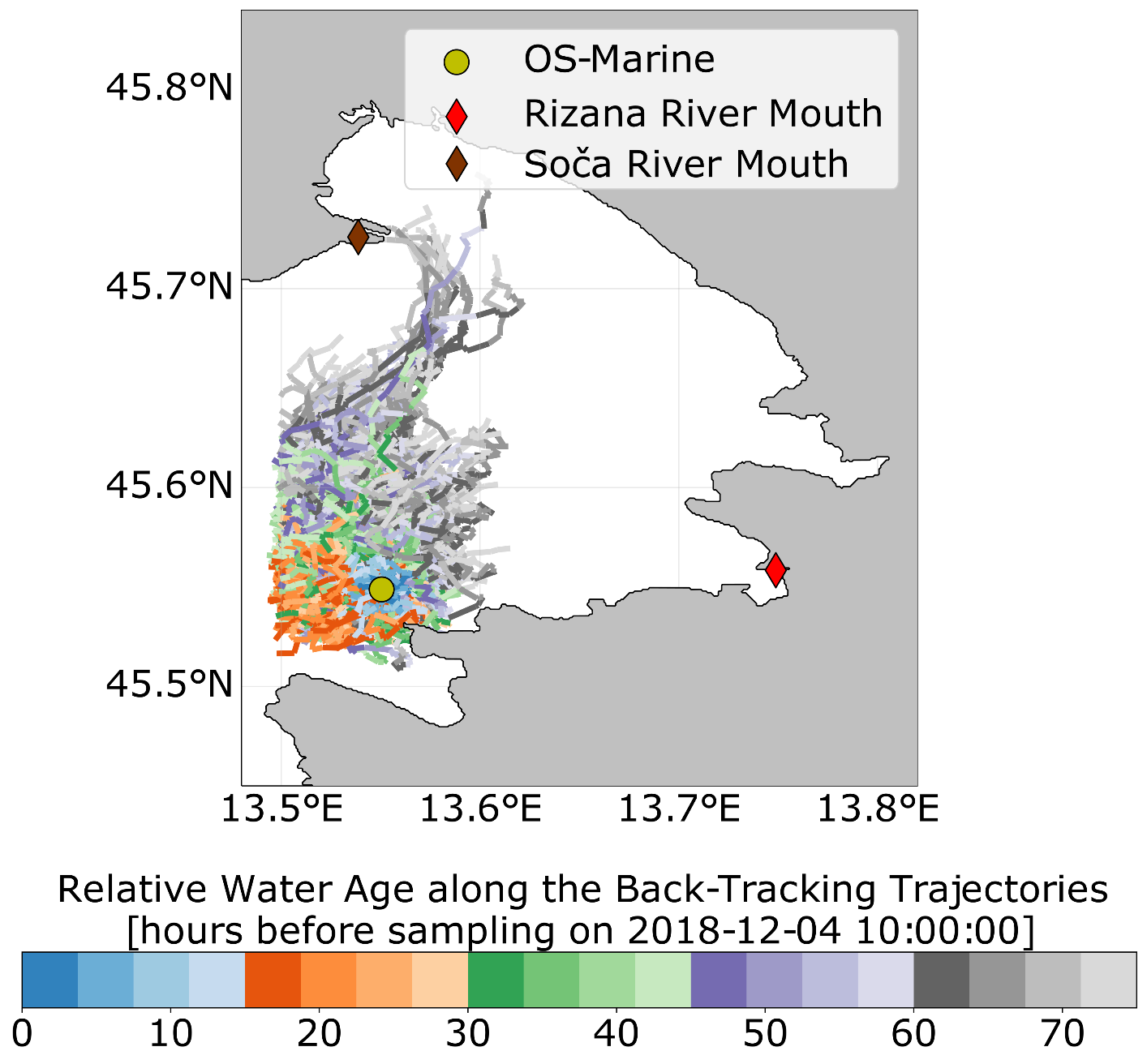


**Supplementary Figure 13:** Lagrangian back-tracking of water masses from OS-Marine sampling station. A sampling station is marked by a green circle, Rižana river mouth with the blue diamond and Soča river mouth with brown diamond. Trajectories indicate from where the water masses may have arrived to the sampling location prior to the respective sampling time. Each trajectory is colored by relative water age prior to the sampling time.

**Supplementary Table 1:** Microbial indicators of anthropogenic pollution selected based on different studies

| **Family** | **Genus** | **Indicator** | **Publication** |
| --- | --- | --- | --- |
| ***Enterobacteriaceae*** | *Escherichia-Shigella* | traditional fecal indicator | Luna et al., 2016, Maugeri et al., 2004 |
| ***Enterococcaceae*** | *Enterococcus* | traditional fecal indicator | Luna et al., 2016, Maugeri et al., 2004 |
| ***Bacteroidaceae*** | *Bacteroides* | Faeces-associated | Luna et al., 2016, Newton et al., 2013, Buccheri et al., 2019 |
| ***Clostridiaceae*** |  | Faeces-associated | Luna et al., 2016, Newton et al., 2013, Buccheri et al., 2019 |
| ***Lachnospiraceae*** |  | Faeces-associated | Luna et al., 2016, Newton et al., 2013, Buccheri et al., 2019 |
| ***Porphyromonadaceae*** |  | Faeces-associated | Luna et al., 2016, Newton et al., 2013, Buccheri et al., 2019 |
| ***Ruminococcaceae*** |  | Faeces-associated | Luna et al., 2016, Newton et al., 2013, Buccheri et al., 2019 |
| ***Aeromonadaceae*** | *Aeromonas* | Sewage-associated | Maugeri et al., 2004 |
| ***Arcobacteraceae*** | *Arcobacter* | Sewage-associated | Luna et al., 2016, Maugeri et al., 2004, Newton et al., 2013, Buccheri et al., 2019 |
| ***Bdellovibrionaceae*** | *Bdellovibrio* | Sewage-associated | Jurkevitch, 2020 |
| ***Campylobacteraceae*** | *Campylobacter* | Sewage-associated | Numberger et al., 2019, Maugeri et al., 2004, Naidoo and Olaniran, 2013 |
| ***Carnobacteriaceae*** | *Trichococcus* | Sewage-associated | Luna et al., 2016, Newton et al., 2013, Buccheri et al., 2019 |
| ***Clostridiaceae*** | *Clostridium* | Sewage-associated | Numberger et al., 2019, Naidoo and Olaniran, 2013 |
| ***Desulfovibrionaceae*** | *Desulfovibrio* | Sewage-associated | El Houari et al., 2020; Kuever et al., 2015 |
| ***Enterobacteriaceae*** | *Salmonella* | Sewage-associated | Numberger et al., 2019, Naidoo and Olaniran, 2013 |
| ***Enterobacteriaceae*** | *Escherichia-Shigella* | Sewage-associated | Numberger et al., 2019, Naidoo and Olaniran, 2013 |
| ***Helicobacteraceae*** | *Helicobacter* | Sewage-associated | Li, 2015 |
| ***Legionellaceae*** | *Legionella* | Sewage-associated | Numberger et al., 2019, Stewart et al., 2008 |
| ***Leptospiraceae*** | *Leptospira* | Sewage-associated | Numberger et al., 2019 |
| ***Listeriaceae*** | *Listeria* | Sewage-associated | Stewart et al., 2008, Li, 2015 |
| ***Moraxellaceae*** | *Acinetobacter* | Sewage-associated | Luna et al., 2016, Newton et al., 2013, Buccheri et al., 2019 |
| ***Mycobacteriaceae*** | *Mycobacterium* | Sewage-associated | Numberger et al., 2019 |
| ***Staphylococcaceae*** | *Staphylococcus* | Sewage-associated | Stewart et al., 2008, Li, 2015 |
| ***Vibrionaceae*** | *Vibrio* | Sewage-associated | Numberger et al., 2019, Maugeri et al., 2004, Naidoo and Olaniran, 2013 |
| ***Yersiniaceae*** | *Yersinia* | Sewage-associated | Numberger et al., 2019 |
| ***Pseudomonadaceae*** | *Pseudomonas* | Sewage-associated | Stewart et al., 2008, Luckiewicz et al. 2015 |
| ***Chlamydiaceae*** | *Chlamydia* | Sewage-associated | Stewart et al., 2008; Collingro et al. 2020 |

**Supplementary Table 2:** Sampling information, sea level and Rižana river flow rate at time of sampling. (Rižana flow rate – source: Hidrolog, sea level – sea levels at the tide gauge Koper)

| **Location** | **Sampling date [dd-mm-yyyy]** | **Sampling season** | **Sampling depth [m]** | **Sampling time [hh:mm]** | **Latitude** | **Longitude** | **Sea level in Kp [cm] (time)** | **Rižana river flow rate [m3/s] (time)** |
| --- | --- | --- | --- | --- | --- | --- | --- | --- |
| R Outfall | 24.03.2015 | spring | 0 |  | 45°33′27.6′′N | 13°45′36.48′′E |  |  |
| R-Estuary-1 | 24.03.2015 | spring | 0 |  | 45°33'31.00"N | 13°44'37.30"E |  |  |
| NS-Marine | 24.03.2015 | spring | 0 |  | 45°33'34.70"N | 13°43'11.90"E |  |  |
| R Outfall | 15.07.2015 | summer | 0 |  | 45°33′27.6′′N | 13°45′36.48′′E |  |  |
| R-Estuary-1 | 15.07.2015 | summer | 0 |  | 45°33'31.00"N | 13°44'37.30"E |  |  |
| NS-Marine | 15.07.2015 | summer | 0 |  | 45°33'34.70"N | 13°43'11.90"E |  |  |
| R Outfall | 25.11.2015 | autumn | 0 |  | 45°33′27.6′′N | 13°45′36.48′′E |  |  |
| R-Estuary-1 | 25.11.2015 | autumn | 0 |  | 45°33'31.00"N | 13°44'37.30"E |  |  |
| NS-Marine | 25.11.2015 | autumn | 0 |  | 45°33'34.70"N | 13°43'11.90"E |  |  |
| R-Estuary-1 | 04.02.2016 | winter | 0 |  | 45°33'31.00"N | 13°44'37.30"E |  |  |
| NS-Marine | 04.02.2016 | winter | 0 |  | 45°33'34.70"N | 13°43'11.90"E |  |  |
| OS-Marine | 04.12.2018 | winter | 0 and 21 | 10:00 | 45°32'55.50"N | 13°33'2.52"E | 246.5 (10:00) | 1.838 (10:00) |
| SM-Outfall | 04.12.2018 | winter | 0 and 21 | 09:20 | 45°32'1.80"N | 13°31'51.00"E | 260.5 (9:20) | 1.63 (9:00) |
| NS-Marine | 04.12.2018 | winter | 0 and 15 | 11:35 | 45°33'34.70"N | 13°43'11.90"E | 233 (10:30) | 1.63 (12:00) |
| R-Estuary-2 | 04.12.2018 | winter | 0 and 14 | 12:15 | 45°33'29.70"N | 13°44'4.60"E | 200.5 (12:10) | 1.63 (12:00) |
| R-Estuary-1 | 04.12.2018 | winter | 0 and 8 | 11:50 | 45°33'31.00"N | 13°44'37.30"E | 227 (10:50) | 1.63 (12:00) |
| OS-Marine | 25.04.2019 | spring | 0 and 21 | 08:50 | 45°32'55.50"N | 13°33'2.52"E | 209.4 (8:50) | 1.255 (9:00) |
| SM-Outfall | 25.04.2019 | spring | 0 and 21 | 08:30 | 45°32'1.80"N | 13°31'51.00"E | 206.5 (8:30) | 1.255 (8:00, 9:00) |
| NS-Marine | 25.04.2019 | spring | 0 and 15 | 09:50 | 45°33'34.70"N | 13°43'11.90"E | 222.1 (9:50) | 1.435 (10:00) |
| R-Estuary-2 | 25.04.2019 | spring | 0 and 15 | 11:00 | 45°33'29.70"N | 13°44'4.60"E | 233.3 (11:00) | 1.435 (11:00) |
| R-Estuary-1 | 25.04.2019 | spring | 0 and 7 | 11:30 | 45°33'31.00"N | 13°44'37.30"E | 235.5 (11:30) | 1.435  (11:00, 12:00) |
| OS-Marine | 18.06.2019 | summer | 0 and 21 | 10:35 | 45°32'55.50"N | 13°33'2.52"E | 245.8 (10:30) | 0.366 (11:00) |
| SM-Outfall | 18.06.2019 | summer | 0 and 21 | 10:00 | 45°32'1.80"N | 13°31'51.00"E | 244.0 (10:00) | 0.291 (10:00) |
| NS-Marine | 18.06.2019 | summer | 0 and 15 | 13:55 | 45°33'34.70"N | 13°43'11.90"E | 227.5 (13:50) | 0.291 (14:00) |
| R-Estuary-2 | 18.06.2019 | summer | 0 and 16 | 13:30 | 45°33'29.70"N | 13°44'4.60"E | 230.9 (13:30) | 0.291  (13:00, 14:00) |
| R-Estuary-1 | 18.06.2019 | summer | 0 and 8 | 13:15 | 45°33'31.00"N | 13°44'37.30"E | 233.7 (13:10) | 0.291 (13:00) |
| OS-Marine | 15.10.2019 | autumn | 0 and 22 | 09:30 | 45°32'55.50"N | 13°33'2.52"E | 283.3 (9:30) | 0.23 (9:00),  0.366 (10:00) |
| SM-Outfall | 15.10.2019 | autumn | 0 and 22 | 08:50 | 45°32'1.80"N | 13°31'51.00"E | 280.6 (8:50) | 0.23 (9:00) |
| NS-Marine | 15.10.2019 | autumn | 0 and 16 | 11:00 | 45°33'34.70"N | 13°43'11.90"E | 278.1 (11:00) | 0.366 (11:00) |
| R-Estuary-2 | 15.10.2019 | autumn | 0 and 16 | 11:30 | 45°33'29.70"N | 13°44'4.60"E | 268.3 (11:30) | 0.366 (11:00),  0.291 (12:00) |
| R-Estuary-1 | 15.10.2019 | autumn | 0 and 8 | 11:45 | 45°33'31.00"N | 13°44'37.30"E | 265.6 (11:40) | 0.291 (12:00) |

**Supplementary Table 3:** Dynamics of abiotic and biotic environmental parameters in 2015/2016 dataset

| **Date** | **Location** | **Temperature sea** | **Salinity** | **Dissolved Oxygen** | **DOC** | **DIN** | **NO_2_^-^ + NO_3_^-^** | **PO_4_^3+^** | **NH_4_^+^** | **SiO_3_** | **Coliform bacteria** |
| --- | --- | --- | --- | --- | --- | --- | --- | --- | --- | --- | --- |
| 24.03.2015 | R-Mouth | 8.3 | 10.9 | 9.92 |  |  |  |  |  |  |  |
| 24.03.2015 | R-Estuary-1 | 10.4 | 21.4 | 8.45 |  |  |  |  |  |  |  |
| 24.03.2015 | NS-Marine | 10.5 | 37 | 7.62 |  |  |  |  |  |  |  |
| 15.07.2015 | R-Mouth | 21.7 | 14.1 | 7.55 |  |  |  |  |  |  | 88 |
| 15.07.2015 | R-Estuary-1 | 23.9 | 32.9 | 6.6 |  |  |  |  |  |  | 22 |
| 15.07.2015 | NS-Marine | 24.3 | 35.4 | 7.06 |  |  |  |  |  |  | 4 |
| 25.11.2015 | R-Mouth | 9.8 | 5.2 | 10.01 | 146.8 | 29.8 | 28.44 | 0.69 | 1.33 | 31.86 | 348 |
| 25.11.2015 | R-Estuary-1 | 10.8 | 13.9 | 8.47 | 107.8 | 7.6 | 6.04 | 0.38 | 1.6 | 7.12 | 110 |
| 25.11.2015 | NS-Marine | 14.3 | 36.6 | 7.33 | 124.1 | 5.7 | 4.11 | 0.21 | 1.56 | 4.97 | 44 |
| 4.02.2016 | R-Estuary-1 | 10 | 12.6 | 10.15 | 115.4 | 43.3 | 18.93 | 0.76 | 24.33 | 47.28 | 756 |
| 4.02.2016 | NS-Marine | 10.3 | 29 | 9.12 | 125.2 | 53.3 | 29.14 | 0.74 | 24.17 | 24.34 | 124 |

**Supplementary Table 4:** Overview of samples with sampling information, number of sequences at each step of bioinformatics analyses and alpha diversity calculations.

| **Location** | **Sampling date [dd-mm-yyyy]** | **Depth [m]** | **No. Of raw amplicons** | **No. Of amplicons after QC** | **No. Of amplicons after merging** | **No. Of amplicons after chimera removal** | **Final no. Of amplicons after taxonomic filtering** | **Observed richenss (number of ASVs)** | **Chao richness estimator** | **Shannon diversity index** | **InvSimpson index** | **Evenness** |
| --- | --- | --- | --- | --- | --- | --- | --- | --- | --- | --- | --- | --- |
| OS-Marine | 04.12.2018 | 0 | 121947 | 95792 | 92067 | 84652 | 72195 | 751 | 751.2 | 5.43 | 107.29 | 0.82 |
| SM-Outfall | 04.12.2018 | 0 | 132807 | 106370 | 101640 | 96331 | 83133 | 785 | 785.52 | 5.48 | 113.2 | 0.82 |
| NS-Marine | 04.12.2018 | 0 | 92212 | 76633 | 71127 | 67774 | 58330 | 447 | 448 | 4.91 | 64.13 | 0.8 |
| R-Estuary-2 | 04.12.2018 | 0 | 145801 | 116919 | 105239 | 97027 | 76851 | 627 | 628.56 | 5.08 | 72.14 | 0.79 |
| R-Estuary-1 | 04.12.2018 | 0 | 141367 | 109473 | 95985 | 91530 | 85294 | 910 | 910.09 | 4.82 | 31.94 | 0.71 |
| OS-Marine | 04.12.2018 | 21 | 149512 | 121412 | 115022 | 99013 | 81192 | 618 | 619.87 | 5.3 | 98.45 | 0.82 |
| SM-Outfall | 04.12.2018 | 21 | 118530 | 96913 | 87813 | 79315 | 63125 | 567 | 567.26 | 5.17 | 86.76 | 0.81 |
| NS-Marine | 04.12.2018 | 15 | 125485 | 102583 | 99342 | 94337 | 80093 | 629 | 630.25 | 5.25 | 93 | 0.81 |
| R-Estuary-2 | 04.12.2018 | 14 | 176351 | 145116 | 132629 | 117031 | 98434 | 540 | 540.59 | 4.75 | 46.24 | 0.76 |
| R-Estuary-1 | 04.12.2018 | 8 | 81227 | 65003 | 55672 | 51337 | 43248 | 453 | 453.18 | 4.8 | 47.99 | 0.79 |
| OS-Marine | 25.04.2019 | 0 | 47353 | 38176 | 37509 | 37177 | 34981 | 212 | 212 | 4.06 | 31.56 | 0.76 |
| SM-Outfall | 25.04.2019 | 0 | 90882 | 73172 | 72107 | 70415 | 67503 | 311 | 311.6 | 4.29 | 33.32 | 0.75 |
| NS-Marine | 25.04.2019 | 0 | 51729 | 41276 | 39935 | 39424 | 35902 | 254 | 254.33 | 4.04 | 20.69 | 0.73 |
| R-Estuary-2 | 25.04.2019 | 0 | 96963 | 78884 | 74318 | 73339 | 65852 | 763 | 763.67 | 4.82 | 41.69 | 0.73 |
| R-Estuary-1 | 25.04.2019 | 0 | 128420 | 98699 | 92045 | 90469 | 81129 | 909 | 909.21 | 4.97 | 43.34 | 0.73 |
| OS-Marine | 25.04.2019 | 21 | 132032 | 107999 | 104266 | 101792 | 91925 | 405 | 405.06 | 4.49 | 38.05 | 0.75 |
| SM-Outfall | 25.04.2019 | 21 | 177198 | 142203 | 138285 | 135390 | 112145 | 473 | 473.65 | 4.58 | 37.13 | 0.74 |
| NS-Marine | 25.04.2019 | 15 | 72778 | 58932 | 57072 | 56561 | 53764 | 297 | 297.2 | 4.16 | 25.09 | 0.73 |
| R-Estuary-2 | 25.04.2019 | 15 | 122030 | 99059 | 95474 | 94507 | 87835 | 476 | 476.03 | 4.26 | 26.74 | 0.69 |
| R-Estuary-1 | 25.04.2019 | 7 | 106983 | 87953 | 76510 | 70731 | 64431 | 713 | 713.05 | 4.64 | 26.13 | 0.71 |
| OS-Marine | 18.06.2019 | 0 | 119873 | 94095 | 89719 | 88012 | 81818 | 498 | 499.67 | 4.76 | 39.01 | 0.77 |
| SM-Outfall | 18.06.2019 | 0 | 118690 | 93043 | 90218 | 88723 | 82075 | 276 | 278 | 4.23 | 29.84 | 0.75 |
| NS-Marine | 18.06.2019 | 0 | 65055 | 51008 | 49665 | 49374 | 45867 | 282 | 282 | 4.4 | 39.22 | 0.78 |
| R-Estuary-2 | 18.06.2019 | 0 | 209521 | 162341 | 155777 | 153755 | 141441 | 731 | 732.29 | 4.82 | 57.16 | 0.73 |
| R-Estuary-1 | 18.06.2019 | 0 | 133295 | 104278 | 97950 | 96357 | 93060 | 710 | 711.57 | 4.81 | 50.37 | 0.73 |
| OS-Marine | 18.06.2019 | 21 | 84229 | 68148 | 63795 | 60897 | 56138 | 342 | 342.3 | 4.57 | 39.68 | 0.78 |
| SM-Outfall | 18.06.2019 | 21 | 109856 | 87584 | 82646 | 79547 | 73482 | 389 | 389.33 | 4.7 | 41.09 | 0.79 |
| NS-Marine | 18.06.2019 | 15 | 101414 | 81341 | 74924 | 70193 | 68244 | 741 | 741 | 5.27 | 86.63 | 0.8 |
| R-Estuary-2 | 18.06.2019 | 16 | 60896 | 49388 | 43493 | 41706 | 39331 | 322 | 322.2 | 4.51 | 48.66 | 0.78 |
| R-Estuary-1 | 18.06.2019 | 8 | 106016 | 86171 | 81160 | 80318 | 76281 | 578 | 578 | 4.82 | 62.58 | 0.76 |
| OS-Marine | 15.10.2019 | 0 | 80901 | 63948 | 62645 | 62239 | 61816 | 428 | 428.43 | 5 | 77.65 | 0.82 |
| SM-Outfall | 15.10.2019 | 0 | 70949 | 57514 | 55349 | 54971 | 52755 | 467 | 468.47 | 4.98 | 72.84 | 0.81 |
| NS-Marine | 15.10.2019 | 0 | 93660 | 76097 | 73854 | 73412 | 70205 | 563 | 563.15 | 4.96 | 64.54 | 0.78 |
| R-Estuary-2 | 15.10.2019 | 0 | 123331 | 97419 | 87693 | 83572 | 81258 | 660 | 660.32 | 4.66 | 33.39 | 0.72 |
| R-Estuary-1 | 15.10.2019 | 0 | 162830 | 126579 | 118935 | 116992 | 105118 | 926 | 927.12 | 5.19 | 60.06 | 0.76 |
| OS-Marine | 15.10.2019 | 22 | 109874 | 88577 | 85849 | 84622 | 79032 | 648 | 648.71 | 5.06 | 73.04 | 0.78 |
| SM-Outfall | 15.10.2019 | 22 | 152306 | 122828 | 119198 | 117848 | 110803 | 673 | 675.28 | 5.06 | 67.74 | 0.78 |
| NS-Marine | 15.10.2019 | 16 | 146050 | 118499 | 111601 | 109970 | 99258 | 866 | 866.08 | 5.07 | 54.66 | 0.75 |
| R-Estuary-2 | 15.10.2019 | 16 | 155853 | 126186 | 118769 | 117012 | 102967 | 1338 | 1338.37 | 5.38 | 78.48 | 0.75 |
| R-Estuary-1 | 15.10.2019 | 8 | 89520 | 71877 | 65071 | 63993 | 58166 | 746 | 746.42 | 5.18 | 78.87 | 0.78 |

**Dataset 2015/2016**

| **Location** | **Sampling date [dd-mm-yyyy]** | **Depth [m]** | **No. Of raw amplicons** | **No. Of amplicons after QC** | **No. Of amplicons after merging** | **No. Of amplicons after chimera removal** | **Final no. Of amplicons after taxonomic filtering** | **Observed richens (number of ASVs)** | **Chao richness estimator** | **Shannon diversity index** | **InvSimpson index** | **Evenness** |
| --- | --- | --- | --- | --- | --- | --- | --- | --- | --- | --- | --- | --- |
| R-Mouth | 24.03.2015 | 0 | 125452 | 69666 | 55558 | 48789 | 48234 | 628 | 628 | 4.88 | 38.18 | 0.76 |
| R-Estuary-1 | 24.03.2015 | 0 | 185751 | 110729 | 93790 | 83611 | 81589 | 724 | 724.62 | 4.93 | 45.67 | 0.75 |
| NS-Marine | 24.03.2015 | 0 | 136802 | 86893 | 81186 | 75475 | 69973 | 346 | 347 | 4.48 | 41.63 | 0.77 |
| R-Mouth | 15.07.2015 | 0 | 121868 | 77398 | 61513 | 54716 | 54462 | 909 | 909.84 | 5.12 | 32.25 | 0.75 |
| R-Estuary-1 | 15.07.2015 | 0 | 93226 | 53743 | 47657 | 44712 | 40080 | 524 | 524.19 | 4.79 | 44.48 | 0.77 |
| NS-Marine | 15.07.2015 | 0 | 170166 | 101098 | 93284 | 87232 | 81910 | 295 | 295.12 | 4.14 | 26.58 | 0.73 |
| R-Mouth | 25.11.2015 | 0 | 258129 | 163114 | 142439 | 102366 | 102085 | 423 | 423.4 | 3.25 | 8.02 | 0.54 |
| R-Estuary-1 | 25.11.2015 | 0 | 153955 | 97207 | 84711 | 70494 | 69508 | 526 | 526 | 3.96 | 11.92 | 0.63 |
| NS-Marine | 25.11.2015 | 0 | 99321 | 53670 | 47261 | 41455 | 41236 | 277 | 277.46 | 2.7 | 3.34 | 0.48 |
| R-Estuary-1 | 04.02.2016 | 0 | 116577 | 70793 | 58366 | 52508 | 52190 | 1002 | 1002.76 | 5.88 | 92.34 | 0.85 |
| NS-Marine | 04.02.2016 | 0 | 124910 | 72364 | 51134 | 43133 | 42766 | 1100 | 1102.68 | 6.2 | 180.2 | 0.89 |

**Supplementary Table 5:** Bacterial abundance determined with flow cytometer measurements and number of total coliforms determined with cultured based approach.

| **Date** | **Location** | **Bottom** | | | | **Surface** | | | |
| --- | --- | --- | --- | --- | --- | --- | --- | --- | --- |
|  |  | **BA [N cell / L]** | **BA_STDEV** | **Total Coliforms [CFU/100 mL]** | **STDEV** | **BA [N cell / L]** | **STDEV** | **Total Coliforms [CFU/100 mL]** | **STDEV** |
| 4.12.2018 | R-Estuary-1 | 8.54E+08 | 8.37E+07 | **7** | 1 | 7.89E+08 | 3.17E+07 | **189** | 7 |
|  | R-Estuary-2 | 8.76E+08 | 3.22E+07 | **1** | 1 | 5.69E+08 | 7.99E+07 | **11** | 7 |
|  | NS-Marine | 5.53E+08 | 3.78E+07 | **0** | 0 | 1.11E+09 | 1.27E+08 | **10** | 0 |
|  | OS-Marine | 5.79E+08 | 2.89E+07 | **0** | 0 | 5.43E+08 | 5.60E+07 | **0** | 0 |
|  | SM-Outfall | 7.28E+08 | 6.69E+07 | **5** | 1 | 5.31E+08 | 3.38E+07 | **3** | 1 |
| 25.04.2019 | R-Estuary-1 | 6.59E+08 | 3.19E+07 | **12** | 17 | 4.87E+08 | 3.87E+07 | **20** | 28 |
|  | R-Estuary-2 | 5.98E+08 | 3.43E+07 | **0** | 0 | 5.02E+08 | 2.61E+07 | **1** | 1 |
|  | NS-Marine | 6.08E+08 | 2.02E+07 | **2** | 0 | 6.33E+08 | 2.18E+07 | **4** | 1 |
|  | OS-Marine | 6.06E+08 | 1.34E+08 | **0** | 0 | 4.48E+08 | 1.64E+07 | **0** | 0 |
|  | SM-Outfall | 4.79E+08 | 5.22E+07 | **0** | 0 | 4.50E+08 | 4.73E+07 | **0** | 0 |
| 18.06.2019 | R-Estuary-1 | 2.37E+09 | 3.24E+07 | **1** | 1 | 2.65E+09 | 5.88E+07 | **0** | 0 |
|  | R-Estuary-2 | 2.05E+09 | 5.44E+07 | **5** | 5 | 2.15E+09 | 1.46E+08 | **0** | 0 |
|  | NS-Marine | 1.66E+09 | 2.40E+07 | **1** | 1 | 1.66E+09 | 3.48E+07 | **0** | 0 |
|  | OS-Marine | 2.32E+09 | 8.04E+07 | **0** | 0 | 1.47E+09 | 1.24E+07 | **0** | 0 |
|  | SM-Outfall | 1.59E+09 | 8.91E+06 | **0** | 0 | 1.69E+09 | 2.43E+07 | **0** | 0 |
| 15.10.2019 | R-Estuary-1 | 1.62E+09 | 1.20E+07 | **5** | 7 | 1.99E+09 | 2.45E+07 | **380** | 6 |
|  | R-Estuary-2 | 1.52E+09 | 1.16E+07 | **3** | 1 | 2.26E+09 | 2.47E+07 | **556** | 74 |
|  | NS-Marine | 1.56E+09 | 1.95E+07 | **3** | 1 | 1.53E+09 | 7.93E+06 | **0** | 0 |
|  | OS-Marine | 1.45E+09 | 8.20E+06 | **0** | 0 | 1.43E+09 | 2.53E+07 | **0** | 0 |
|  | SM-Outfall | 1.49E+09 | 1.10E+07 | **0** | 0 | 1.24E+09 | 8.52E+06 | **0** | 0 |

**Supplementary Table 6:** Number of ASVs of bacterial indicators associated with wastewater source (WW = R-Estuary-1, surface sample; SM = SM-Outfall, bottom sample) according to SourceTracker analysis.

| **Phylum** | **Class** | **Order** | **Family** | **WW** | **SM** |
| --- | --- | --- | --- | --- | --- |
| Actinobacteriota | Actinobacteria | Corynebacteriales | *Mycobacteriaceae* | **2** | **NA** |
| Bacteroidota | Bacteroidia | Bacteroidales | *Bacteroidaceae* | **2** | **NA** |
| Bdellovibrionota | Bdellovibrionia | Bdellovibrionales | *Bdellovibrionaceae* | **2** | **3** |
| Campilobacterota | Campylobacteria | Campylobacterales | *Arcobacteraceae* | **18** | **NA** |
| Firmicutes | Clostridia | Lachnospirales | *Lachnospiraceae* | **1** | **NA** |
| Firmicutes | Clostridia | Oscillospirales | *Ruminococcaceae* | **1** | **1** |
| Proteobacteria | Gammaproteobacteria | Aeromonadales | *Aeromonadaceae* | **5** | **NA** |
| Proteobacteria | Gammaproteobacteria | Enterobacterales | *Enterobacteriaceae* | **1** | **NA** |
| Proteobacteria | Gammaproteobacteria | Pseudomonadales | *Moraxellaceae* | **7** | **NA** |
| Proteobacteria | Gammaproteobacteria | Pseudomonadales | *Pseudomonadaceae* | **1** | **NA** |
| Proteobacteria | Gammaproteobacteria | Vibrionales | *Vibrionaceae* | **4** | **5** |
| Spirochaetota | Leptospirae | Leptospirales | *Leptospiraceae* | **1** | **NA** |

**Supplementary Table 7:** ASVs associated reference sequences of known *Arcobacter* species in 2015/2016 and 2018/2019 dataset.

| **Reference sequences** | **Associated ASVs in 2015/2016 dataset** | **Associated ASVs in 2018/2019 dataset** |
| --- | --- | --- |
| *Arcobacter cryaerophilus* | ASV55, ASV897 | ASV55 |
| *Arcobacter nitrofigilis* | ASV522, ASV475, ASV1087, ASV2674, ASV712, ASV5285 | ASV522, ASV475, ASV1087 |
| *Arcobacter suis* | ASV703, ASV684, ASV279 | ASV703, ASV684, ASV279 |
