# Supplementary figures and images for: Bacterial Indicators Are Ubiquitous Members of Pelagic Microbiome in Anthropogenically Impacted Coastal Ecosystem
Source: Front Microbiol. 2022 Jan 17;12:765091. doi: 10.3389/fmicb.2021.765091 (PMC8801744; doi:10.3389/fmicb.2021.765091)

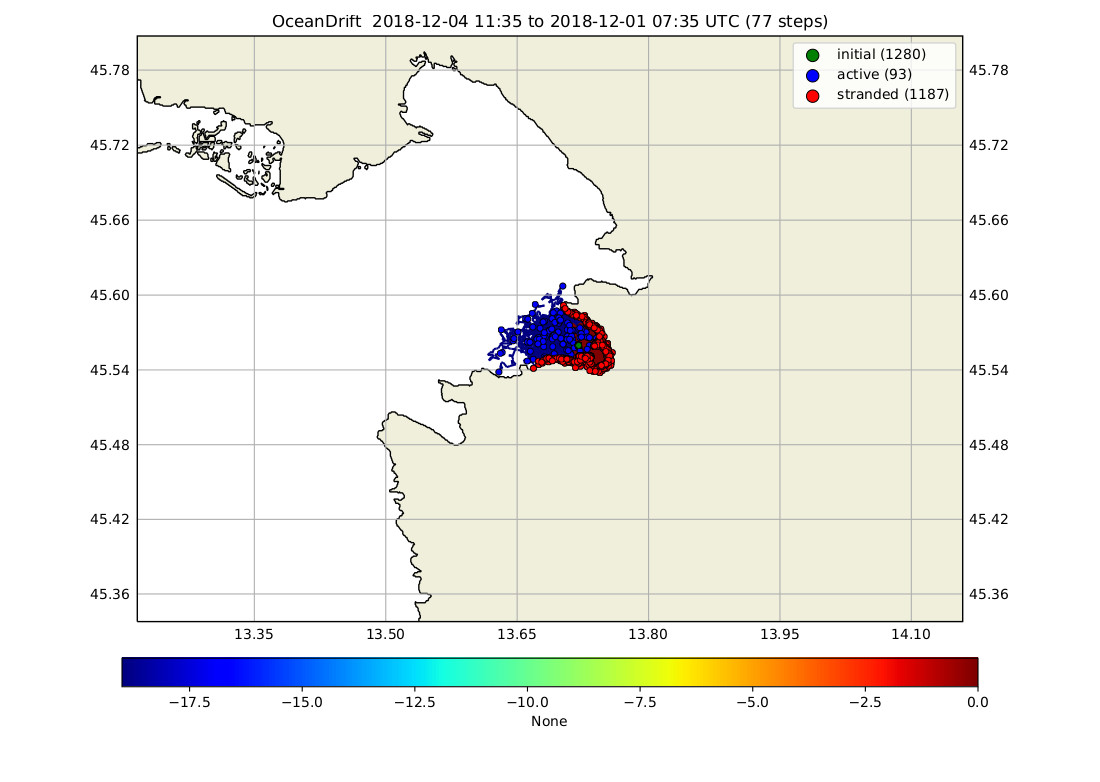

Supplement: Supplementary file 3 [file Data_Sheet_3.ZIP › Supplementary_material_3/Supplementary material 3/opendrift_oceandrift_backprop_z_NS-Marine_2018120411.jpg]

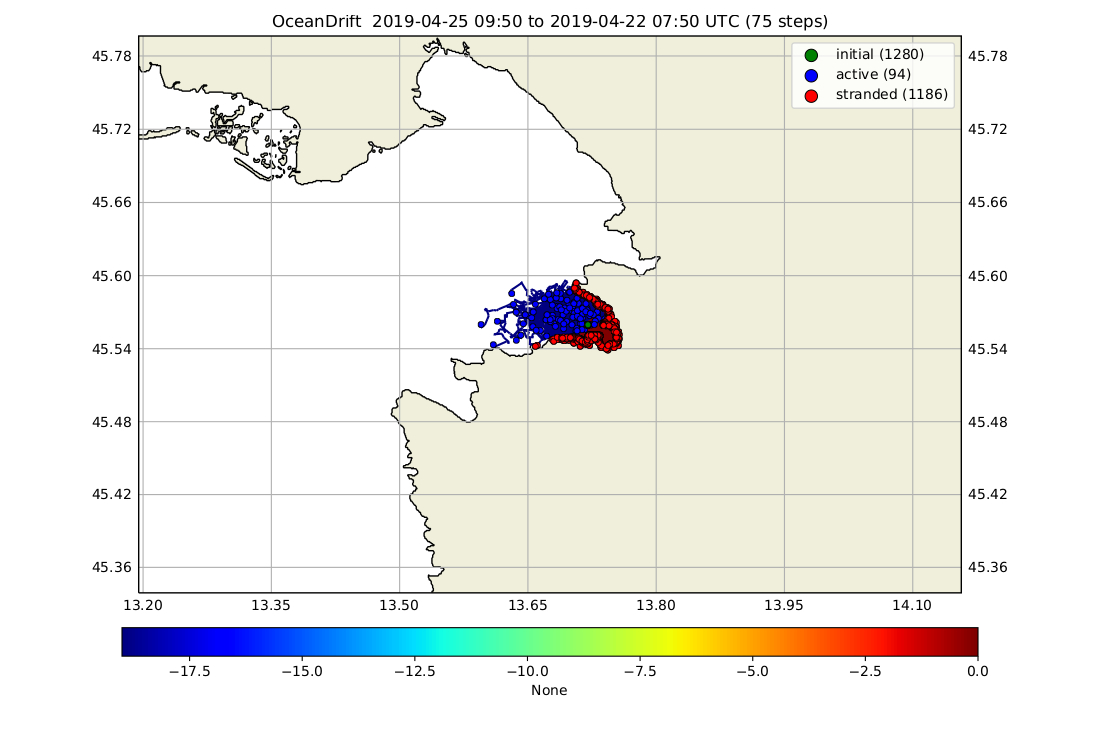

Supplement: Supplementary file 3 [file Data_Sheet_3.ZIP › Supplementary_material_3/Supplementary material 3/opendrift_oceandrift_backprop_z_NS-Marine_2019042509.jpg]

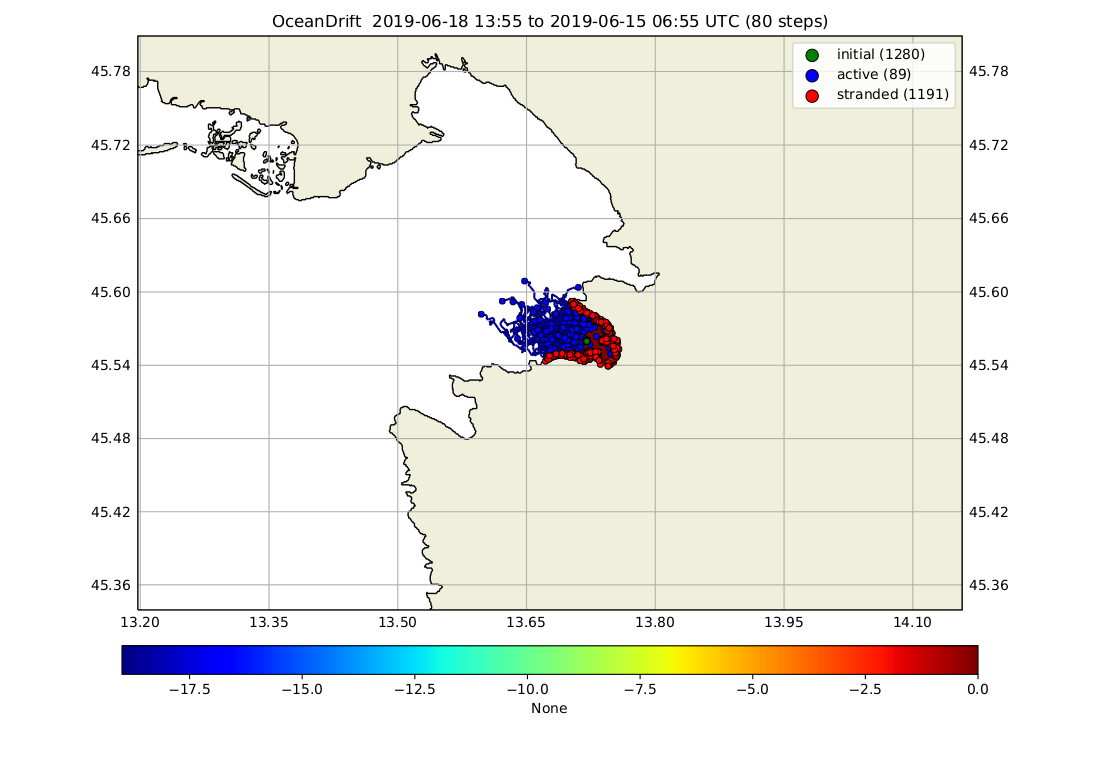

Supplement: Supplementary file 3 [file Data_Sheet_3.ZIP › Supplementary_material_3/Supplementary material 3/opendrift_oceandrift_backprop_z_NS-Marine_2019061813.jpg]

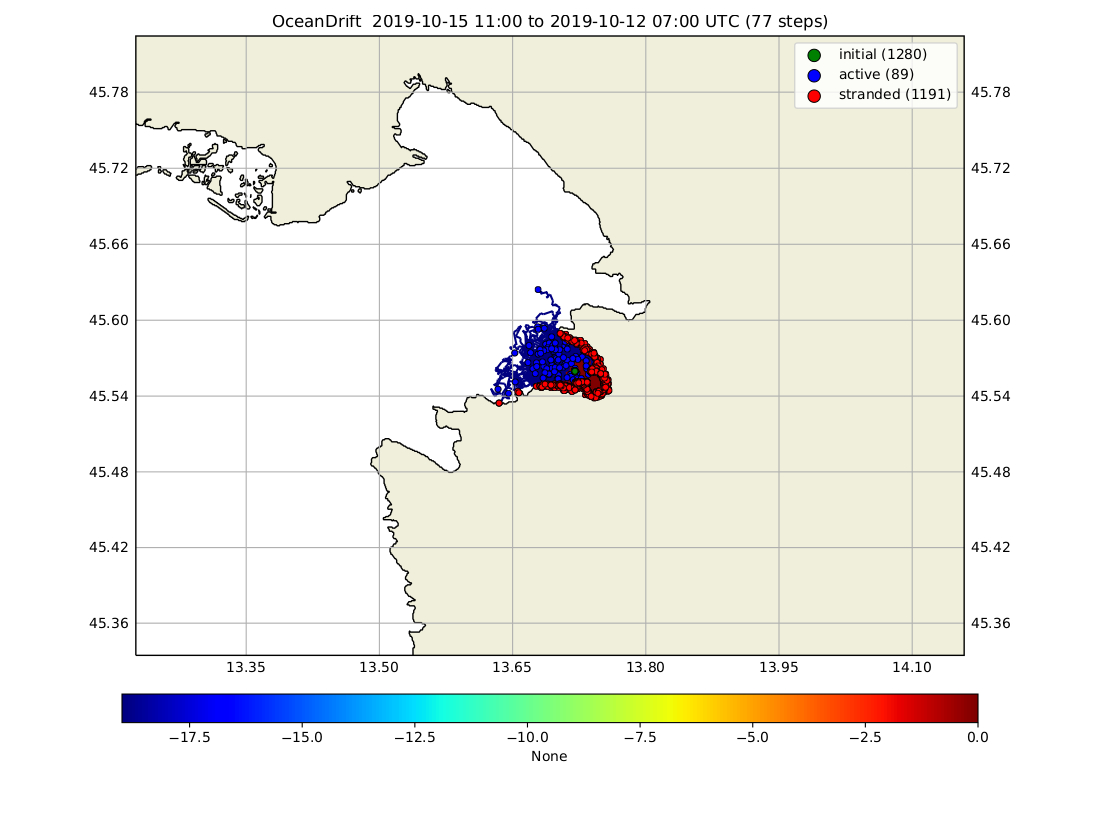

Supplement: Supplementary file 3 [file Data_Sheet_3.ZIP › Supplementary_material_3/Supplementary material 3/opendrift_oceandrift_backprop_z_NS-Marine_2019101511.jpg]

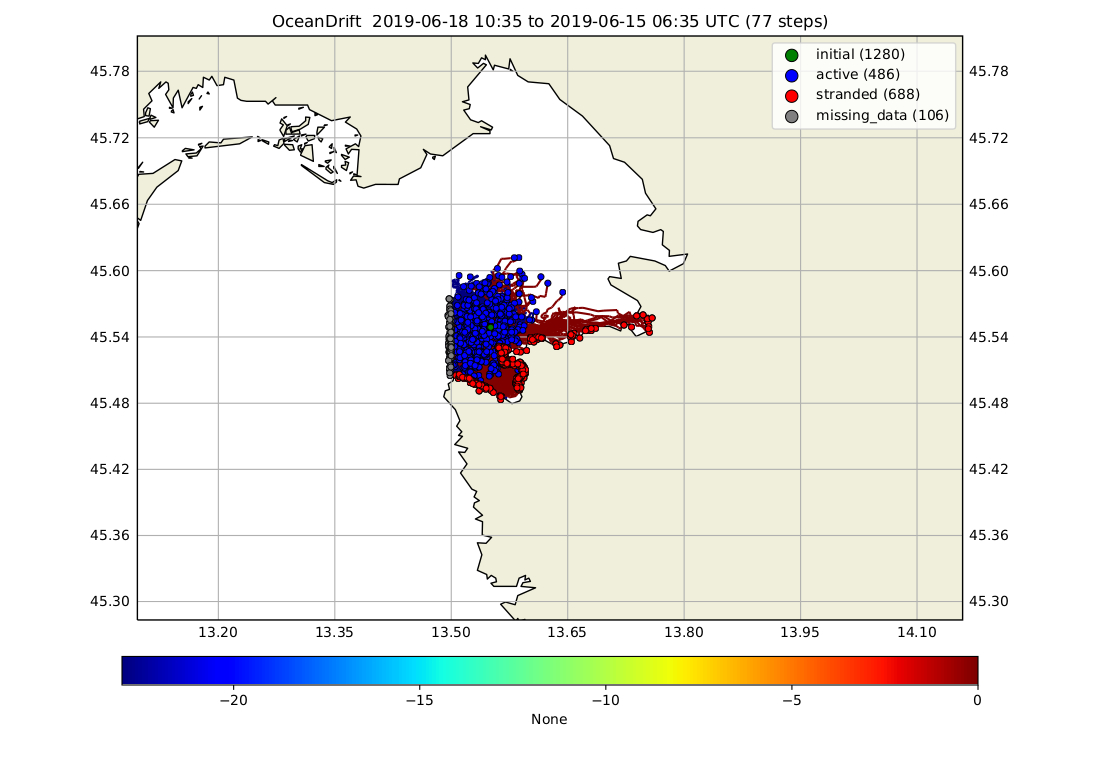

Supplement: Supplementary file 3 [file Data_Sheet_3.ZIP › Supplementary_material_3/Supplementary material 3/opendrift_oceandrift_backprop_z_OS-Marine.jpg]

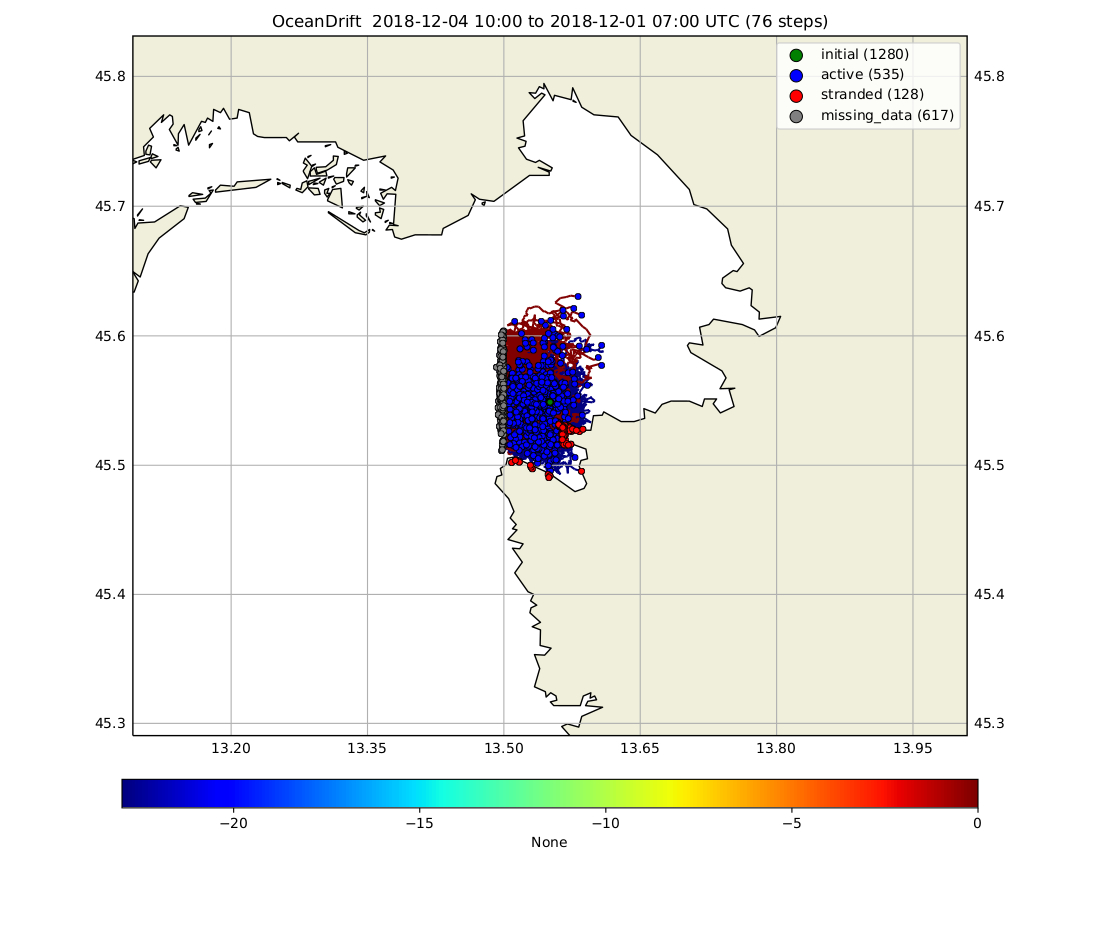

Supplement: Supplementary file 3 [file Data_Sheet_3.ZIP › Supplementary_material_3/Supplementary material 3/opendrift_oceandrift_backprop_z_OS-Marine_2018120410.jpg]

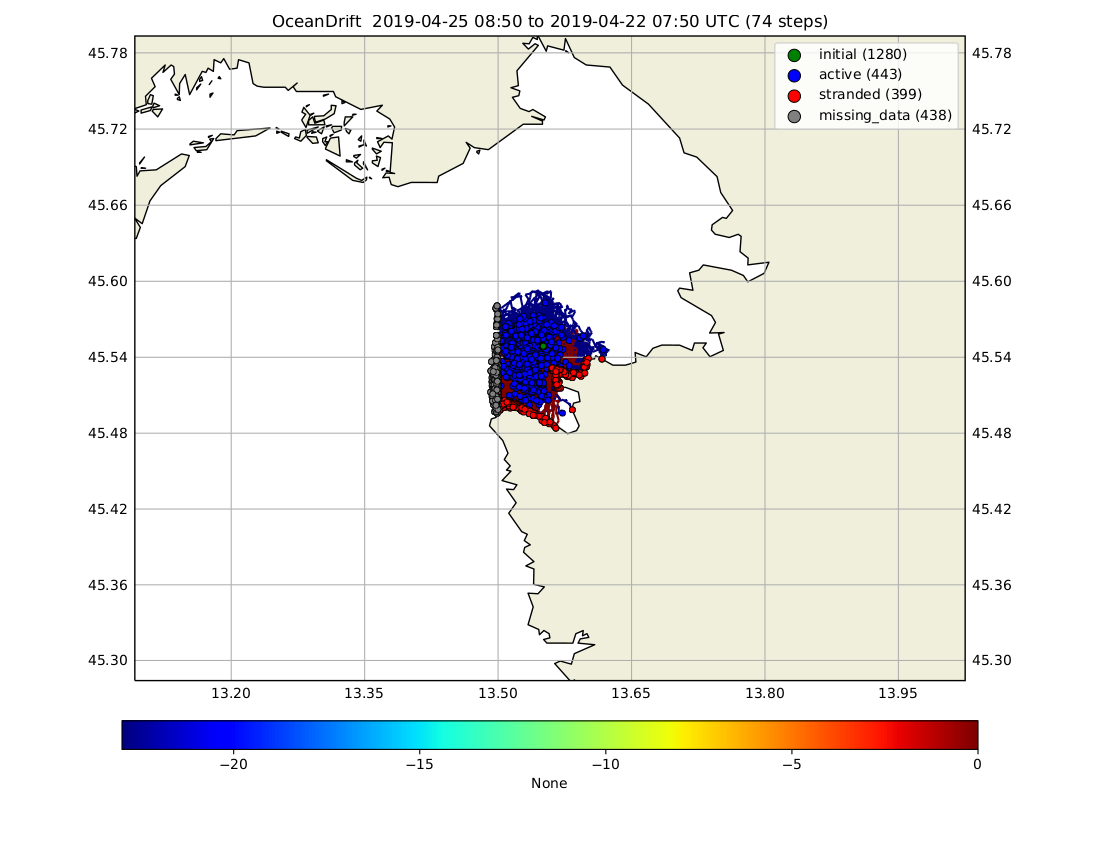

Supplement: Supplementary file 3 [file Data_Sheet_3.ZIP › Supplementary_material_3/Supplementary material 3/opendrift_oceandrift_backprop_z_OS-Marine_2019042508.jpg]

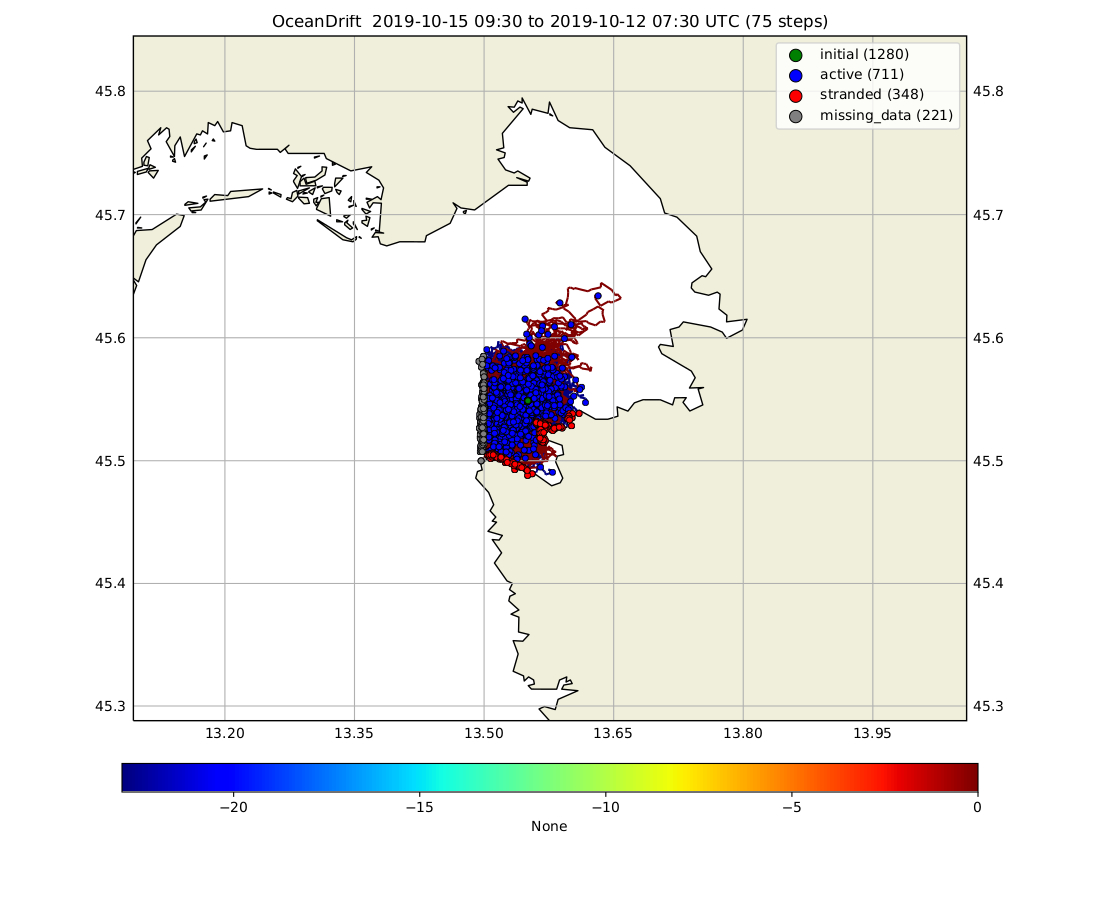

Supplement: Supplementary file 3 [file Data_Sheet_3.ZIP › Supplementary_material_3/Supplementary material 3/opendrift_oceandrift_backprop_z_OS-Marine_2019101509.jpg]

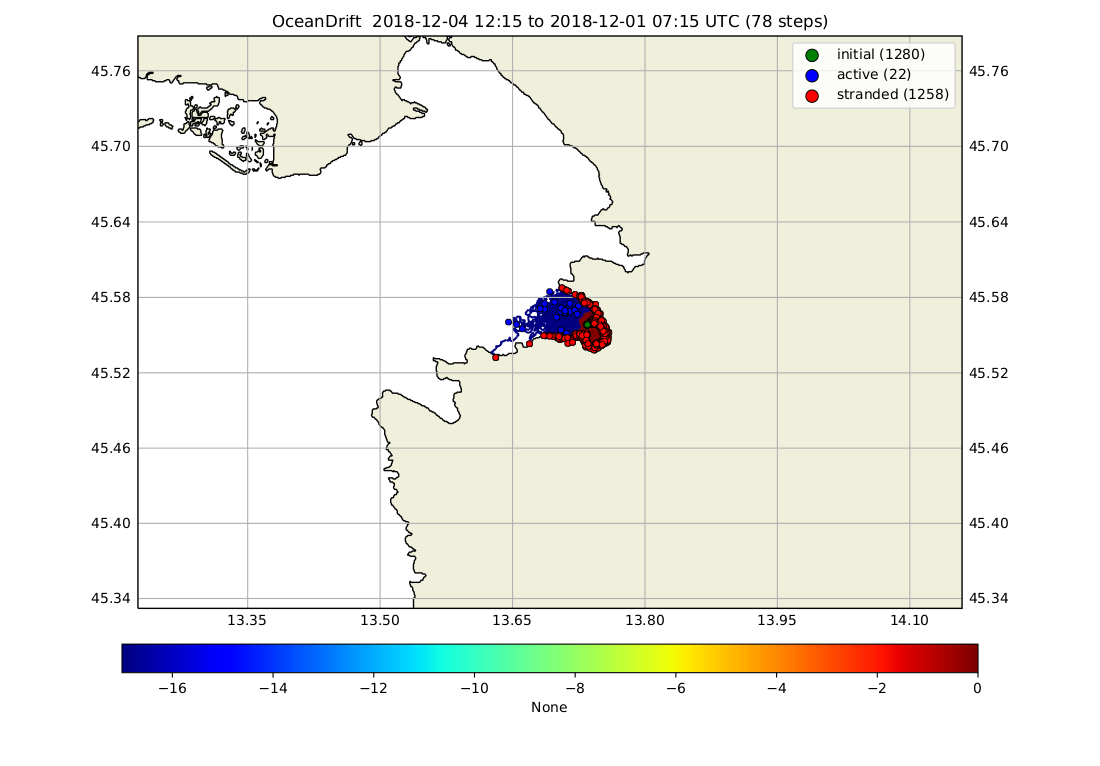

Supplement: Supplementary file 3 [file Data_Sheet_3.ZIP › Supplementary_material_3/Supplementary material 3/opendrift_oceandrift_backprop_z_R-Estuary-1_2018120412.jpg]

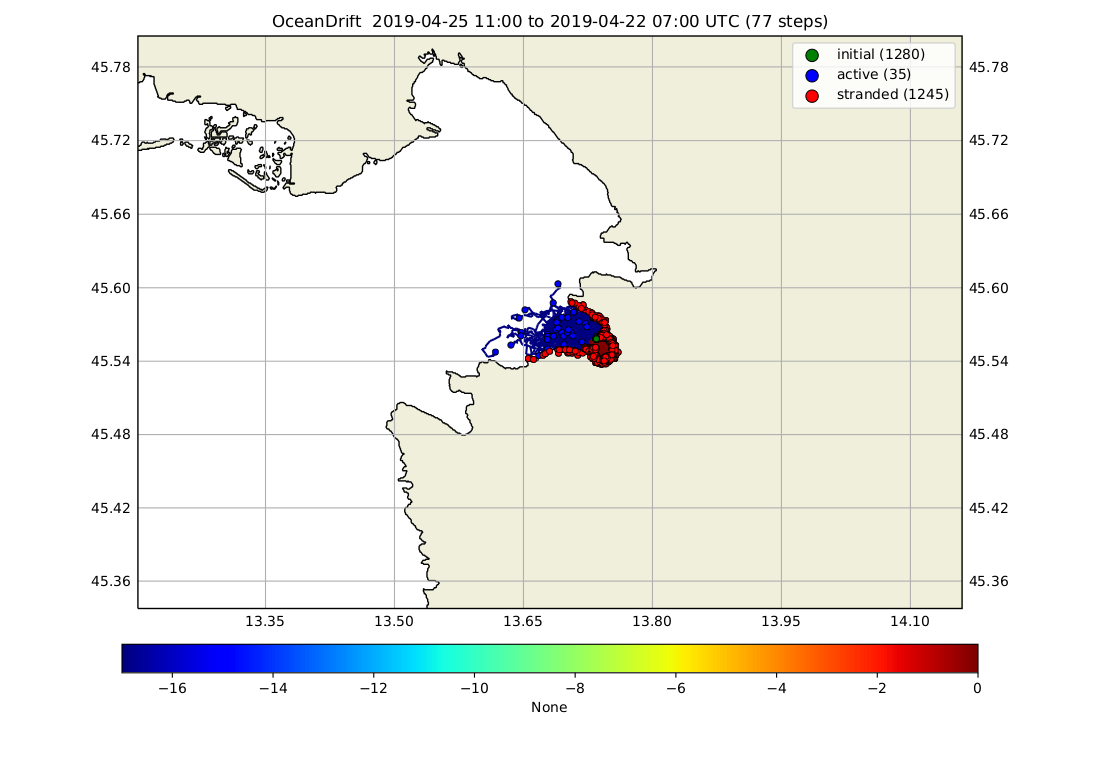

Supplement: Supplementary file 3 [file Data_Sheet_3.ZIP › Supplementary_material_3/Supplementary material 3/opendrift_oceandrift_backprop_z_R-Estuary-1_2019042511.jpg]

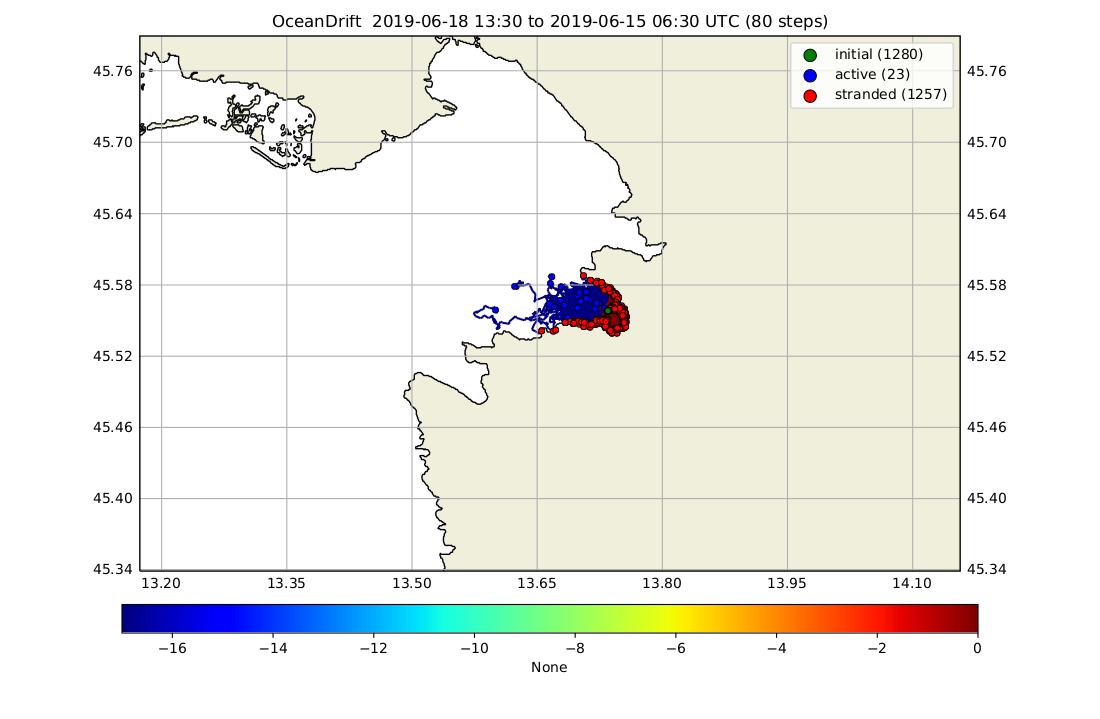

Supplement: Supplementary file 3 [file Data_Sheet_3.ZIP › Supplementary_material_3/Supplementary material 3/opendrift_oceandrift_backprop_z_R-Estuary-1_2019061813.jpg]

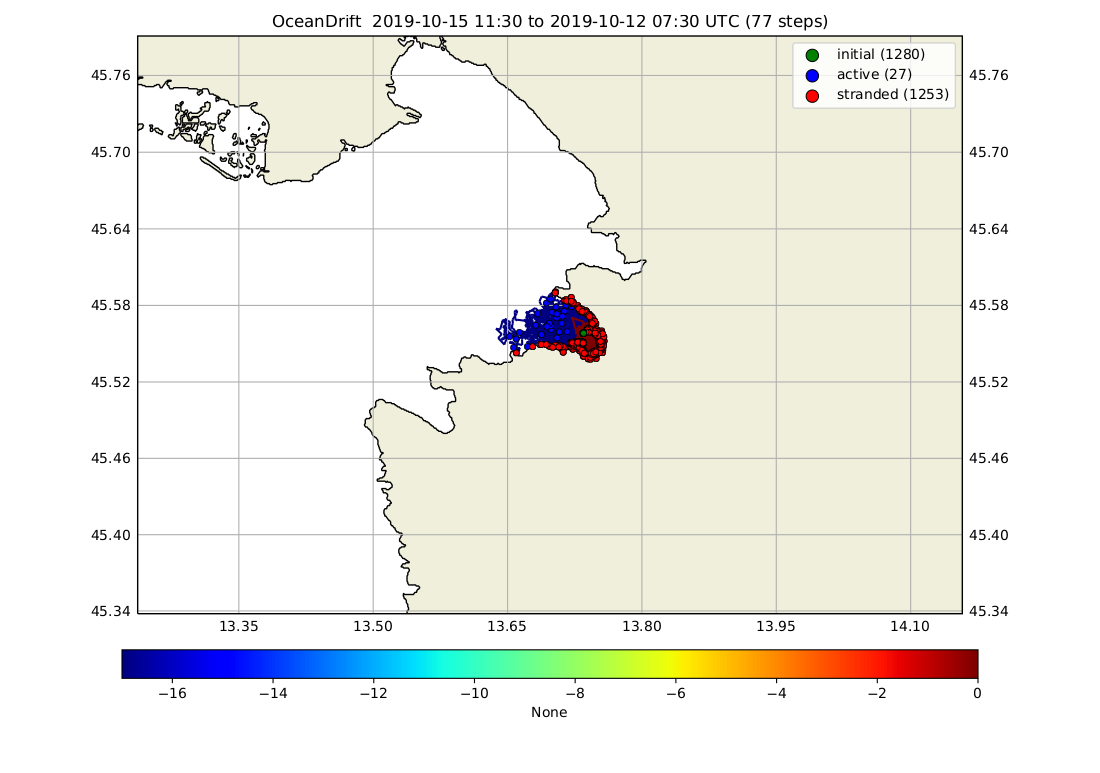

Supplement: Supplementary file 3 [file Data_Sheet_3.ZIP › Supplementary_material_3/Supplementary material 3/opendrift_oceandrift_backprop_z_R-Estuary-1_2019101511.jpg]

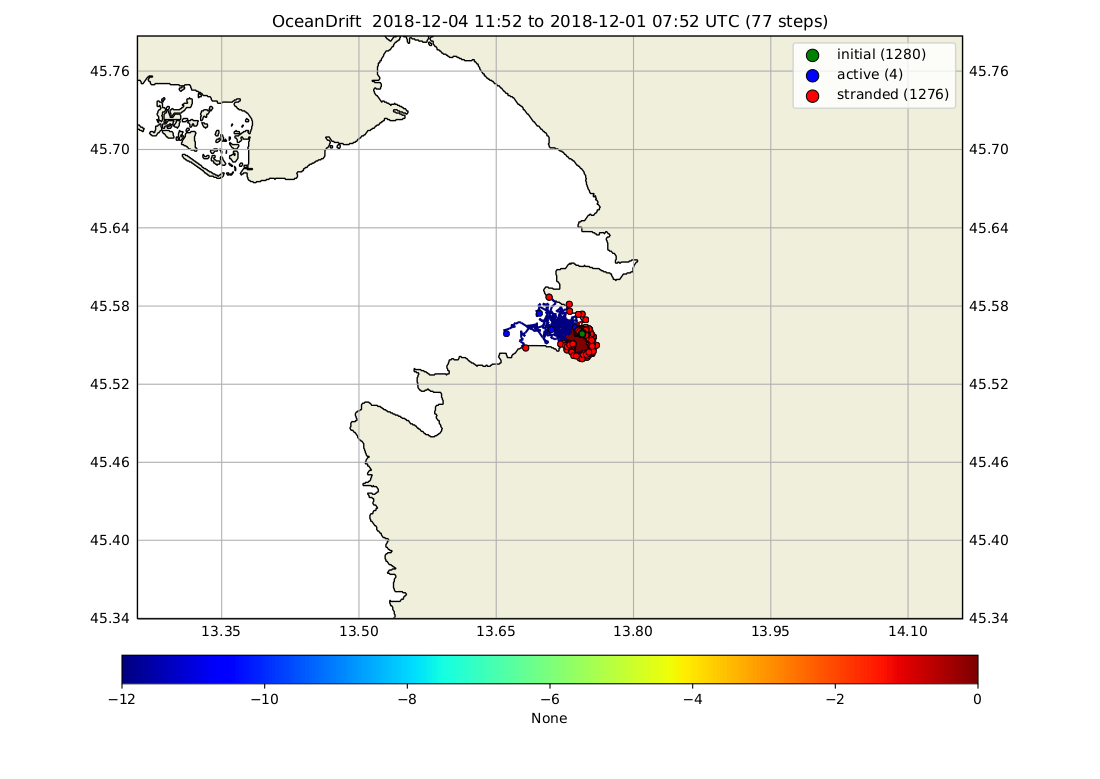

Supplement: Supplementary file 3 [file Data_Sheet_3.ZIP › Supplementary_material_3/Supplementary material 3/opendrift_oceandrift_backprop_z_R-Estuary-2_2018120411.jpg]

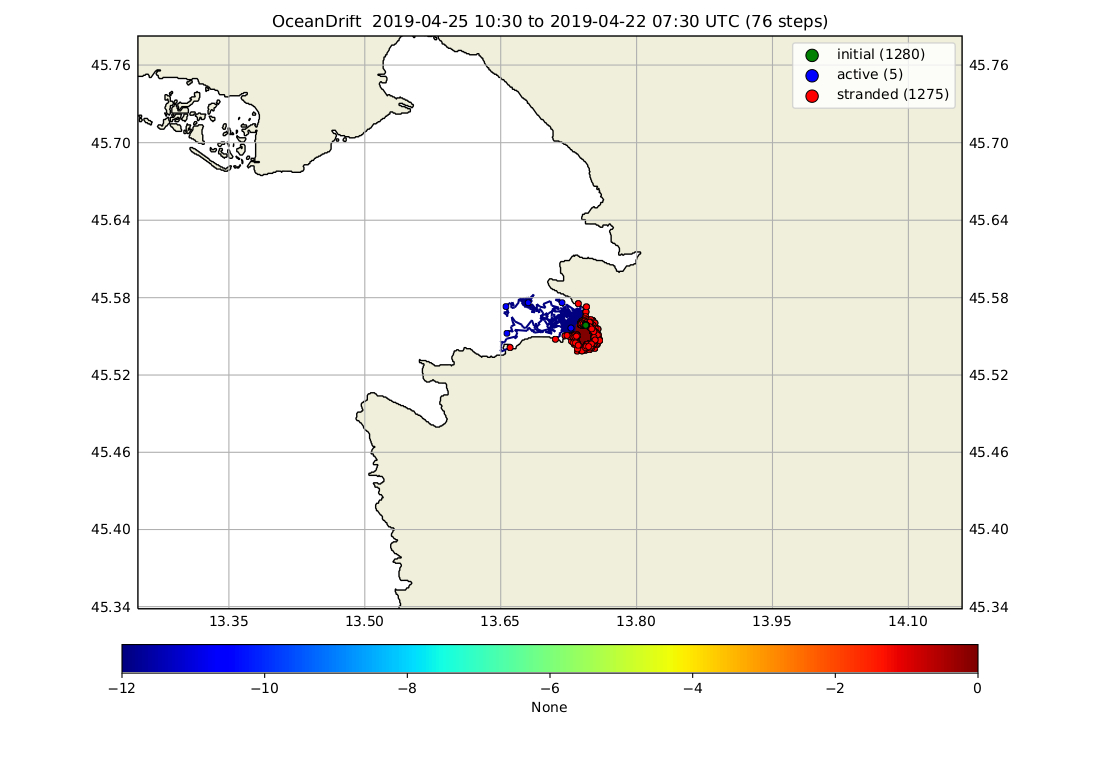

Supplement: Supplementary file 3 [file Data_Sheet_3.ZIP › Supplementary_material_3/Supplementary material 3/opendrift_oceandrift_backprop_z_R-Estuary-2_2019042510.jpg]

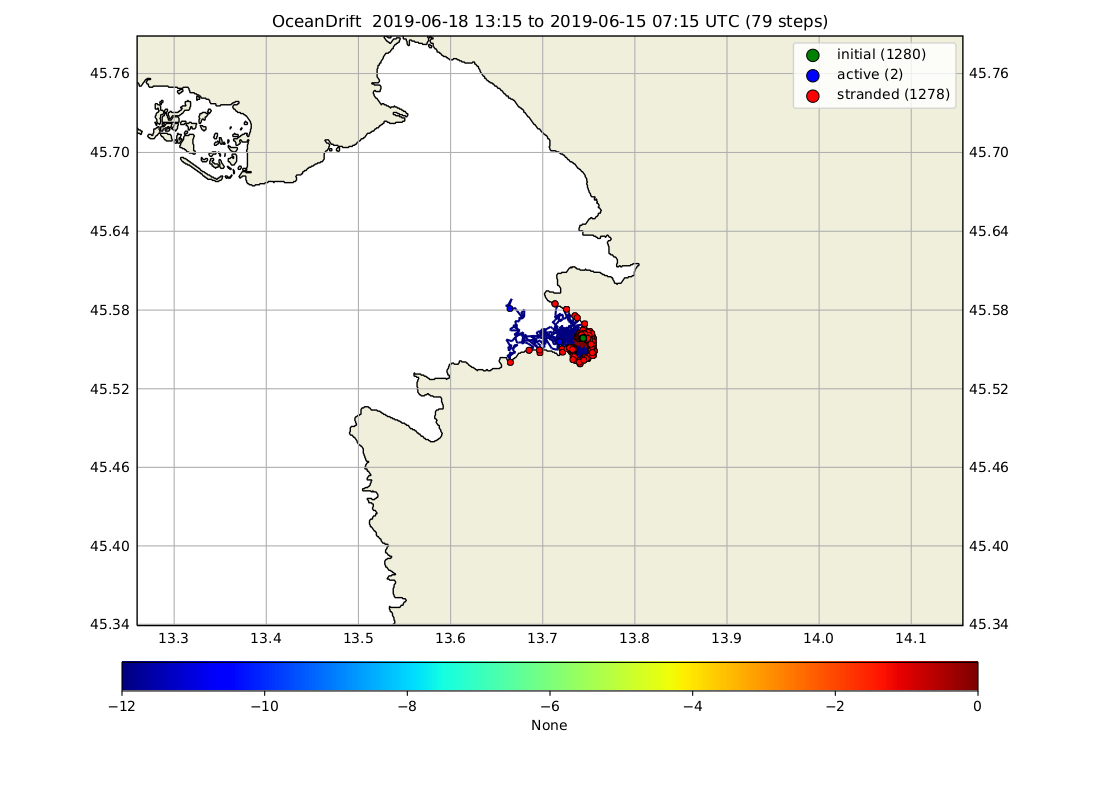

Supplement: Supplementary file 3 [file Data_Sheet_3.ZIP › Supplementary_material_3/Supplementary material 3/opendrift_oceandrift_backprop_z_R-Estuary-2_2019061813.jpg]

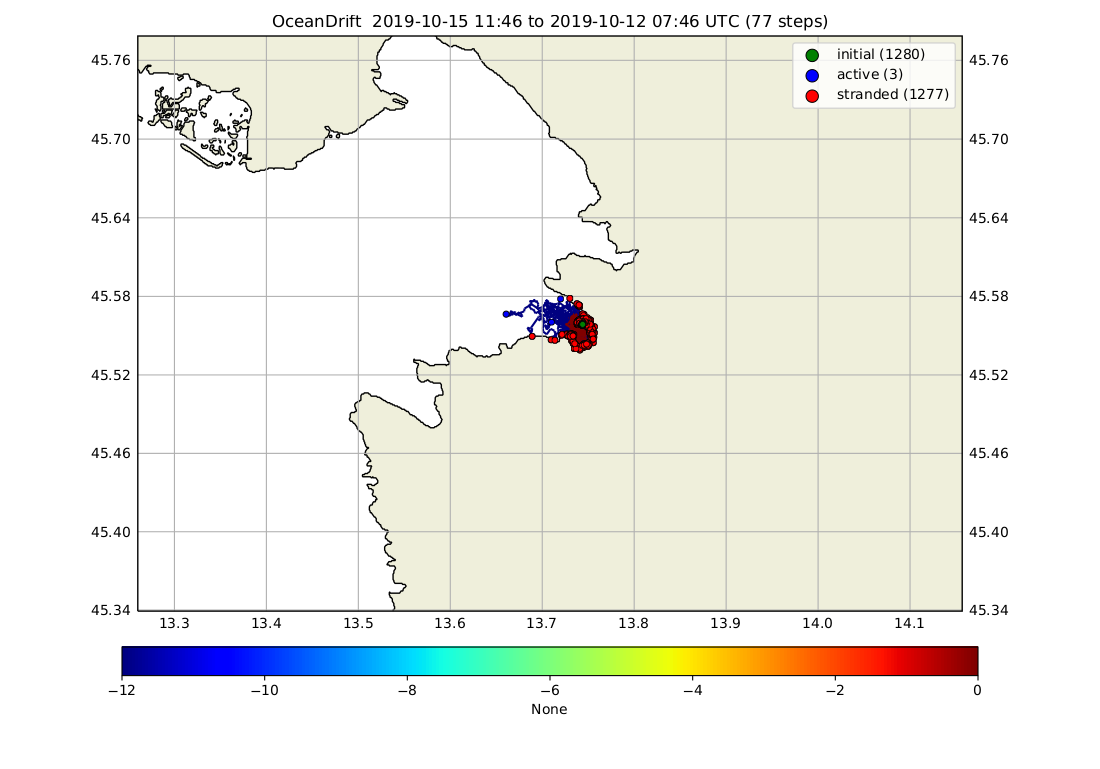

Supplement: Supplementary file 3 [file Data_Sheet_3.ZIP › Supplementary_material_3/Supplementary material 3/opendrift_oceandrift_backprop_z_R-Estuary-2_2019101511.tif]

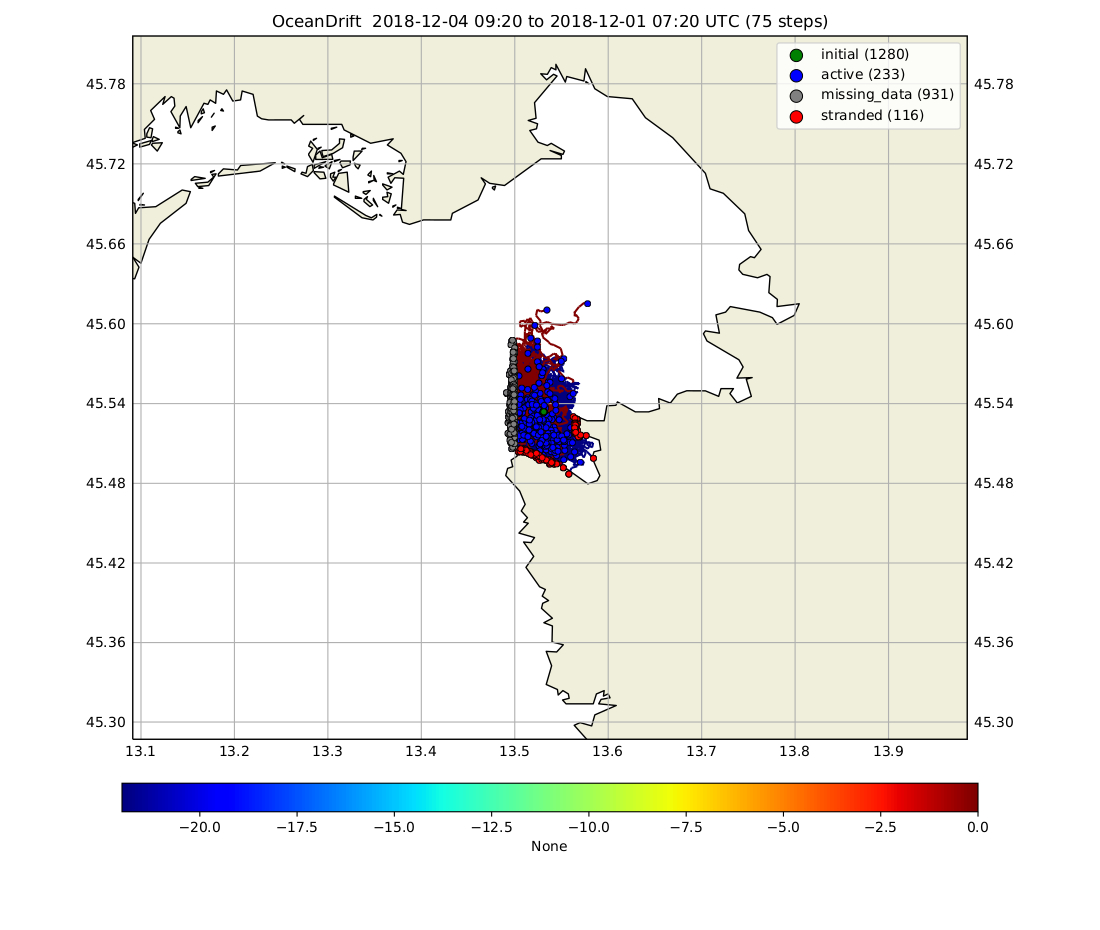

Supplement: Supplementary file 3 [file Data_Sheet_3.ZIP › Supplementary_material_3/Supplementary material 3/opendrift_oceandrift_backprop_z_SM-Outfall_2018120409.jpg]

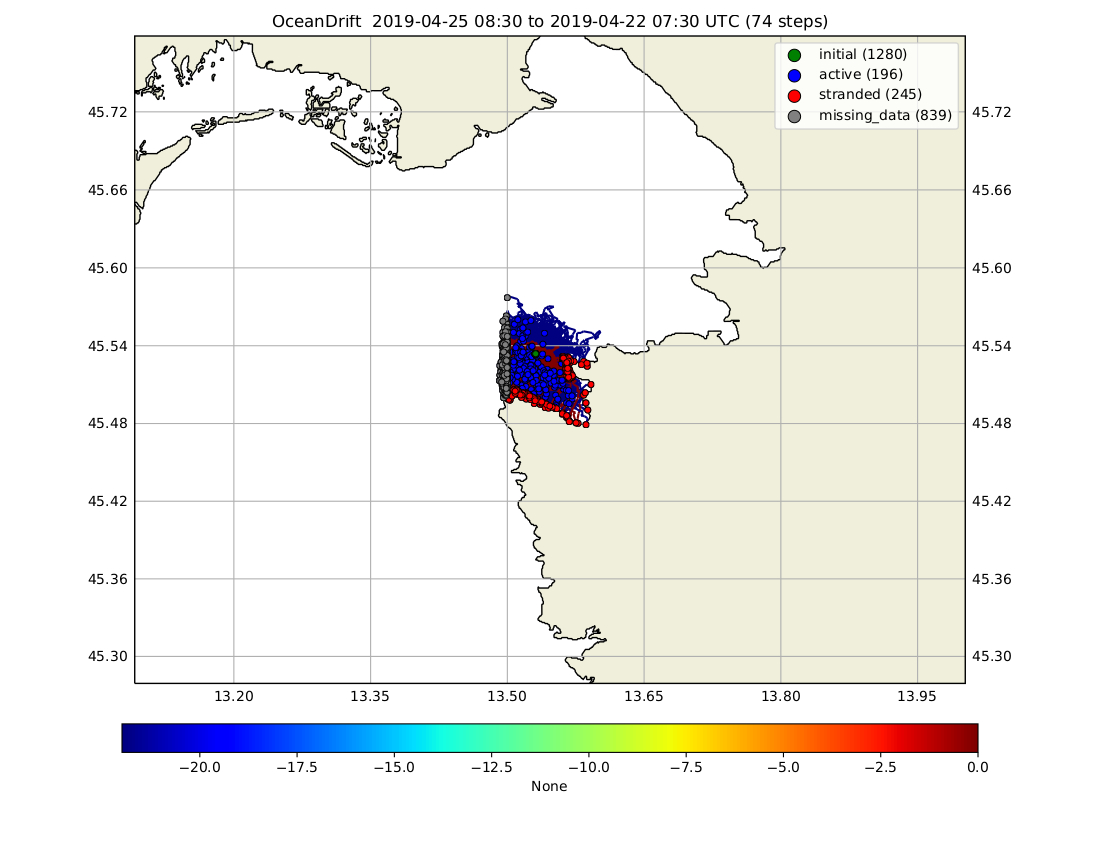

Supplement: Supplementary file 3 [file Data_Sheet_3.ZIP › Supplementary_material_3/Supplementary material 3/opendrift_oceandrift_backprop_z_SM-Outfall_2019042508.jpg]

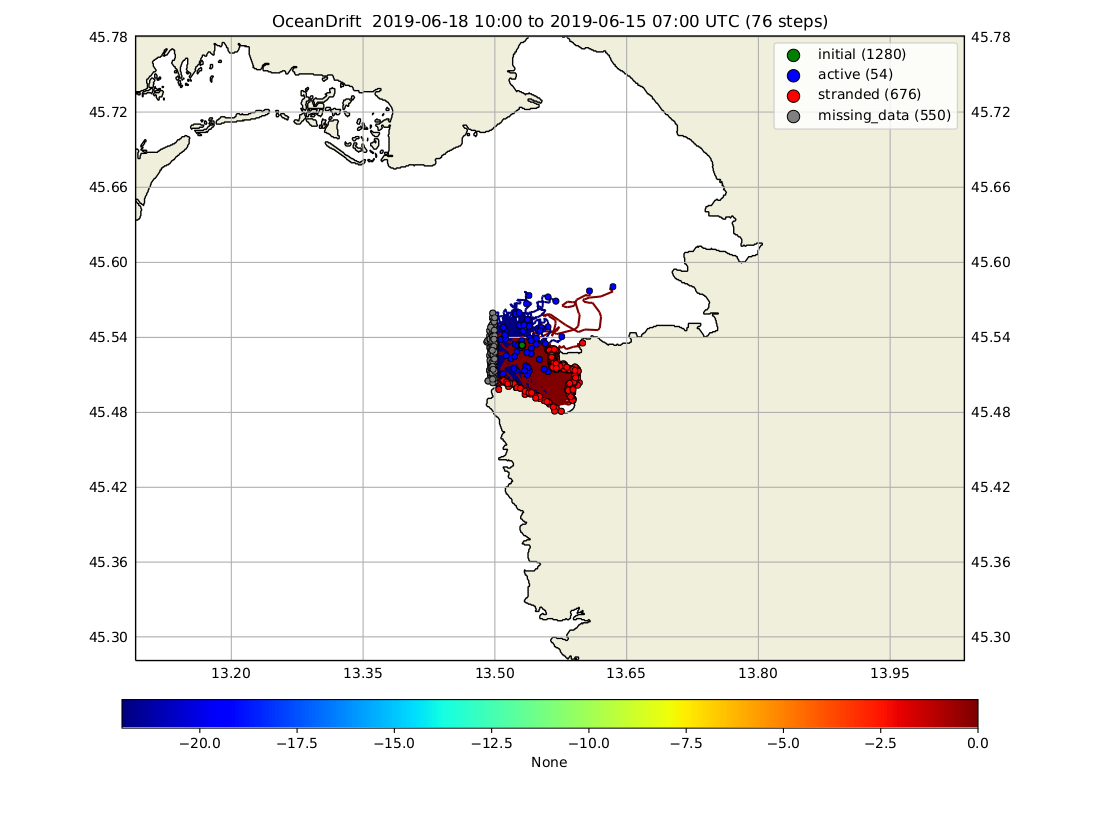

Supplement: Supplementary file 3 [file Data_Sheet_3.ZIP › Supplementary_material_3/Supplementary material 3/opendrift_oceandrift_backprop_z_SM-Outfall_2019061810.jpg]

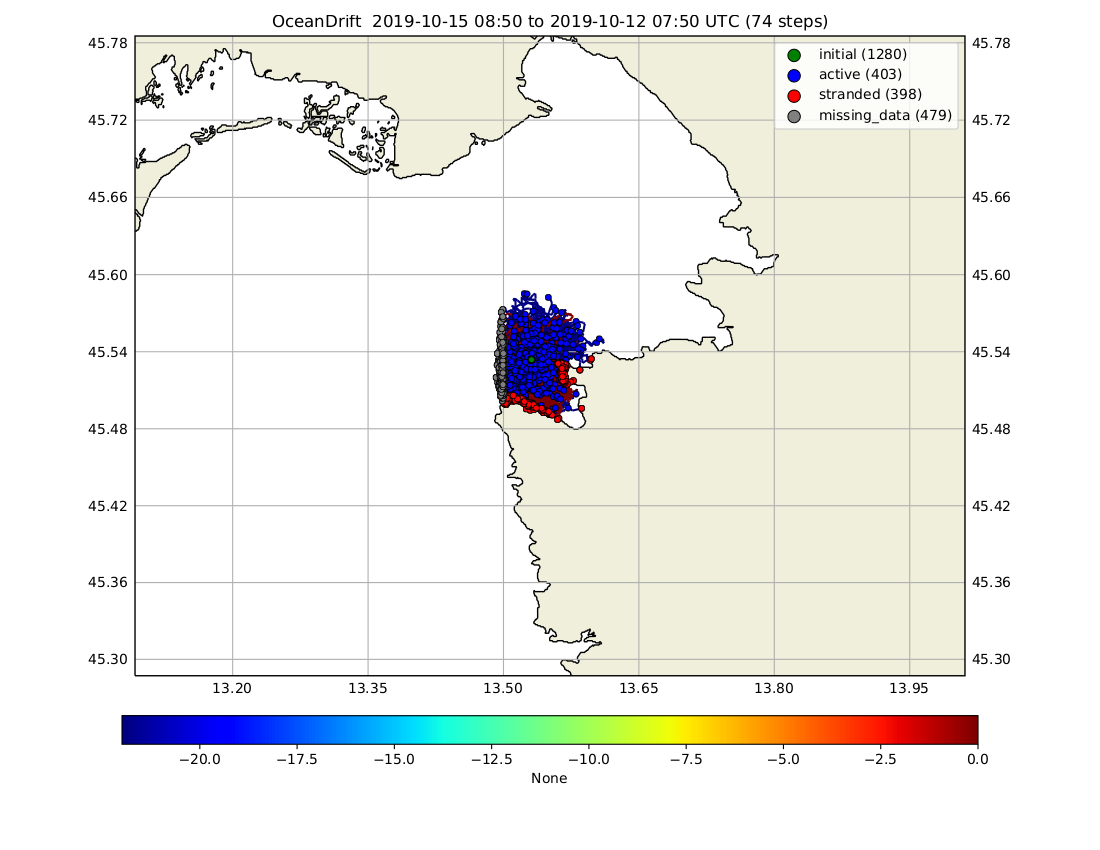

Supplement: Supplementary file 3 [file Data_Sheet_3.ZIP › Supplementary_material_3/Supplementary material 3/opendrift_oceandrift_backprop_z_SM-Outfall_2019101508.jpg]
